# Statistical properties of cerebral near infrared and intracranial pressure-based cerebrovascular reactivity metrics in moderate and severe neural injury: a machine learning and time-series analysis

**DOI:** 10.1186/s40635-023-00541-3

**Published:** 2023-08-28

**Authors:** Alwyn Gomez, Amanjyot Singh Sainbhi, Kevin Y. Stein, Nuray Vakitbilir, Logan Froese, Frederick A. Zeiler

**Affiliations:** 1 Section of Neurosurgery, Department of Surgery, Rady Faculty of Health Sciences, University of Manitoba, Winnipeg, MB Canada; 2 Department of Human Anatomy and Cell Science, Rady Faculty of Health Sciences, University of Manitoba, Winnipeg, Canada; 3 Department of Biomedical Engineering, Price Faculty of Engineering, University of Manitoba, Winnipeg, MB Canada; 4 Centre on Aging, University of Manitoba, Winnipeg, Canada; 5 Division of Anaesthesia, Department of Medicine, Addenbrooke’s Hospital, University of Cambridge, Cambridge, UK; 6 Department of Clinical Neurosciences, Karolinksa Institutet, Stockholm, Sweden

**Keywords:** Cerebrovascular reactivity, Multi-modal monitoring, Near infrared spectroscopy, Traumatic brain injury

## Abstract

**Background:**

Cerebrovascular reactivity has been identified as a key contributor to secondary injury following traumatic brain injury (TBI). Prevalent intracranial pressure (ICP) based indices of cerebrovascular reactivity are limited by their invasive nature and poor spatial resolution. Fortunately, interest has been building around near infrared spectroscopy (NIRS) based measures of cerebrovascular reactivity that utilize regional cerebral oxygen saturation (rSO_2_) as a surrogate for pulsatile cerebral blood volume (CBV). In this study, the relationship between ICP- and rSO_2_-based indices of cerebrovascular reactivity, in a cohort of critically ill TBI patients, is explored using classical machine learning clustering techniques and multivariate time-series analysis.

**Methods:**

High-resolution physiologic data were collected in a cohort of adult moderate to severe TBI patients at a single quaternary care site. From this data both ICP- and rSO_2_-based indices of cerebrovascular reactivity were derived. Utilizing agglomerative hierarchical clustering and principal component analysis, the relationship between these indices in higher dimensional physiologic space was examined. Additionally, using vector autoregressive modeling, the response of change in ICP and rSO_2_ (ΔICP and ΔrSO_2_, respectively) to an impulse in change in arterial blood pressure (ΔABP) was also examined for similarities.

**Results:**

A total of 83 patients with 428,775 min of unique and complete physiologic data were obtained. Through agglomerative hierarchical clustering and principal component analysis, there was higher order clustering between rSO_2_- and ICP-based indices, separate from other physiologic parameters. Additionally, modeled responses of ΔICP and ΔrSO_2_ to impulses in ΔABP were similar, indicating that ΔrSO_2_ may be a valid surrogate for pulsatile CBV.

**Conclusions:**

rSO_2_- and ICP-based indices of cerebrovascular reactivity relate to one another in higher dimensional physiologic space. ΔICP and ΔrSO_2_ behave similar in modeled responses to impulses in ΔABP. This work strengthens the body of evidence supporting the similarities between ICP-based and rSO_2_-based indices of cerebrovascular reactivity and opens the door to cerebrovascular reactivity monitoring in settings where invasive ICP monitoring is not feasible.

**Supplementary Information:**

The online version contains supplementary material available at 10.1186/s40635-023-00541-3.

## Background

Dysfunctional cerebrovascular reactivity has been identified as a significant contributor to secondary injury following traumatic brain injury (TBI), with current guideline-based management paradigms doing little to mitigate this [1–4]. To date, the most prevalent means of continuously monitoring cerebrovascular reactivity at the bedside of critically ill TBI patients is the Pressure Reactivity Index (PRx), which is a continuously updating Pearson correlation coefficient between arterial blood pressure (ABP) and intracranial pressure (ICP) [5, 6].

In PRx, ABP acts as a surrogate for driving pressure while ICP is a surrogate for pulsatile cerebral blood volume (CBV). This reliance on invasively derived ICP measurements has limited the application of PRx to the acute phase of injury, where ICP monitoring is otherwise already indicated. The invasive nature of ICP monitoring further limits the spatial resolution of PRx to a global measure of cerebrovascular reactivity. This is also true of other ICP-based indices of cerebrovascular reactivity, such as the Pulse Amplitude Index (PAx; the Pearson correlation between pulse amplitude of ICP (AMP) and ABP) and RAC (the Pearson correlation coefficient between AMP and cerebral perfusion pressure (CPP), which is the difference between ABP and ICP) [6, 7]. Beyond this, if the subject is not on the steep portion of the pressure/volume curve, it is unclear how ICP may relate to CBV. For this reason, it should also be noted that there is no clear consensus on which metric of cerebrovascular reactivity is optimal; however, ICP-based indices remain the most prevalent in the literature [8].

Fortunately, interest has been building around near infrared spectroscopy (NIRS)-based measures of cerebrovascular reactivity that utilize regional cerebral oxygen saturation (rSO_2_) as a surrogate for pulsatile CBV [9–11]. The NIRS-based parameter rSO_2_ is measured continuously and non-invasively and has the potential for significantly improved spatial resolution [12]. These indices come in two flavors: Cerebral Oxygenation Index (COx) and ABP-based Cerebral Oxygenation Index (COx_a), with CPP and ABP used as surrogates for driving pressure, respectively. Obviously, COx, by its nature, also depends on invasive ICP monitoring; however, COx_a has the potential of being an entirely non-invasive means of continuously measuring cerebrovascular reactivity [13, 14].

Previous work has examined the co-variance relationship between alternative NIRS-based and ICP-based indices of cerebrovascular reactivity [15], and both NIRS-based and ICP-based indices have been found to detect the lower limit of autoregulation in pre-clinical settings [16–18]. However, to date, there has only been one study evaluating the multivariate co-variance pattern between NIRS and ICP-based indices, with limited examination of COx and COx_a specifically. As such, before confidence in the use of COx/COx_a as surrogates for more invasive measures can develop, more details surrounding the statistical properties of rSO_2_, ICP, COx/COx_a and ICP-derived cerebrovascular indices (PRx, PAx, RAC) need to be obtained. In this study, classical machine learning methodologies along with multivariate time-series modeling are utilized to explore and better define the relationship between ICP-based (PRx, PAx, and RAC) and NIRS-based rSO_2_ (COx and COx_a) indices of cerebrovascular reactivity along with their respective measures of pulsatile CBV, ICP and rSO_2_. Additionally, the relationship of indices that utilize ABP versus those that utilize CPP as a surrogate for driving pressure is also examined as a possible avenue to reduce the invasiveness of monitoring cerebrovascular reactivity.

## Methods

### Study design

A retrospective single-center cohort study utilizing prospectively collected high-resolution physiologic data from critically ill TBI patients was performed, with data originally collected between April of 2019 and December of 2022. The data originated from the Winnipeg Acute TBI database which included adult TBI patients admitted to Winnipeg Health Sciences Centre Intensive Care Units (ICU) with invasive ICP and ABP monitoring, as has been reported in recent studies [19–22]. All patients were cared for in line with contemporary ICP- and CPP-based Brain Trauma Foundation guidelines [23, 24]. For this study, only patients with concurrent NIRS-based rSO_2_ were included. Notably, in this cohort, while rSO_2_ was monitored, it was not actively utilized in the management of patients. Additionally, metrics of cerebrovascular reactivity (whether rSO_2_- or ICP-base) were derived following data collection and were not incorporated into patient care.

### Ethical consideration

Data were collected following full approval by the University of Manitoba Biomedical Research Ethics Board (H2017:181, H2017:188, B2018:103, H2020:118) and the Health Sciences Centre Research Impact Committee.

### Data collection

In total, four high-resolution physiologic data streams were utilized; ABP, ICP, as well as left and right rSO_2_. ABP was measured utilizing radial arterial lines while ICP was monitored using intra-parenchymal strain gauge probes (Codman ICP MicroSensor; Codman & Shurtlef Inc., Raynham, MA, USA) placed in the frontal lobe or using external ventricular drains (Medtronic, Minneapolis, MN, USA). rSO_2_ was measured using NIRS monitoring pads placed on the left and right forehead (Covidien INVOS 5100C), when possible, to interrogate the left and right frontal lobes.

ABP and ICP were recorded using analogue-to-digital signal converters (Data Translations, DT9804 or DT9826) while rSO_2_ was recorded from direct digital output from the monitoring device. Data were recorded at a sampling frequency of 100 Hz for ABP and ICP, to capture full waveform signals, and 1 Hz for rSO_2_, due to limitations in the export frequency. This digitized data were linked and stored in time-series using Intensive Care Monitoring (ICM+) software (Cambridge Enterprise Ltd, Cambridge, UK).

Additionally, demographic data, such as age, biologic sex, Marshal computed tomography (CT) score, admission Glasgow Coma Scale (GCS), admission pupil exam, and metabolic parameters; were collected for all patients. This was utilized to better characterize the cohort. Finally, for the purposes of selecting appropriate rSO_2_ signals, radiographic evidence of extravascular blood that might interfere with NIRS signals was noted for the frontal region of each subject. This included the presence of significant acute subdural hematomas, epidural hematomas, cerebral contusions, and scalp hematomas.

### Physiologic data cleaning and processing

Data cleaning and processing was performed using ICM + software. All high-resolution data streams were manually artifact cleared by qualified personnel. Subsequently, for each subject, the AMP, a continuous physiologic parameter, was derived using Fourier analysis of the ICP pulse waveform. ICP, ABP, AMP, and rSO_2_ were then decimated using a 10-s, non-overlapping, moving average filter in a standard practice to eliminate high-frequency signals unrelated to cerebrovascular reactivity [25–28]. CPP was then derived from the difference of the decimated ABP and ICP signals.

The various continuous indices of cerebrovascular reactivity were then derived using 10-s mean values. PRx was derived as a minute-by-minute updating Pearson correlation between ICP and ABP over a 300 s window of paired 10-s mean values. Similarly, PAx and RAC were derived using the correlation between AMP with ABP and CPP, respectively. Finally, COx and COx_a were derived by calculating the correlation between rSO_2_ with CPP and ABP, respectively. This was performed for each side. As all indices were based on Pearson correlations, they ranged from − 1 to 1, with higher values indicating greater disruption in cerebrovascular reactivity. All data streams were exported as both minute-by-minute and 10-s-by-10-s comma separated values (.csv) files.

### Physiological data analysis and statistical methods

#### Overview

The data analysis was performed using R statistical software (Version 4.2.2, R Foundation for Statistical Computing, Vienna, Austria) with the following packages: *forecast, ggplot2, lmtest, MTS, tidyverse, tseries, vars, zoo*. OpenBLAS (Institute of Software, Chinese Academy of Sciences, Beijing, China) was utilized for the Basic Linear Algebra Subprograms (BLAS) and the Linear Algebra Package (LAPACK) to improve multithreaded computational performance. Data streams were further filtered by removing values likely to be artifactual. ABP values less than 0 mmHg and greater than 200 mmHg, ICP values greater than 100 mmHg, and rSO_2_ values less than 25% were all removed. Additionally, for each subject, the right-sided channel of rSO_2_ used was selected unless a scalp hematoma or frontal contusion was present based on radiographic data, in which case the left side was used. While arguments might be made as to why it may be beneficial to select one side over another, ultimately, in the setting of an exploratory analysis, it was felt that a systematic and consistent approach minimized the possibility of bias. The right side was selected as it is typically the side of ICP monitor placement. Further to this, an exploration of hemispheric difference was felt to be beyond the scope of this study. Finally, for statistical tests alpha was set to 0.05 without correction for multiple comparisons due to the exploratory nature of this study.

Given the nature of this data, the priors of linearity are not upheld as datapoints are not truly independent of one another. As a result, correlation analysis of the data streams was not felt to be appropriate as interpretation of this would be questionable. Following analysis previously reported in the multimodal monitoring literature [15, 29–32], three approaches were taken to characterize the relationship between rSO_2_- and ICP-based indices. First classical unsupervised machine learning methods, agglomerative hierarchical clustering and principal component analysis, were utilized to better understand the relationship between various indices in a multidimensional physiological space. Secondly, multivariate time-series modeling, in the form of vector autoregressive modeling and impulse response functions, was used to model the nature of the changes in ICP (ΔICP) and rSO_2_ (ΔrSO_2_) in response to an impulse of change in ABP (ΔABP). This was done to evaluate if ΔrSO_2_ would respond similarly to ΔICP, as would be expected if rSO_2_ was an appropriate surrogate of pulsatile CBV. The details of these analyses are presented in the following sections. Finally, Granger causality testing was performed to examine the temporal causal relationship between ABP, ICP and rSO_2_. This was done to further evaluate if ABP had a strong temporal causal relationship with both ICP and rSO_2_.

The detailed statistical background of these methodologies is beyond the scope of this paper and the interested reader is directed to recent reviews and textbooks on these topics [32–37]. In brief, agglomerative hierarchical clustering is a means of clustering data points in a multidimensional space based on Euclidian distance. Dendrograms are produced for this analysis that display the relative proximity of data points with those connected at lower levels being more similar than those connected at higher levels. Principal component analysis is a means of dimensionality reduction that identifies vectors (typically 2) in multidimensional space that explain the greatest degree of variance in the data. A biplot can then be made to examine how various parameters project onto this new two-dimensional space. When parameters project closely, they can be thought to have a greater relationship to one another in multidimensional space. Vector autoregressive modeling is a means of modeling a multivariate time-series. That is to say, the relationship between time-series within a collection of time-series. Impulse response function plots can be made from these vector autoregressive models to demonstrate how one time-series may react to an impulse in another. If two series have similar responses to an impulse in a third series, then those two series may be thought to share similar properties. Finally, Granger causality testing is a means of testing the temporal causal relationship of time-series A on time-series B. Fundamentally, the test works by comparing predictions of time-series B based on past values of time-series B with predictions of time-series B based on past values of time-series B and time-series A. If the latter is significantly better statistically then time-series A is said to have a Granger causal relationship with time-series B. It should be noted that this is not necessarily equivalent to true causality.

#### Agglomerative hierarchical clustering and principal component analysis

The high-frequency minute-by-minute data streams of interest (ABP, ICP, CPP, rSO_2_, PRx, PAx, RAC, AMP, COx, and COx_a) were aggregated over all patients in the cohort. Next, due to the nature of agglomerative hierarchical clustering and principal component analysis, any measurements with missing parameters were removed to create a fully populated matrix without missing values. Next, to negate differences in the magnitude of the various physiologic parameters, each parameter was scaled over the entire cohort to have a mean of zero and a standard deviation of one.

To perform agglomerative hierarchical clustering, a Euclidian distance matrix was produced for the scaled parameter matrix indicating the Euclidian distance between each measurement in multidimensional physiologic space. Agglomerative hierarchical clustering was then performed utilizing a complete-linkage clustering methodology. To summarize the results of the agglomerative hierarchical clustering, a dendrogram was plotted indicating the hierarchical relationships between the various physiologic parameters, including ICP- and rSO_2_-based measures of cerebrovascular reactivity. Finally, in order to test the goodness of fit of the clustering, the cophenetic correlation coefficient was calculated.

Principal component analysis was performed on the scaled parameter matrix as an alternative means of evaluating the relationships between various physiologic parameters. A cumulative Scree plot was made to examine the variance in the data explained by each principal component. Finally, a biplot was made using the first and second principal components, PC1 and PC2, as a qualitative means of examining the relationships between the various physiologic parameters, including ICP- and rSO_2_-based measures of cerebrovascular reactivity.

#### Vector autoregressive modeling and impulse response function

The high-frequency 10-s-by-10-s data streams of interest (ABP, ICP, and rSO_2_) were utilized as the cerebral vasoactive response was being examined in this analysis and acts on a frequency scale of approximately 0.1 Hz [27, 28]. Prior to creating a tri-variate (ABP, ICP, and rSO_2_) vector autoregressive model for the cohort, it was imperative that the stationarity of the time-series be determined. First, gaps in the data streams were filled using linear interpolation using the *na.approx()* function in R. Stationarity was examined through the augmented Dickie Fuller and Kwiatkowski–Phillips–Schmidt–Shin tests for stationarity, which generally found ABP, ICP, and rSO_2_ to be non-stationary in most subjects. As such, the first differences of the time-series were taken for each subject. This resulted in the parameters ΔABP, ΔICP, and ΔrSO_2_ which were found to be stationary in almost all subjects augmented Dickie Fuller and Kwiatkowski–Phillips–Schmidt–Shin testing. As such, these parameters were used going forward for vector autoregressive modeling and generation of the impulse response functions.

To determine the appropriate autoregressive order of the vector autoregressive model, the Akaike information criterion was determined for vector autoregressive models of order 1–15. By plotting these values, it was clear that there were diminishing improvements in model quality beyond lag 5 and so to follow the principle of parsimony, a tri-variate vector autoregressive model with autoregressive order of 5 was created using the *VAR()* function in R. Finally, using the *irf()* function in R, this tri-variate vector autoregressive model was utilized to model and plot the response in ΔICP as well as ΔrSO_2_ of an orthogonal impulse in ΔABP over the subsequent 10 lags. Vector autoregressive models and impulse response functions plots were also constructed for individual subjects to examine this relationship on an individual subject basis.

#### Granger causality testing

The 10-s-by-10-s data streams of ΔABP, ΔICP and ΔrSO_2_ were utilized for Granger causality testing. For each subject, the Granger causality of ΔABP → ΔrSO_2_, ΔABP → ΔICP, ΔrSO_2_ → ΔABP, and ΔICP → ΔABP. A time lag of 1 was utilized for all testing. F-statistics and p-values were tabulated for comparison to determine the causal direction.

## Results

### Cohort demographics

In total, 83 patients were included in this study, with 428,775 min of unique physiologic data. The demographic data of this cohort can be seen summarized in Table 1. Of note, in 16 subjects the right-sided rSO_2_ signal was not used due to either the presence of a frontal scalp hematoma or a frontal contusion. In these subjects, the left-sided rSO_2_ signal was used.

**Table 1:** Cohort demographics

Demographic parameter		Median (IQR) or *N* (%)
Age		42 (28.5–60.5)
Male patients		66 (79.5)
Admission GCS	Total	6 (4–8)
	Eye	1 (1–2)
	Verbal	1 (1–2)
	Motor	4 (2–5)
Admission pupil exam	Bilaterally unreactive	13 (15.7)
	Unilaterally Unreactive	16 (19.3)
	Bilaterally reactive	54 (65.1)
Admission Marshall CT score	I	0 (0.0)
	II	3 (3.6)
	III	23 (27.7)
	IV	﻿16 (19.3)
	V	41 (49.3)
	VI	﻿0 (0.0)
ICP monitoring method	Intraparenchymal probe	﻿78 (94.0)
	Extraventricular drain	5 (6.0)
Admission HgB (g/L)		135 (113–147)
Admission serum glucose (mmol/L)		8.05 (7–10.95)
Average PaO_2_ (mmHg) over course of recording		109 (87–138)
Average PaCO_2_ (mmHg) over course of recording		37 (34–40)
Average blood gas pH over course of recording		7.43 (7.39–7.47)
Side of rSO_2_ used	Right	67 (80.7)
	Left	16 (19.3)
Frontal contusion present	Right	9 (10.8)
	Left	7 (8.4)
Frontal scalp hematoma present	Right	7 (8.4)
	Left	﻿6 (7.2)

*CT* computed tomography, *GCS* Glasgow Coma Scale, *HgB* hemoglobin, *ICP *intracranial pressure, *IQR* interquartile range, *N* number of subjects, *PaCO*_*2*_ partial pressure of carbon dioxide in arterial blood, *PaO*_*2*_ partial pressure of oxygen in arterial blood, *rSO*_*2*_ regional cerebral oxygen saturation

### Agglomerative hierarchical clustering

**Fig. 1:**
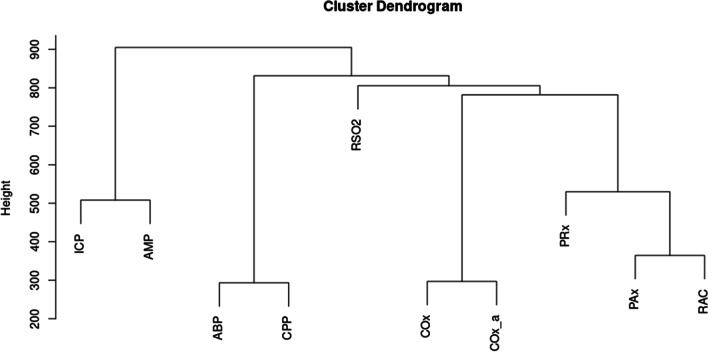
Cluster dendrogram of the minute-by-minute cohort high-resolution physiologic data. *ABP* arterial blood pressure, *AMP* pulse amplitude of ICP, *Cox* rSO_2_ and CPP-based cerebrovascular reactivity index, *COx_a* rSO_2_ and ABP-based cerebrovascular reactivity index, *CPP* cerebral perfusion pressure, *ICP* intracranial pressure, *Pax* AMP and ABP-based cerebrovascular reactivity index, *PRx* ICP and ABP-based cerebrovascular reactivity index, *RAC* AMP and CPP-based cerebrovascular reactivity index, *RSO2* regional cerebral oxygen saturation

The dendrogram from the agglomerative hierarchical clustering can be seen in Fig. 1. The cophenetic correlation was excellent, with a value of 0.94, indicating a good fit of the agglomerative hierarchical clustering. There are several notable features. Firstly, as expected, the various indices of cerebrovascular reactivity (PRx, PAx, and RAC) are closely related based on the early connection of these parameters at lower levels. Perhaps more interestingly, the two rSO_2_-based indices, COx and COx_a, are also closely related with a connection at lower levels. Additionally, ICP-based and rSO_2_-based measures of cerebrovascular reactivity were more closely related to one another than to other physiological parameters, such as ICP, ABP, CPP and rSO_2_ which were only connected to measures of cerebrovascular reactivity late at higher levels.

### Principal component analysis

**Fig. 2:**
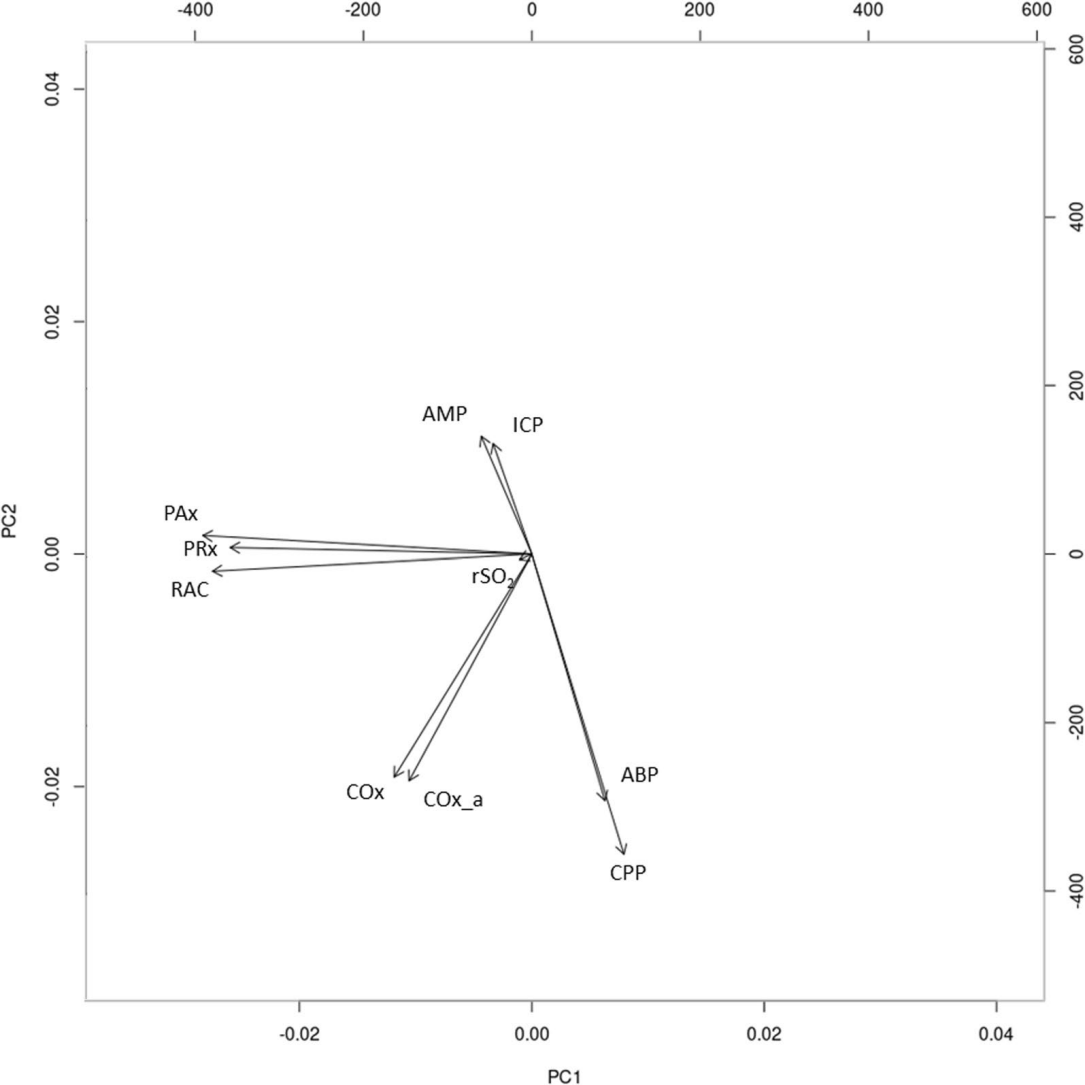
A biplot of the of the minute-by-minute cohort high-resolution physiologic data including principal component 1 (PC1) and principal component 2 (PC2). *ABP* arterial blood pressure, *AMP* pulse amplitude of ICP, *Cox* rSO_2_ and CPP-based cerebrovascular reactivity index, *COx_a* rSO_2_ and ABP-based cerebrovascular reactivity index, *CPP* cerebral perfusion pressure, *ICP* intracranial pressure, *Pax* AMP and ABP-based cerebrovascular reactivity index, *PRx* ICP and ABP-based cerebrovascular reactivity index, *RAC* AMP and CPP-based cerebrovascular reactivity index, *rSO*_*2*_ regional cerebral oxygen saturation

The cumulative Scree plot, and associated data, of the principal component analysis can be found in Additional file 1 with almost 44% of the variance in the data accounted for by the first two principal components. The biplot of the principal component analysis using PC1 and PC2 can be seen in Fig. 2. Similar relationships as in the agglomerative hierarchical clustering dendrogram are seen, with ICP-based indices projected close to one another, indicating a close relationship in multidimensional space. In a similar fashion, COx and COx_a are also projected close to one another. PRx, PAx, and RAC are almost orthogonal to the more traditional physiological parameters of ABP, ICP, and CPP. Furthermore, PRx, PAx, and RAC are more closely located to COx/COx_a in terms of co-variance patterns, indicating that these ICP-based and rSO_2_-based indices are associated in multidimensional physiologic space.

### Vector autoregressive modeling and impulse response function

Initial testing for stationarity of 10-s-by-10-s data streams of ABP, ICP, and rSO_2_, for each subject using the Kwiatkowski–Phillips–Schmidt–Shin, indicated non-stationarity in almost all data streams and all subjects. Augmented Dickie Fuller testing generally failed to find a unit root in most subjects. Given this result, the first difference was taken for each dataset to give ΔABP, ΔICP, and ΔrSO_2_. Augmented Dickie Fuller and Kwiatkowski–Phillips–Schmidt–Shin testing now indicated stationarity in virtually all data streams in all subjects and no unit roots. The results of the Augmented Dickie Fuller and Kwiatkowski–Phillips–Schmidt–Shin testing, both pre- and post-differencing, can be found in Additional file 2.

**Fig. 3:**
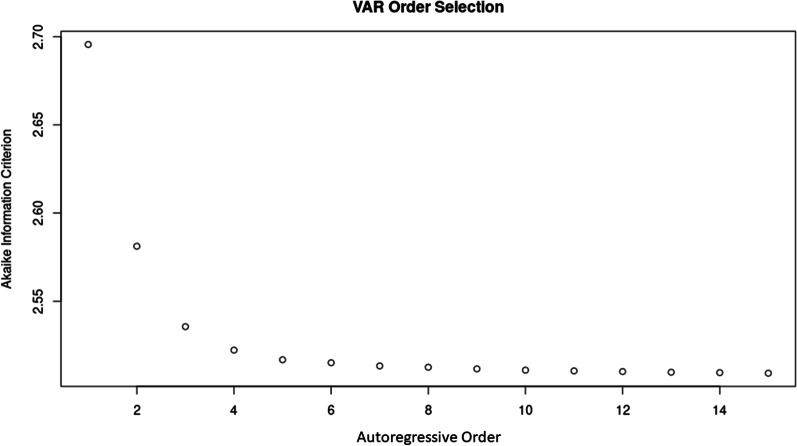
A plot of Akaike Information Criterion versus autoregressive order of the tri-variate vector autoregressive (VAR) model. There is limited improvement in Akaike Information Criterion beyond an order of 5

The plot of Akaike Information Criterion values versus vector autoregressive model order can be seen in Fig. 3**.** As can be seen in this plot, the marginal improvements in Akaike Information Criterion with increasing order greatly declined after order of 5. This indicated that models of order above 5 provided little improvement in accuracy but significantly increased computational complexity. As such, a tri-variate (ΔABP, ΔICP, and ΔrSO_2_) Vector Autoregressive model of order of 5 was constructed and used to generate the impulse response function plots.

**Fig. 4:**
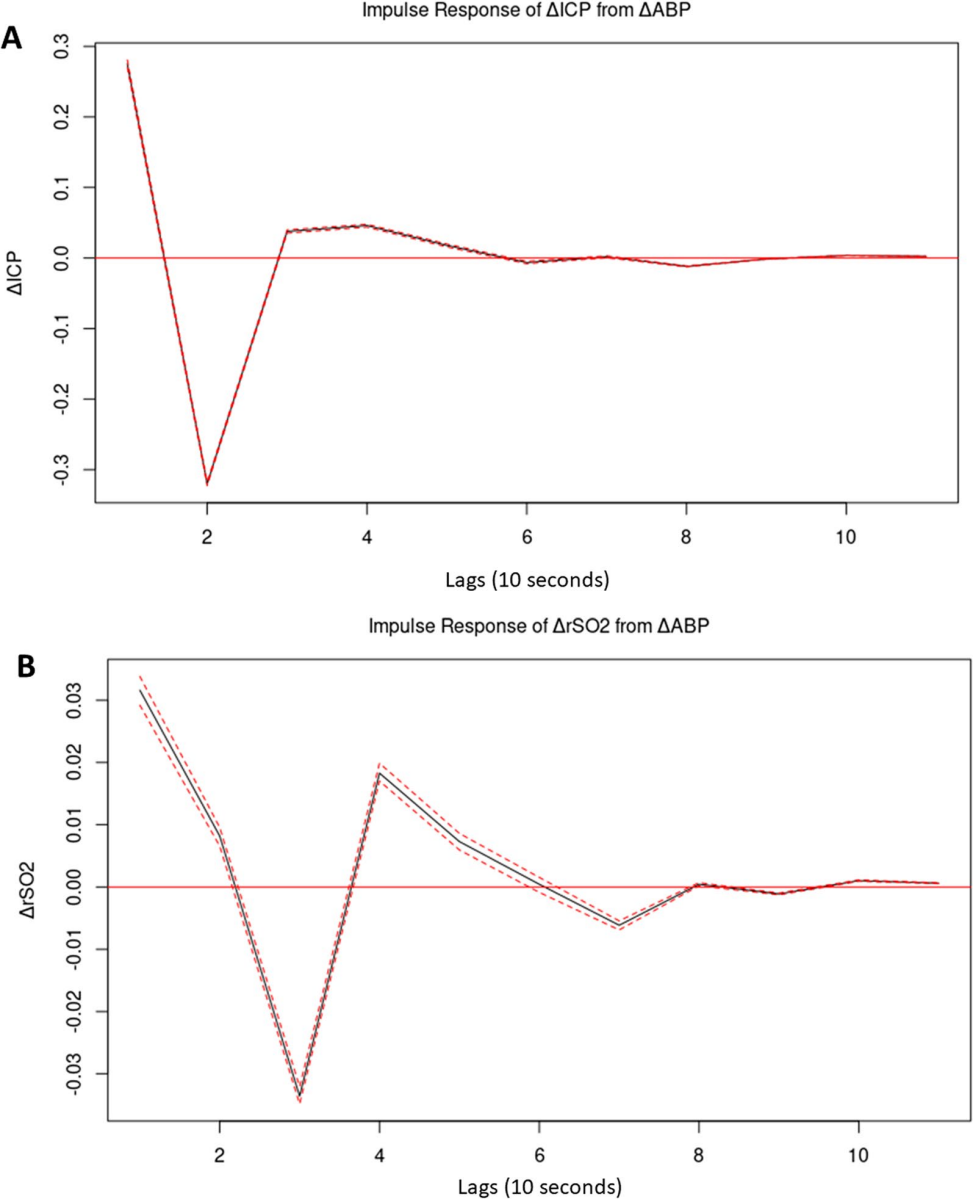
**A** Shows the modeled resulting response in change in intracranial pressure (ΔICP) to an orthogonal impulse in change in arterial blood pressure (ΔABP). **B** Shows the modeled resulting response in change in regional cerebral oxygen saturation (ΔrSO_2_) to an orthogonal impulse in change in arterial blood pressure (ΔABP). The 95% confidence intervals are indicated by the red dashed line. Note that in both plots there is an initial rise followed by subsequent negative and positive overcorrections and then a return to steady state between Lag 6 and 8

Figure 4a shows the impulse response function plot of the response in ΔICP to an orthogonal impulse in ΔABP. It can be seen that the impulse in ΔABP causes an initial sharp increase in ΔICP followed by a large negative overcorrection in ΔICP by lag 2. The ΔICP then, again, positively overcorrects around a lag of 3 and 4. ΔICP then oscillates around 0 and, by a lag of 6, is back at steady state. Figure 4b shows the impulse response function plot of the response of ΔrSO_2_ to an impulse in ΔABP. As with ΔICP, an impulse in ΔABP causes an initial sharp increase in ΔrSO_2_ followed by a large negative overcorrection in ΔrSO_2_, this time by lag 3. There is again a positive overcorrection at lag 4 with ΔrSO_2_ values subsequently oscillating around 0 until a return to steady state at around a lag of 8.

The impulse response function plots for individual subjects can be seen in Additional file 3. Of the 83 subjects, the generated response of ΔICP and ΔrSO_2_ to an impulse of ΔABP was of similar form to one another in 63 of the subjects. This indicated that, even on the individual subject level, ΔrSO_2_ and ΔICP responded similarly to an impulse in ΔABP. Of note, the variability between subjects in responses to an impulse of ΔABP was greater for ΔrSO_2_ than for ΔICP. Additionally, for each subject, the response of ΔICP to an impulse in ΔABP was similar to the impulse response function plot for the overall cohort.

### Granger causality testing

The results of the Granger causality testing for each subject can be seen in Additional file 4. In 67 subjects, by comparing the magnitude of the F-statistic, the Granger causal relationship of ΔABP → ΔrSO_2_ was greater than ΔrSO_2_ → ΔABP. Similarly, in 76 subjects, by comparing the magnitude of the F-statistic, the Granger causal relationship of ΔABP → ΔICP was greater than ΔICP → ΔABP. This indicates that the directionality of temporal causation is more in favor of ΔABP → ΔrSO_2_ or ΔICP**.** Finally, for most patients, the F-statistics for ΔABP → ΔICP were generally greater than the F-statistic for ΔABP → ΔrSO_2_ which may indicate a stronger relationship between ΔABP and ΔICP than ΔABP and ΔrSO_2_.

## Discussion

In this relatively large retrospective cohort study of extremely unique prospectively collected high-resolution physiologic data in critically ill TBI patients, the relationship between ICP- and rSO_2_-based indices of cerebrovascular reactivity was explored using classical machine learning and multivariate time-series analysis. Several interesting insights can be drawn from the analysis of this exceedingly rare dataset. Through agglomerative hierarchical clustering and principal component analysis, it is clear that ICP-based parameters are closely associated with one another. Interestingly, through the agglomerative hierarchical clustering, we can see that the ICP-based indices are also more closely related to rSO_2_-based indices of cerebrovascular reactivity than other physiological parameters.

A significant finding of this study was that COx and COx_a are closely associated with one another in both principal component analysis and agglomerative hierarchical clustering. Using ABP instead of CPP as the surrogate for driving pressure may be an adequate substitution to reduce the invasiveness of cerebrovascular reactivity monitoring as non-invasive continuous ABP monitoring is already possible. This opens the door to entirely non-invasive cerebrovascular reactivity monitoring [13, 14]. This is also supported by the close association between PAx and RAC, where ABP and CPP are used as the surrogate for driving pressure, respectively, in both the agglomerative hierarchical clustering dendrogram and principal component analysis biplot. Additionally, we see that CPP is much more closely related to ABP than ICP in both analyses. For the purposes of monitoring cerebrovascular reactivity ABP and CPP may then have similar value as surrogates for driving pressure, with ABP being the clearly less invasive option.

ICP- and rSO_2_-based indices of cerebrovascular reactivity are closely associated, as identified through agglomerative hierarchical clustering and principal component analysis analysis, with co-clustering on agglomerative hierarchical clustering and close proximity noted on the principal component analysis biplot. Of note, on the principal component analysis biplot, while ICP-based indices are nearly coaxial with PC2, rSO_2_-based indices are nearly equally composed of PC1 and PC2 and seem to have a stronger relationship with ABP and CPP than ICP-based indices do.

When examining the other physiologic parameters, some interesting conclusions can be made. As previously mentioned, CPP is much more closely associated with ABP than with ICP, which may indicate that in the setting of critically ill TBI patients managed in the ICU, minute-to-minute CPP is much more strongly tied to ABP than to ICP. This is not entirely surprising as ICP is a very tightly controlled parameter, especially in the stable, but critically ill, TBI patient. Also of interest, through agglomerative hierarchical clustering and principal component analysis we see that absolute ICP and rSO_2_ are not well associated even though their respective indices of cerebrovascular reactivity are.

Through vector autoregressive modeling and impulse response function plots, over the cohort and in individual subjects, ΔICP and ΔrSO_2_ do typically share a similar response to a sudden change in ΔABP. Their similarity in response is found over the temporal resolution used in the calculation of their respective indices of cerebrovascular reactivity, PRx and COx_a, as these indices initially decimate these physiologic signals using a 10-s moving average filter [5]. Additionally, since Pearson correlation coefficients are used in the derivation of both ICP- and rSO_2_-based indices, it is the change in these parameters in response to change in ABP that is of primary interest; which is what has been examined in the vector autoregressive impulse response function analysis. The similarity in impulse response function plots indicate that both ICP and rSO_2_ may be adequate surrogates for pulsatile CBV in the calculation of cerebrovascular indices despite sharing little in common as standalone parameters. This analysis helps reconcile the findings of previous studies that failed to find value in NIRS-based parameters in the monitoring of critically ill TBI patients [38–42] with studies that have found NIRS-based indices of cerebrovascular reactivity to be similar to ICP-based indices [15–17]. If change in ICP and change in rSO_2_ are related but not their absolute values, it can be understood why ICP-based and rSO_2_-based indices of cerebrovascular reactivity associate with one another even when ICP and rSO_2_ are not in agglomerative hierarchical clustering and principal component analysis.

Further examination of the cohort impulse response function plots also gives insight into the basic temporal mechanisms of cerebrovascular reactivity. We see that an initial impulse in ΔABP is initially met with a positive ΔICP and ΔrSO_2_ which would be consistent with an initial passive arteriolar dilation. This is similar to what has been observed in transcranial Doppler-based transient hyperemic response testing of dynamic cerebral autoregulation [43, 44]. This is then followed by a negative phase in both ΔICP and ΔrSO_2_ which could represent a period of active vasoconstriction. The third positive phase in ΔICP and ΔrSO_2_ may indicate that generally there is a slight overcorrection in second phase vasoconstriction that requires an additional vasodilatory phase to reach a subsequent steady state. This is an interesting finding that will guide future exploration into the fundamental basis of cerebrovascular reactivity.

Finally, through the Granger causality testing in individual subjects, generally changes in ABP temporally precede and are associated with changes in both rSO_2_ and ICP. This supports the use of ABP as a surrogate for driving pressure and both ICP or rSO_2_ as a surrogate for pulsatile CBV in indices of cerebrovascular reactivity.

### Limitations

While this study leverages a unique dataset and advanced data science techniques, there are notable limitations to this study. First, the cohort is from a single institution, and as a result, several regional factors, such as local management norms, may limit the generalizability of these findings at institutions where patient populations and management differ. Additionally, the decision was made to aggregate for the agglomerative hierarchical clustering and principal component analysis. This approach leads to the loss of information as individual patient factors are not examined and the hierarchical nature of the dataset is ignored. Due to the novel nature of this cohort and the exploratory nature of this study, it was felt that this was appropriate. However, individual subject data were examined through the vector autoregressive modeling and impulse response plots as well as with the Granger causality testing. Secondly, the retrospective nature of this study means that independent variables, such as ABP, were not intentionally manipulated to evaluate changes in dependent variables, such as ICP and rSO_2_. While impulse response function plots help model the response of one parameter to another, they are not a substitution to interventional studies where the response of ICP and rSO_2_ to changes in ABP can be induced and observed prospectively. Finally, as rSO_2_ is influenced by blood oxygen content, the lack of high temporal resolution oxygenation parameters, such as oxygen saturation (SpO_2_) and partial pressure of oxygen in arterial blood (PaO_2_), means that their influence is not directly accounted for in this analysis.

### Future work

This study lays the groundwork for future examination of the viability of rSO_2_-based indices of cerebrovascular reactivity. Prior to their wider adoption as both a research and clinical tool in the setting of TBI, further work is needed. Prospective interventional studies in large animal models with concurrent ICP and rSO_2_ monitoring may help further validate rSO_2_ as an adequate measure of pulsatile CBV. Additionally, studies aimed at modeling ICP-based indices of cerebrovascular reactivity from rSO_2_-based indices may further strengthen confidence in its use as a less invasive alternative. Similar work has been conducted using non-invasive transcranial Doppler cerebrovascular reactivity indices [45, 46]. Beyond this, studies examining the prognostic utility of rSO_2_ and its derived indices in a large multi-institutional cohort of critically ill TBI patients are also vital. Finally, leveraging the non-invasive nature of NIRS, studies examining the trajectory of cerebrovascular reactivity through the chronic phase of injury will help better understand the wider picture of dysfunctional cerebrovascular reactivity following TBI, and lead to improved prognostication as well as therapeutic targets.

This study also presented some insights into the basic temporal mechanisms of cerebrovascular reactivity. If rSO_2_-based indices of cerebrovascular reactivity are found to be similar to ICP-based indices, studies in a healthy control population may help identify what vascular reactivity patterns are attributable to either the functional or dysfunctional state.

## Conclusion

In this retrospective cohort study of unique prospectively collected high-resolution physiologic data in critically ill TBI patients, ICP- and rSO_2_-based indices were found to be distinct but related metrics. Within these indices, both ABP and CPP were found to have similar utility as surrogates for driving pressure. Further, ΔrSO_2_ and ΔICP were found to respond similarly to modeled impulses in ΔABP, indicating that rSO_2_ might also be an adequate measure of pulsatile CBV in the determination of continuous cerebrovascular reactivity indices. This opens the door to further research that can validate the prognostic utility of rSO_2_-based indices in TBI and leverage its non-invasive nature to further the understanding of dysfunctional cerebrovascular reactivity following TBI.

### Supplementary Information

**Additional file 1:** A cumulative Scree plot indicating the summative variance explained with each additional principal component.

**Additional file 2:** Summary table of the KPSS and ADF statistic values for each subject, before and after taking the first difference.

**Additional file 3:** The individual subject order 5 Vector Autoregressive model Impulse Response change in intracranial pressure (ΔICP) to an orthogonal impulse in change in arterial blood pressure (ΔABP) and change in regional cerebral oxygen saturation (ΔrSO_2_) to an orthogonal impulse in change in ΔABP and the number of 10-second datapoints for each subject in the study used in constructing the Vector Autoregressive model Impulse Response plots.

**Additional file 4:** The results of Granger causality testing for individual subjects.

## Data Availability

The datasets used and/or analyzed during the current study are available from the corresponding author on reasonable request.

## Abbreviations

ABP: Arterial blood pressure
AMP: Pulse amplitude of ICP
BLAS: Basic linear algebra subprograms
CBF: Cerebral blood flow
CBV: Cerebral blood volume
COx: Cerebral Oxygenation Index with CPP
COx_a: Cerebral Oxygenation Index with ABP
CPP: Cerebral perfusion pressure
.csv: Comma separated vale
ΔABP: Change in arterial blood pressure
ΔICP: Change in intracranial pressure
ΔrSO_2_: Change in regional cerebral oxygen saturation
GCS: Glasgow Coma Scale
ICP: Intracranial pressure
ICU: Intensive care unit
LAPACK: Linear algebra package
NIRS: Near infrared spectroscopy
PAx: Pulse Amplitude Index
PaO_2_: Partial pressure of oxygen in arterial blood
PC1: Principal component 1
PC2: Principal component 2
PRx: Pressure Reactivity Index
RAC: Correlation (R) between pulse amplitude of ICP (A) and cerebral perfusion pressure (C)
rSO_2_: Regional cerebral oxygen saturation
SpO_2_: Oxygen saturation
TBI: Traumatic brain injury
