# Supplementary material for: Statistical properties of cerebral near infrared and intracranial pressure-based cerebrovascular reactivity metrics in moderate and severe neural injury: a machine learning and time-series analysis
Source: Intensive Care Med Exp. 2023 Aug 28;11:57. doi: 10.1186/s40635-023-00541-3 (PMC10460757; doi:10.1186/s40635-023-00541-3)
Supplement: Supplementary file 1 — Additional file 1: A cumulative Scree plot indicating the summative variance explained with each additional principal component. [file 40635_2023_541_MOESM1_ESM.docx]

**Additional File 1**


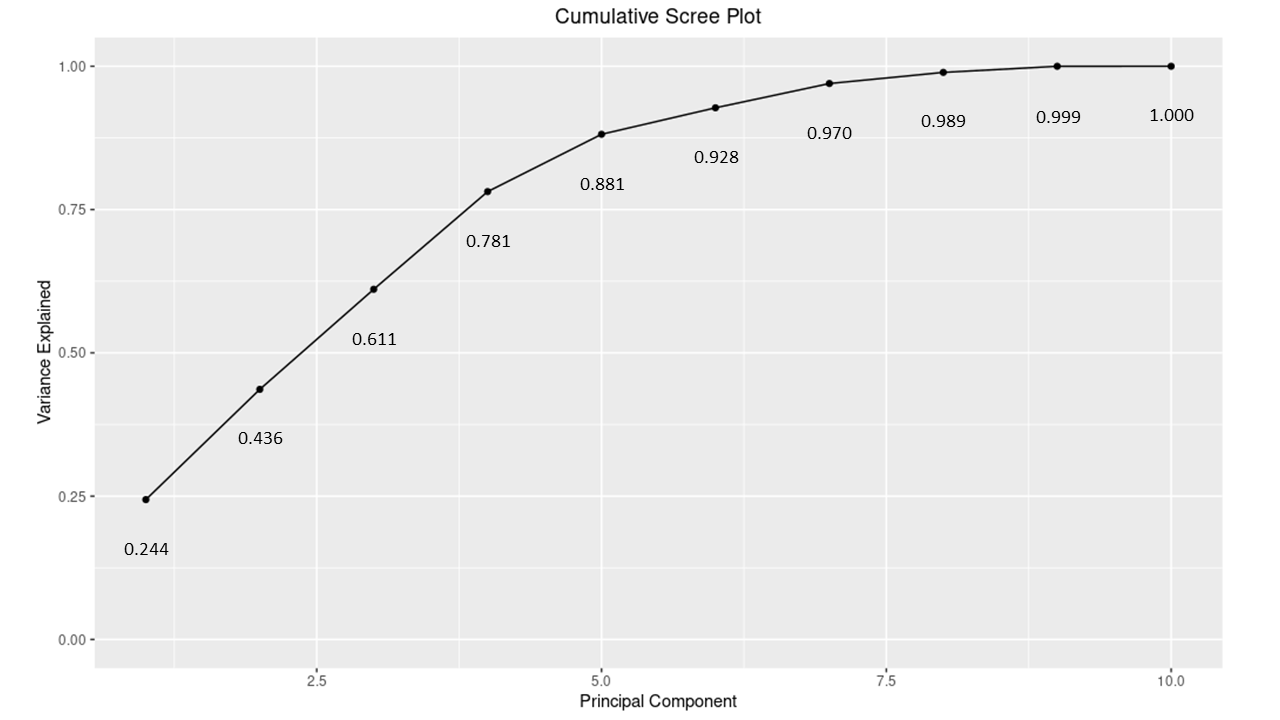
 ***Figure S1:*** *A cumulative Scree plot indicating the summative variance explained with each additional principal component. The proportion of variance explained is listed under each point. As can be seen in the plot, nearly 50% of the variance in the data is explained through the first two principal components.*
