# Supplementary material for: Statistical properties of cerebral near infrared and intracranial pressure-based cerebrovascular reactivity metrics in moderate and severe neural injury: a machine learning and time-series analysis
Source: Intensive Care Med Exp. 2023 Aug 28;11:57. doi: 10.1186/s40635-023-00541-3 (PMC10460757; doi:10.1186/s40635-023-00541-3)
Supplement: Supplementary file 2 — Additional file 2: Summary table of the KPSS and ADF statistic values for each subject, before and after taking the first difference. [file 40635_2023_541_MOESM2_ESM.docx]

**Additional File 2**

***Table S1:*** *Summary table of the KPSS and ADF statistic values for each subject, before and after taking the first difference.*

| **Subject ID** | **KPSS Statistic for ABP** | **ADF Statistic for ABP** | **KPSS Statistic for ICP** | **ADF Statistic for ICP** | **KPSS Statistic for rSO_2_** | **ADF Statistic for rSO_2_** | **KPSS Statistic for ΔABP** | **ADF Statistic for ΔABP** | **KPSS Statistic for ΔICP** | **ADF Statistic for ΔICP** | **KPSS Statistic for Δ rSO_2_** | **ADF Statistic for ΔrSO_2_** |
| --- | --- | --- | --- | --- | --- | --- | --- | --- | --- | --- | --- | --- |
| 1 | <0.01 | <0.01 | <0.01 | <0.01 | <0.01 | <0.01 | >0.1 | <0.01 | >0.1 | <0.01 | >0.1 | <0.01 |
| 2 | <0.01 | <0.01 | <0.01 | <0.01 | <0.01 | <0.01 | >0.1 | <0.01 | >0.1 | <0.01 | >0.1 | <0.01 |
| 3 | <0.01 | <0.01 | <0.01 | <0.01 | <0.01 | 0.51 | >0.1 | <0.01 | >0.1 | <0.01 | >0.1 | <0.01 |
| 4 | <0.01 | <0.01 | <0.01 | <0.01 | <0.01 | 0.02 | >0.1 | <0.01 | >0.1 | <0.01 | >0.1 | <0.01 |
| 5 | <0.01 | <0.01 | <0.01 | <0.01 | <0.01 | <0.01 | >0.1 | <0.01 | >0.1 | <0.01 | >0.1 | <0.01 |
| 6 | <0.01 | <0.01 | <0.01 | <0.01 | <0.01 | 0.08 | >0.1 | <0.01 | >0.1 | <0.01 | >0.1 | <0.01 |
| 7 | <0.01 | <0.01 | <0.01 | <0.01 | <0.01 | <0.01 | >0.1 | <0.01 | >0.1 | <0.01 | >0.1 | <0.01 |
| 8 | <0.01 | <0.01 | <0.01 | <0.01 | <0.01 | <0.01 | >0.1 | <0.01 | >0.1 | <0.01 | >0.1 | <0.01 |
| 9 | <0.01 | <0.01 | <0.01 | <0.01 | <0.01 | <0.01 | >0.1 | <0.01 | >0.1 | <0.01 | >0.1 | <0.01 |
| 10 | <0.01 | <0.01 | <0.01 | <0.01 | <0.01 | <0.01 | >0.1 | <0.01 | >0.1 | <0.01 | >0.1 | <0.01 |
| 11 | <0.01 | <0.01 | <0.01 | <0.01 | <0.01 | 0.37 | >0.1 | <0.01 | >0.1 | <0.01 | >0.1 | <0.01 |
| 12 | <0.01 | <0.01 | <0.01 | <0.01 | <0.01 | <0.01 | >0.1 | <0.01 | >0.1 | <0.01 | >0.1 | <0.01 |
| 13 | <0.01 | <0.01 | <0.01 | 0.47 | <0.01 | 0.66 | >0.1 | <0.01 | >0.1 | <0.01 | >0.1 | <0.01 |
| 14 | <0.01 | 0.02 | <0.01 | <0.01 | <0.01 | <0.01 | >0.1 | <0.01 | >0.1 | <0.01 | >0.1 | <0.01 |
| 15 | <0.01 | <0.01 | <0.01 | <0.01 | <0.01 | 0.02 | >0.1 | <0.01 | >0.1 | <0.01 | >0.1 | <0.01 |
| 16 | <0.01 | <0.01 | <0.01 | 0.23 | <0.01 | 0.06 | >0.1 | <0.01 | >0.1 | <0.01 | >0.1 | <0.01 |
| 17 | <0.01 | <0.01 | <0.01 | <0.01 | <0.01 | <0.01 | >0.1 | <0.01 | >0.1 | <0.01 | >0.1 | <0.01 |
| 18 | <0.01 | <0.01 | <0.01 | <0.01 | <0.01 | <0.01 | >0.1 | <0.01 | >0.1 | <0.01 | >0.1 | <0.01 |
| 19 | <0.01 | <0.01 | <0.01 | <0.01 | <0.01 | <0.01 | >0.1 | <0.01 | >0.1 | <0.01 | >0.1 | <0.01 |
| 20 | <0.01 | <0.01 | <0.01 | <0.01 | <0.01 | <0.01 | >0.1 | <0.01 | >0.1 | <0.01 | >0.1 | <0.01 |
| 21 | <0.01 | <0.01 | <0.01 | <0.01 | <0.01 | <0.01 | >0.1 | <0.01 | >0.1 | <0.01 | >0.1 | <0.01 |
| 22 | <0.01 | <0.01 | <0.01 | <0.01 | <0.01 | <0.01 | >0.1 | <0.01 | >0.1 | <0.01 | >0.1 | <0.01 |
| 23 | <0.01 | <0.01 | <0.01 | <0.01 | <0.01 | <0.01 | >0.1 | <0.01 | >0.1 | <0.01 | >0.1 | <0.01 |
| 24 | <0.01 | <0.01 | <0.01 | <0.01 | <0.01 | <0.01 | >0.1 | <0.01 | >0.1 | <0.01 | >0.1 | <0.01 |
| 25 | <0.01 | <0.01 | <0.01 | <0.01 | <0.01 | 0.09 | >0.1 | <0.01 | >0.1 | <0.01 | >0.1 | <0.01 |
| 26 | <0.01 | <0.01 | <0.01 | <0.01 | <0.01 | 0.26 | >0.1 | <0.01 | >0.1 | <0.01 | >0.1 | <0.01 |
| 27 | <0.01 | <0.01 | <0.01 | <0.01 | <0.01 | 0.46 | >0.1 | <0.01 | >0.1 | <0.01 | >0.1 | <0.01 |
| 28 | <0.01 | <0.01 | <0.01 | <0.01 | <0.01 | 0.04 | >0.1 | <0.01 | >0.1 | <0.01 | >0.1 | <0.01 |
| 29 | <0.01 | <0.01 | <0.01 | <0.01 | <0.01 | <0.01 | >0.1 | <0.01 | >0.1 | <0.01 | >0.1 | <0.01 |
| 30 | <0.01 | <0.01 | <0.01 | <0.01 | <0.01 | <0.01 | >0.1 | <0.01 | >0.1 | <0.01 | >0.1 | <0.01 |
| 31 | <0.01 | <0.01 | <0.01 | 0.57 | <0.01 | <0.01 | >0.1 | <0.01 | >0.1 | <0.01 | >0.1 | <0.01 |
| 32 | <0.01 | <0.01 | <0.01 | <0.01 | <0.01 | 0.2 | >0.1 | <0.01 | >0.1 | <0.01 | >0.1 | <0.01 |
| 33 | <0.01 | <0.01 | <0.01 | <0.01 | <0.01 | <0.01 | >0.1 | <0.01 | >0.1 | <0.01 | >0.1 | <0.01 |
| 34 | <0.01 | <0.01 | <0.01 | <0.01 | <0.01 | <0.01 | >0.1 | <0.01 | >0.1 | <0.01 | >0.1 | <0.01 |
| 35 | <0.01 | <0.01 | <0.01 | <0.01 | <0.01 | 0.06 | >0.1 | <0.01 | >0.1 | <0.01 | >0.1 | <0.01 |
| 36 | <0.01 | 0.29 | <0.01 | <0.01 | <0.01 | 0.92 | >0.1 | <0.01 | >0.1 | <0.01 | >0.1 | <0.01 |
| 37 | <0.01 | <0.01 | <0.01 | <0.01 | <0.01 | <0.01 | >0.1 | <0.01 | >0.1 | <0.01 | >0.1 | <0.01 |
| 38 | <0.01 | <0.01 | <0.01 | <0.01 | <0.01 | <0.01 | >0.1 | <0.01 | >0.1 | <0.01 | >0.1 | <0.01 |
| 39 | <0.01 | <0.01 | <0.01 | 0.12 | <0.01 | 0.02 | >0.1 | <0.01 | >0.1 | <0.01 | >0.1 | <0.01 |
| 40 | <0.01 | <0.01 | <0.01 | <0.01 | <0.01 | 0.02 | >0.1 | <0.01 | >0.1 | <0.01 | >0.1 | <0.01 |
| 41 | <0.01 | <0.01 | <0.01 | <0.01 | <0.01 | 0.08 | >0.1 | <0.01 | >0.1 | <0.01 | >0.1 | <0.01 |
| 42 | <0.01 | <0.01 | <0.01 | 0.02 | <0.01 | 0.02 | >0.1 | <0.01 | >0.1 | <0.01 | >0.1 | <0.01 |
| 43 | <0.01 | <0.01 | <0.01 | <0.01 | <0.01 | 0.23 | >0.1 | <0.01 | >0.1 | <0.01 | >0.1 | <0.01 |
| 44 | <0.01 | <0.01 | <0.01 | <0.01 | <0.01 | 0.54 | >0.1 | <0.01 | >0.1 | <0.01 | >0.1 | <0.01 |
| 45 | <0.01 | <0.01 | <0.01 | <0.01 | <0.01 | <0.01 | >0.1 | <0.01 | >0.1 | <0.01 | >0.1 | <0.01 |
| 46 | <0.01 | <0.01 | <0.01 | <0.01 | <0.01 | 0.02 | >0.1 | <0.01 | >0.1 | <0.01 | >0.1 | <0.01 |
| 47 | <0.01 | <0.01 | <0.01 | <0.01 | <0.01 | <0.01 | >0.1 | <0.01 | >0.1 | <0.01 | >0.1 | <0.01 |
| 48 | <0.01 | <0.01 | <0.01 | <0.01 | <0.01 | <0.01 | >0.1 | <0.01 | >0.1 | <0.01 | >0.1 | <0.01 |
| 49 | <0.01 | <0.01 | <0.01 | 0.23 | <0.01 | 0.03 | >0.1 | <0.01 | >0.1 | <0.01 | >0.1 | <0.01 |
| 50 | <0.01 | <0.01 | <0.01 | <0.01 | <0.01 | <0.01 | >0.1 | <0.01 | >0.1 | <0.01 | >0.1 | <0.01 |
| 51 | <0.01 | <0.01 | <0.01 | 0.18 | <0.01 | <0.01 | >0.1 | <0.01 | >0.1 | <0.01 | 0.02 | <0.01 |
| 52 | <0.01 | <0.01 | <0.01 | <0.01 | <0.01 | <0.01 | >0.1 | <0.01 | >0.1 | <0.01 | >0.1 | <0.01 |
| 53 | <0.01 | <0.01 | <0.01 | <0.01 | <0.01 | <0.01 | >0.1 | <0.01 | >0.1 | <0.01 | >0.1 | <0.01 |
| 54 | <0.01 | <0.01 | <0.01 | <0.01 | <0.01 | 0.69 | >0.1 | <0.01 | >0.1 | <0.01 | >0.1 | <0.01 |
| 55 | <0.01 | <0.01 | <0.01 | <0.01 | <0.01 | <0.01 | >0.1 | <0.01 | >0.1 | <0.01 | >0.1 | <0.01 |
| 56 | <0.01 | <0.01 | <0.01 | <0.01 | <0.01 | <0.01 | >0.1 | <0.01 | >0.1 | <0.01 | >0.1 | <0.01 |
| 57 | <0.01 | <0.01 | <0.01 | <0.01 | <0.01 | 0.03 | >0.1 | <0.01 | >0.1 | <0.01 | >0.1 | <0.01 |
| 58 | <0.01 | <0.01 | <0.01 | <0.01 | <0.01 | 0.26 | >0.1 | <0.01 | >0.1 | <0.01 | >0.1 | <0.01 |
| 59 | <0.01 | <0.01 | <0.01 | 0.3 | <0.01 | <0.01 | >0.1 | <0.01 | >0.1 | <0.01 | >0.1 | <0.01 |
| 60 | <0.01 | <0.01 | <0.01 | <0.01 | <0.01 | <0.01 | >0.1 | <0.01 | >0.1 | <0.01 | >0.1 | <0.01 |
| 61 | <0.01 | <0.01 | <0.01 | 0.03 | <0.01 | 0.01 | >0.1 | <0.01 | >0.1 | <0.01 | >0.1 | <0.01 |
| 62 | <0.01 | <0.01 | <0.01 | <0.01 | <0.01 | <0.01 | >0.1 | <0.01 | >0.1 | <0.01 | >0.1 | <0.01 |
| 63 | <0.01 | <0.01 | <0.01 | <0.01 | <0.01 | <0.01 | >0.1 | <0.01 | >0.1 | <0.01 | >0.1 | <0.01 |
| 64 | <0.01 | <0.01 | <0.01 | <0.01 | <0.01 | <0.01 | >0.1 | <0.01 | >0.1 | <0.01 | >0.1 | <0.01 |
| 65 | <0.01 | <0.01 | <0.01 | <0.01 | <0.01 | <0.01 | >0.1 | <0.01 | >0.1 | <0.01 | >0.1 | <0.01 |
| 66 | <0.01 | <0.01 | <0.01 | <0.01 | <0.01 | <0.01 | >0.1 | <0.01 | >0.1 | <0.01 | >0.1 | <0.01 |
| 67 | <0.01 | <0.01 | <0.01 | <0.01 | <0.01 | 0.6 | >0.1 | <0.01 | >0.1 | <0.01 | >0.1 | <0.01 |
| 68 | <0.01 | <0.01 | <0.01 | <0.01 | <0.01 | <0.01 | >0.1 | <0.01 | >0.1 | <0.01 | >0.1 | <0.01 |
| 69 | <0.01 | <0.01 | <0.01 | <0.01 | <0.01 | <0.01 | >0.1 | <0.01 | >0.1 | <0.01 | >0.1 | <0.01 |
| 70 | <0.01 | <0.01 | <0.01 | <0.01 | <0.01 | <0.01 | >0.1 | <0.01 | >0.1 | <0.01 | >0.1 | <0.01 |
| 71 | <0.01 | <0.01 | <0.01 | <0.01 | <0.01 | <0.01 | >0.1 | <0.01 | >0.1 | <0.01 | >0.1 | <0.01 |
| 72 | <0.01 | <0.01 | <0.01 | <0.01 | <0.01 | 0.31 | >0.1 | <0.01 | >0.1 | <0.01 | >0.1 | <0.01 |
| 73 | <0.01 | <0.01 | <0.01 | 0.15 | <0.01 | 0.04 | >0.1 | <0.01 | >0.1 | <0.01 | >0.1 | <0.01 |
| 74 | <0.01 | <0.01 | <0.01 | <0.01 | <0.01 | 0.12 | >0.1 | <0.01 | >0.1 | <0.01 | >0.1 | <0.01 |
| 75 | <0.01 | <0.01 | <0.01 | <0.01 | <0.01 | 0.02 | >0.1 | <0.01 | >0.1 | <0.01 | >0.1 | <0.01 |
| 76 | <0.01 | <0.01 | <0.01 | <0.01 | <0.01 | <0.01 | >0.1 | <0.01 | >0.1 | <0.01 | >0.1 | <0.01 |
| 77 | <0.01 | <0.01 | <0.01 | <0.01 | <0.01 | <0.01 | >0.1 | <0.01 | >0.1 | <0.01 | >0.1 | <0.01 |
| 78 | <0.01 | 0.023 | <0.01 | 0.17 | <0.01 | 0.34 | >0.1 | <0.01 | >0.1 | <0.01 | >0.1 | <0.01 |
| 79 | <0.01 | <0.01 | <0.01 | <0.01 | <0.01 | <0.01 | >0.1 | <0.01 | >0.1 | <0.01 | >0.1 | <0.01 |
| 80 | <0.01 | <0.01 | <0.01 | <0.01 | <0.01 | <0.01 | >0.1 | <0.01 | >0.1 | <0.01 | >0.1 | <0.01 |
| 81 | <0.01 | <0.01 | <0.01 | <0.01 | <0.01 | <0.01 | >0.1 | <0.01 | >0.1 | <0.01 | >0.1 | <0.01 |
| 82 | <0.01 | <0.01 | <0.01 | <0.01 | <0.01 | <0.01 | >0.1 | <0.01 | >0.1 | <0.01 | >0.1 | <0.01 |
| 83 | <0.01 | <0.01 | <0.01 | <0.01 | <0.01 | <0.01 | >0.1 | <0.01 | >0.1 | <0.01 | >0.1 | <0.01 |

*ABP = Arterial Blood Pressure, ADF = Augmented Dickie Fuller, ICP = Intracranial Pressure, rSO_2_ = Regional Cerebral Oxygen Saturation, ΔABP = Change in Arterial Blood Pressure, ΔICP = Change in Intracranial Pressure, ΔrSO_2_ = Change in Regional Cerebral Oxygen Saturation, KPSS = Kwiatkowski–Phillips–Schmidt–Shin.*
