# Supplementary material for: Statistical properties of cerebral near infrared and intracranial pressure-based cerebrovascular reactivity metrics in moderate and severe neural injury: a machine learning and time-series analysis
Source: Intensive Care Med Exp. 2023 Aug 28;11:57. doi: 10.1186/s40635-023-00541-3 (PMC10460757; doi:10.1186/s40635-023-00541-3)
Supplement: Supplementary file 3 — Additional file 3: The individual subject order 5 Vector Autoregressive model Impulse Response change in intracranial pressure (ΔICP) to an orthogonal impulse in change in arterial blood pressure (ΔABP) and change in regional cerebral oxygen saturation (ΔrSO2) to an orthogonal impulse in change in ΔABP and the number of 10-second datapoints for each subject in the study used in constructing the Vector Autoregressive model Impulse Response plots. [file 40635_2023_541_MOESM3_ESM.docx]

**Additional File 3**

In this Additional file can be found the individual subject order 5 Vector Autoregressive model Impulse Response change in intracranial pressure (ΔICP) to an orthogonal impulse in change in arterial blood pressure (ΔABP) and change in regional cerebral oxygen saturation (ΔrSO2) to an orthogonal impulse in change in ΔABP. Additional, **Table S1** shows the number of 10-second datapoints for each subject in the study used in constructing the Vector Autoregressive model Impulse Response plots.


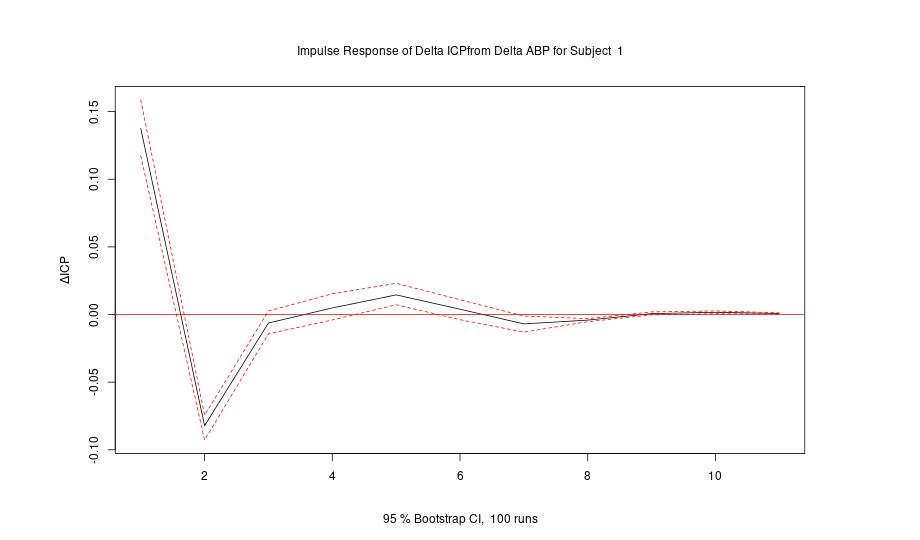


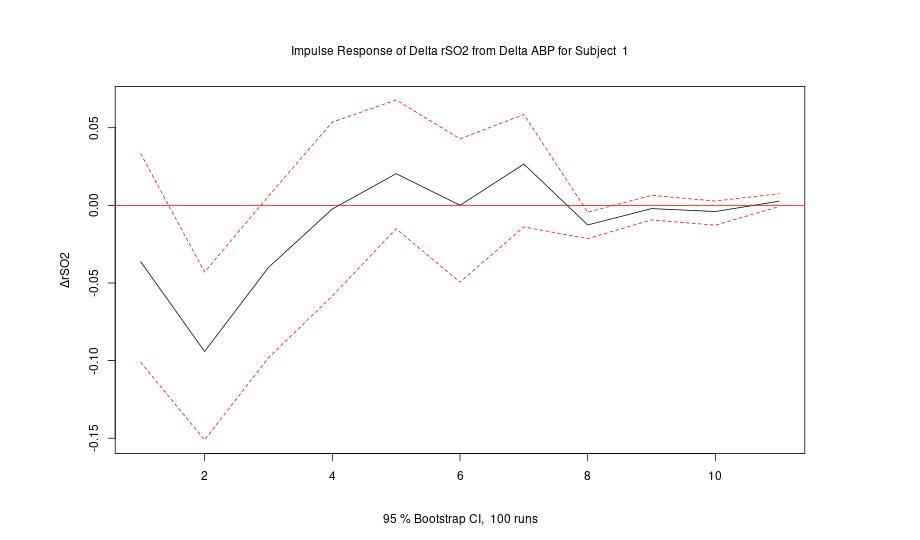


**Figure S1:** *Top panel shows the modeled resulting response in change in intracranial pressure (ΔICP) to an orthogonal impulse in change in arterial blood pressure (ΔABP). Bottom panel shows the modeled resulting response in change in regional cerebral oxygen saturation (ΔrSO_2_) to an orthogonal impulse in change in arterial blood pressure (ΔABP). The 95% confidence intervals are indicated by the red dashed line. Note the similar pattern of phases.*


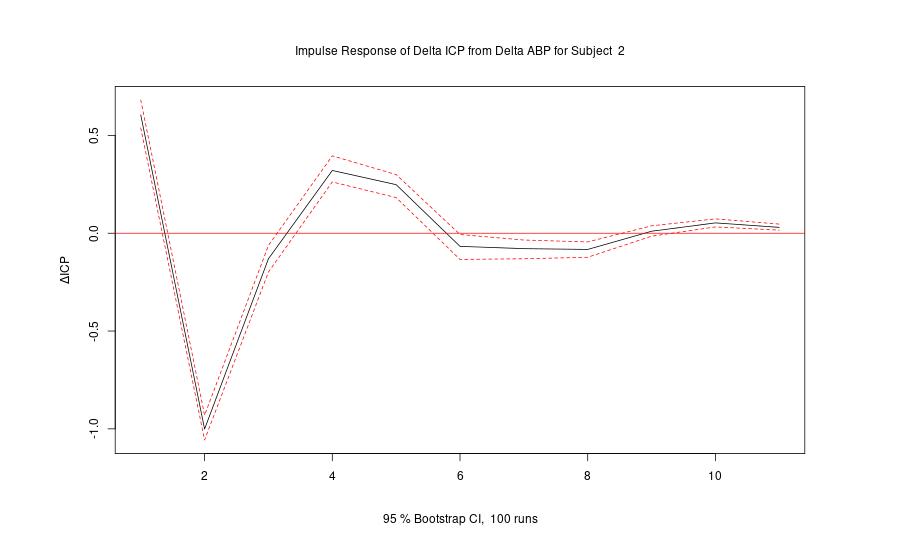

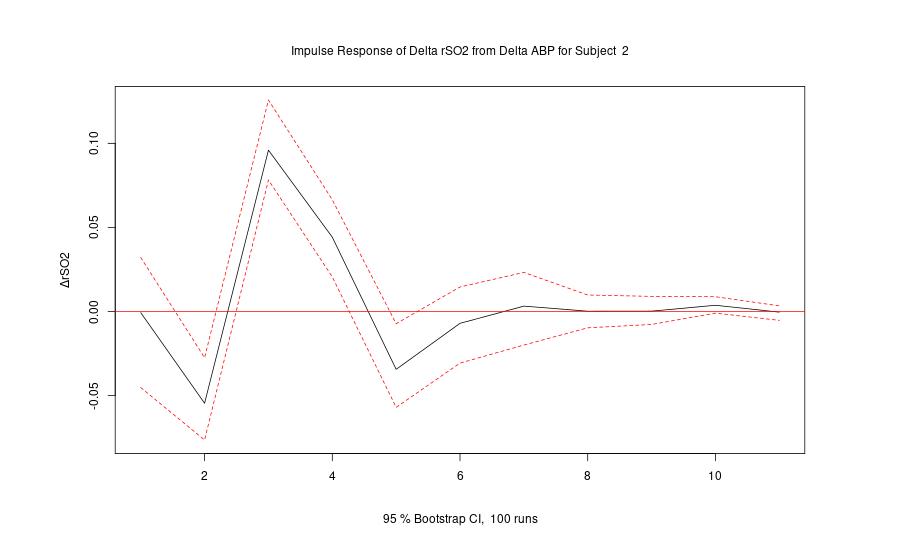
 **Figure S2:** *Top panel shows the modeled resulting response in change in intracranial pressure (ΔICP) to an orthogonal impulse in change in arterial blood pressure (ΔABP). Bottom panel shows the modeled resulting response in change in regional cerebral oxygen saturation (ΔrSO_2_) to an orthogonal impulse in change in arterial blood pressure (ΔABP). The 95% confidence intervals are indicated by the red dashed line. Note the similar pattern of phases.*


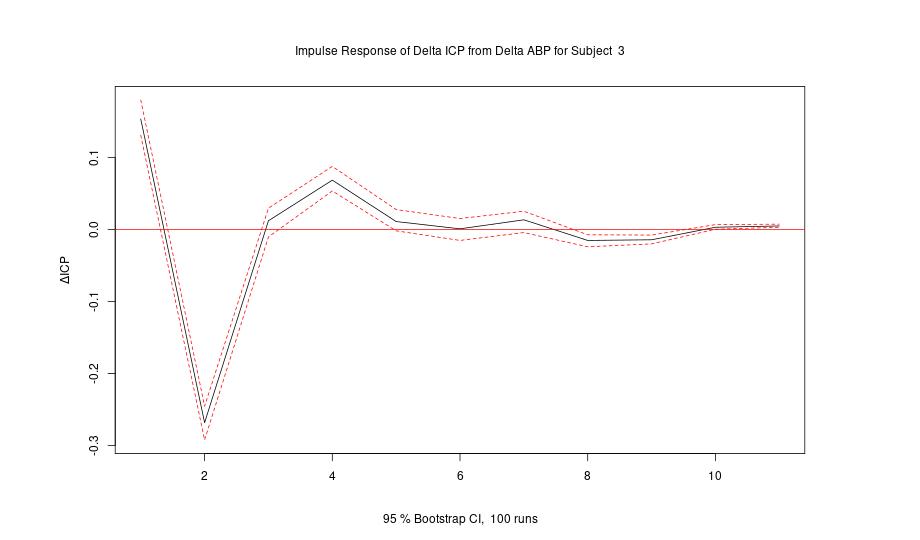

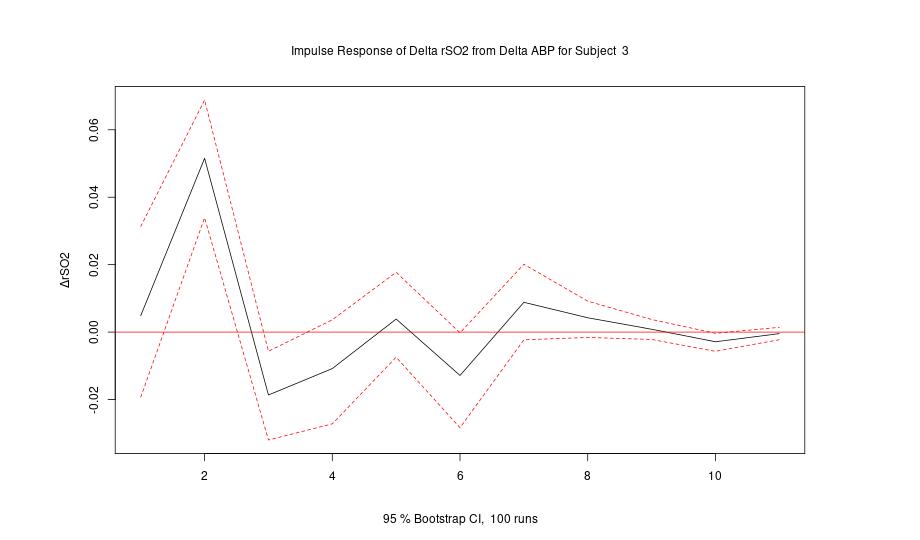


**Figure S3:** *Top panel shows the modeled resulting response in change in intracranial pressure (ΔICP) to an orthogonal impulse in change in arterial blood pressure (ΔABP). Bottom panel shows the modeled resulting response in change in regional cerebral oxygen saturation (ΔrSO_2_) to an orthogonal impulse in change in arterial blood pressure (ΔABP). The 95% confidence intervals are indicated by the red dashed line. Note the dissimilar pattern of phases.*


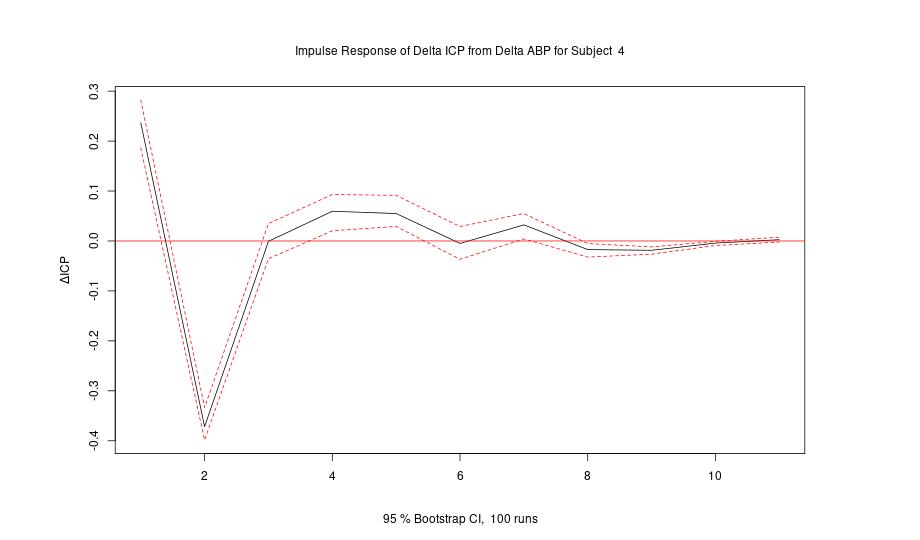

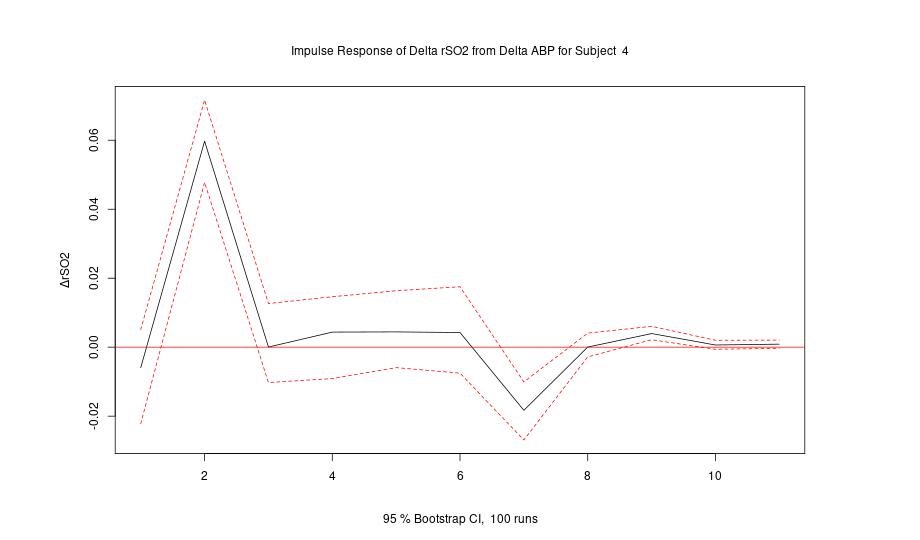


**Figure S4:** *Top panel shows the modeled resulting response in change in intracranial pressure (ΔICP) to an orthogonal impulse in change in arterial blood pressure (ΔABP). Bottom panel shows the modeled resulting response in change in regional cerebral oxygen saturation (ΔrSO_2_) to an orthogonal impulse in change in arterial blood pressure (ΔABP). The 95% confidence intervals are indicated by the red dashed line. Note the dissimilar pattern of phases.*


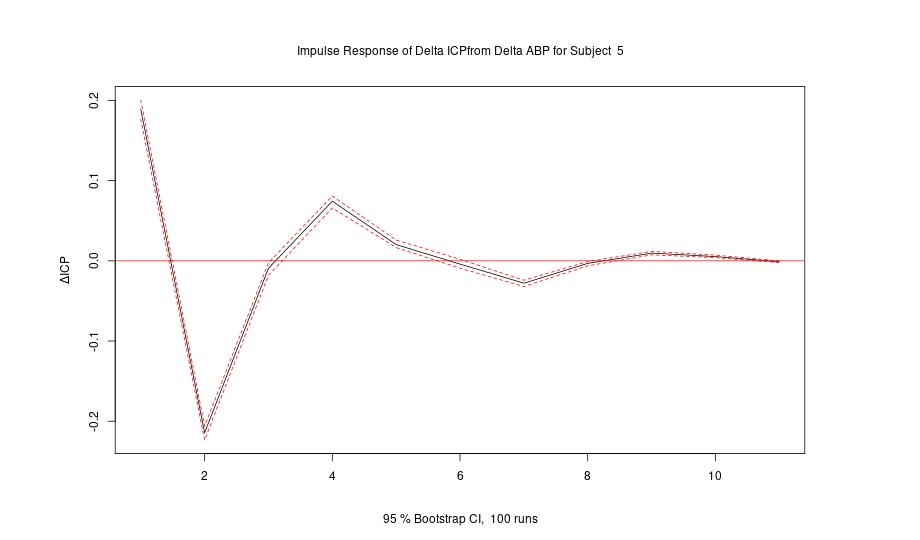

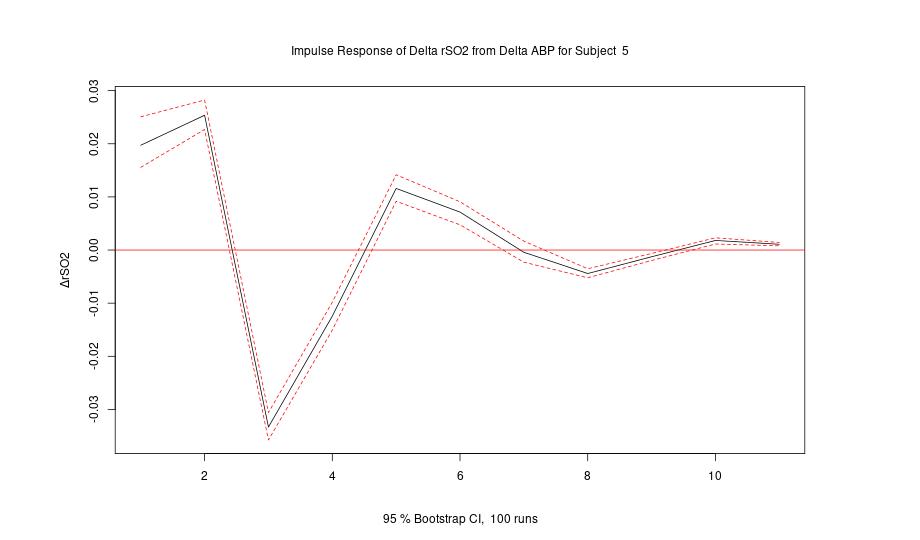


**Figure S5:** *Top panel shows the modeled resulting response in change in intracranial pressure (ΔICP) to an orthogonal impulse in change in arterial blood pressure (ΔABP). Bottom panel shows the modeled resulting response in change in regional cerebral oxygen saturation (ΔrSO_2_) to an orthogonal impulse in change in arterial blood pressure (ΔABP). The 95% confidence intervals are indicated by the red dashed line. Note the similar pattern of phases.*


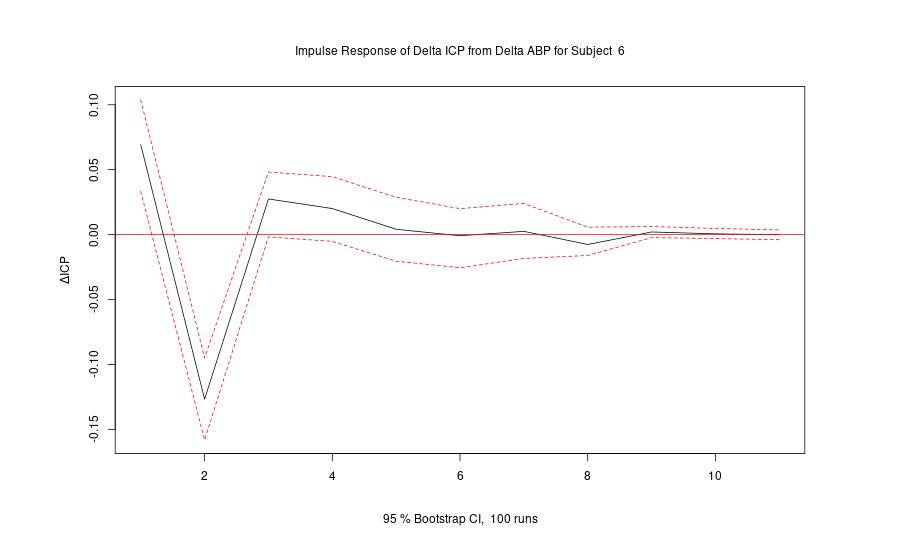


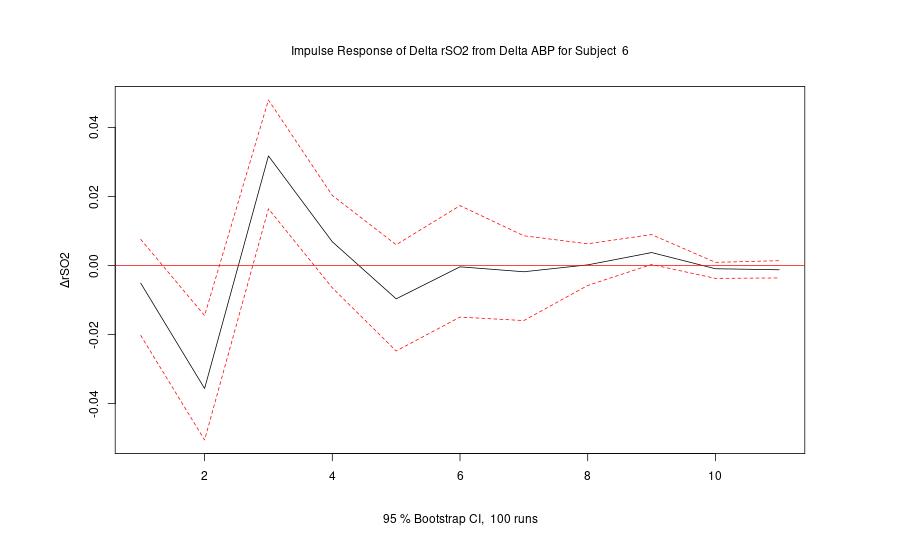


**Figure S6:** *Top panel shows the modeled resulting response in change in intracranial pressure (ΔICP) to an orthogonal impulse in change in arterial blood pressure (ΔABP). Bottom panel shows the modeled resulting response in change in regional cerebral oxygen saturation (ΔrSO_2_) to an orthogonal impulse in change in arterial blood pressure (ΔABP). The 95% confidence intervals are indicated by the red dashed line. Note the similar pattern of phases.*


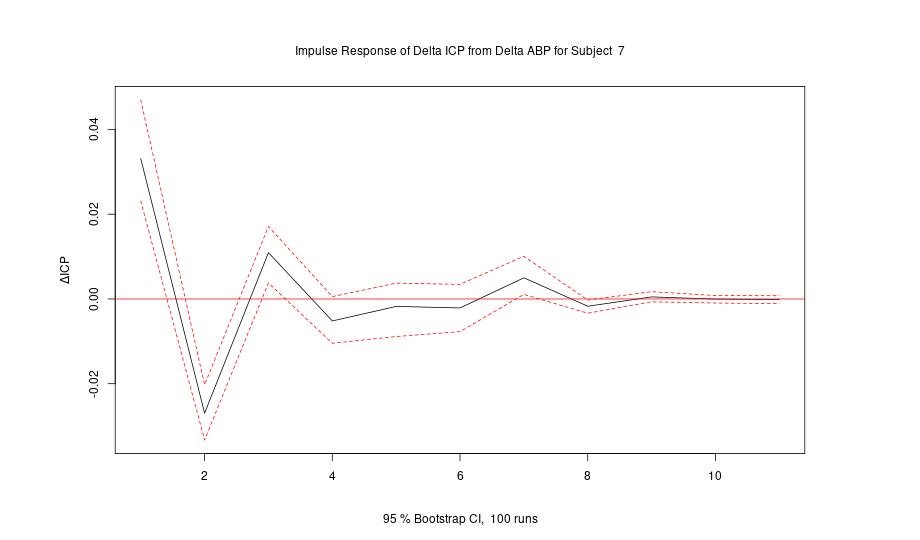

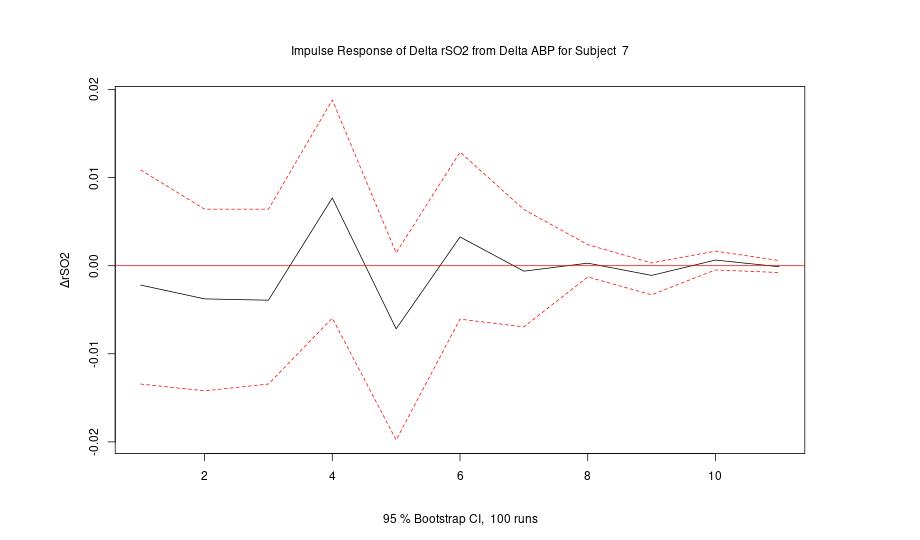


**Figure S7:** *Top panel shows the modeled resulting response in change in intracranial pressure (ΔICP) to an orthogonal impulse in change in arterial blood pressure (ΔABP). Bottom panel shows the modeled resulting response in change in regional cerebral oxygen saturation (ΔrSO_2_) to an orthogonal impulse in change in arterial blood pressure (ΔABP). The 95% confidence intervals are indicated by the red dashed line. Note the dissimilar pattern of phases.*


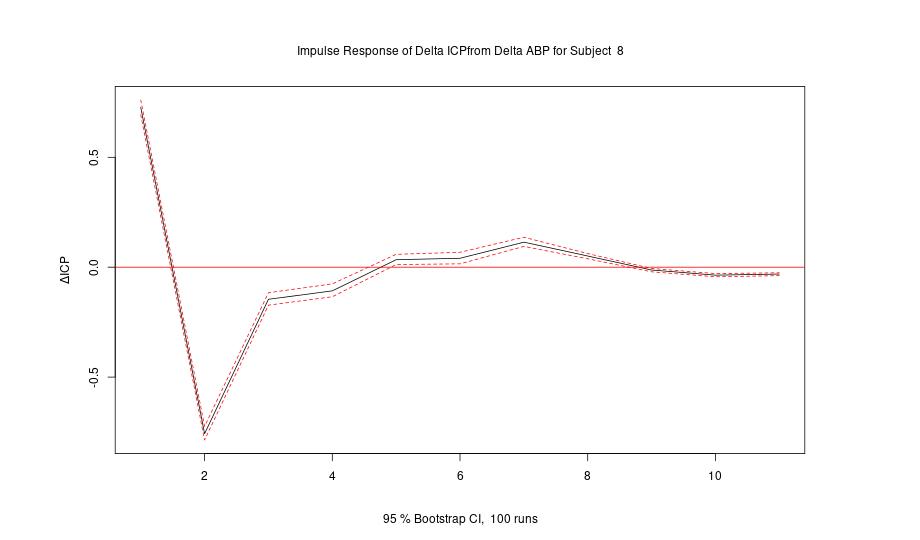

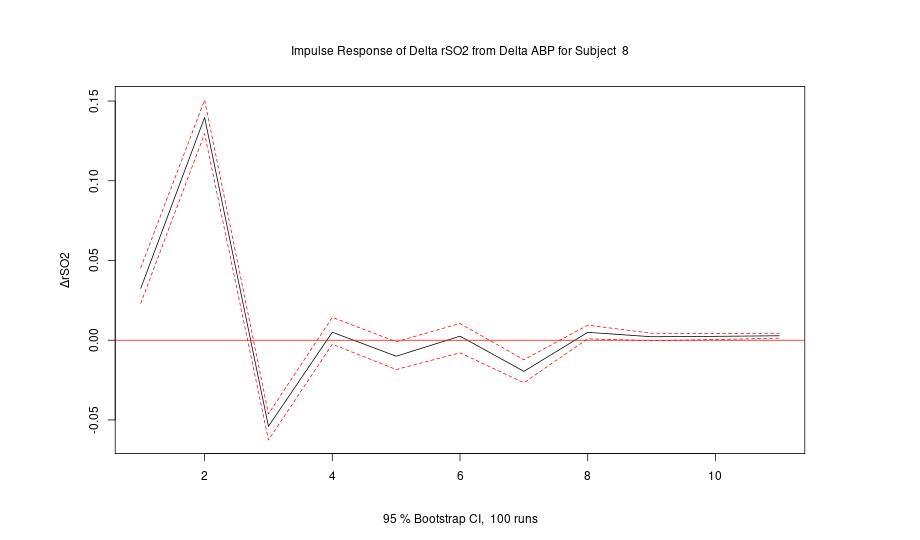


**Figure S8:** *Top panel shows the modeled resulting response in change in intracranial pressure (ΔICP) to an orthogonal impulse in change in arterial blood pressure (ΔABP). Bottom panel shows the modeled resulting response in change in regional cerebral oxygen saturation (ΔrSO_2_) to an orthogonal impulse in change in arterial blood pressure (ΔABP). The 95% confidence intervals are indicated by the red dashed line. Note the dissimilar pattern of phases.*


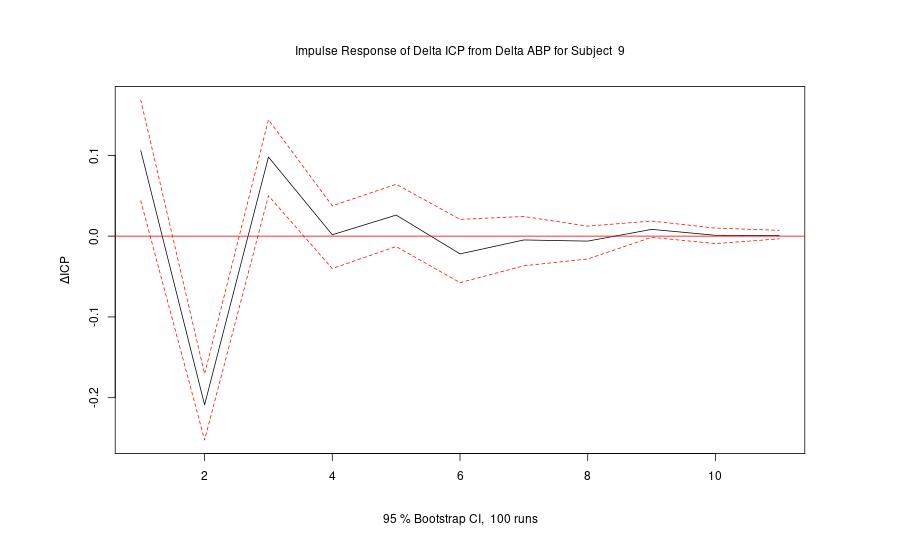

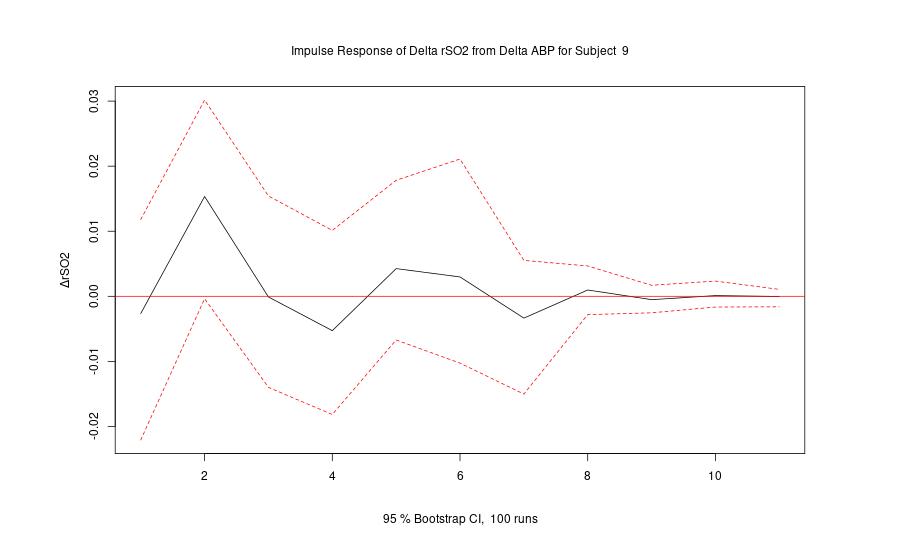


**Figure S9:** *Top panel shows the modeled resulting response in change in intracranial pressure (ΔICP) to an orthogonal impulse in change in arterial blood pressure (ΔABP). Bottom panel shows the modeled resulting response in change in regional cerebral oxygen saturation (ΔrSO_2_) to an orthogonal impulse in change in arterial blood pressure (ΔABP). The 95% confidence intervals are indicated by the red dashed line. Note the dissimilar pattern of phases.*


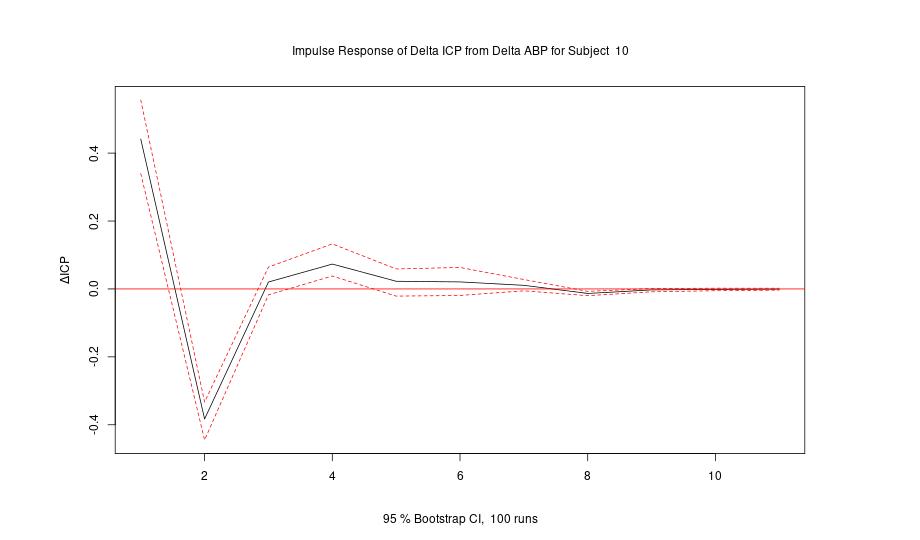

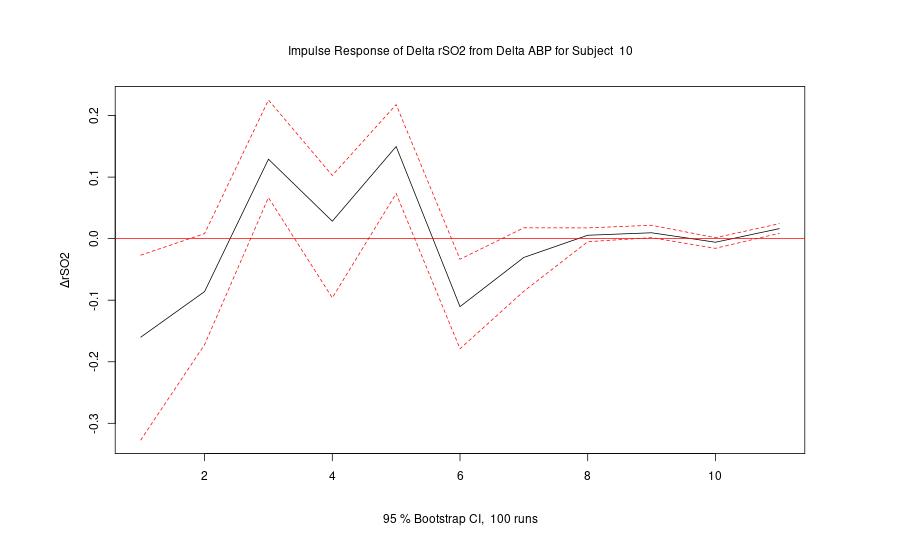


**Figure S10:** *Top panel shows the modeled resulting response in change in intracranial pressure (ΔICP) to an orthogonal impulse in change in arterial blood pressure (ΔABP). Bottom panel shows the modeled resulting response in change in regional cerebral oxygen saturation (ΔrSO_2_) to an orthogonal impulse in change in arterial blood pressure (ΔABP). The 95% confidence intervals are indicated by the red dashed line. Note the dissimilar pattern of phases.*


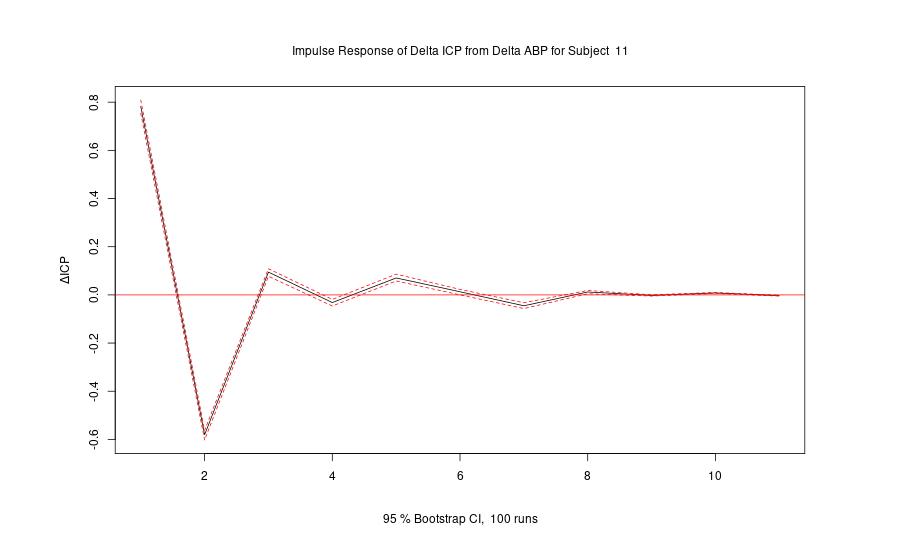

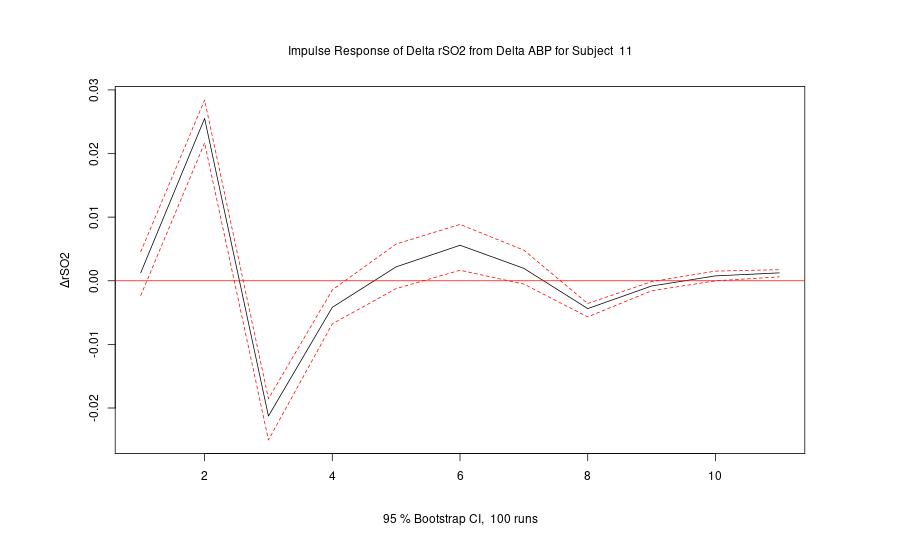


**Figure S11:** *Top panel shows the modeled resulting response in change in intracranial pressure (ΔICP) to an orthogonal impulse in change in arterial blood pressure (ΔABP). Bottom panel shows the modeled resulting response in change in regional cerebral oxygen saturation (ΔrSO_2_) to an orthogonal impulse in change in arterial blood pressure (ΔABP). The 95% confidence intervals are indicated by the red dashed line. Note the similar pattern of phases.*


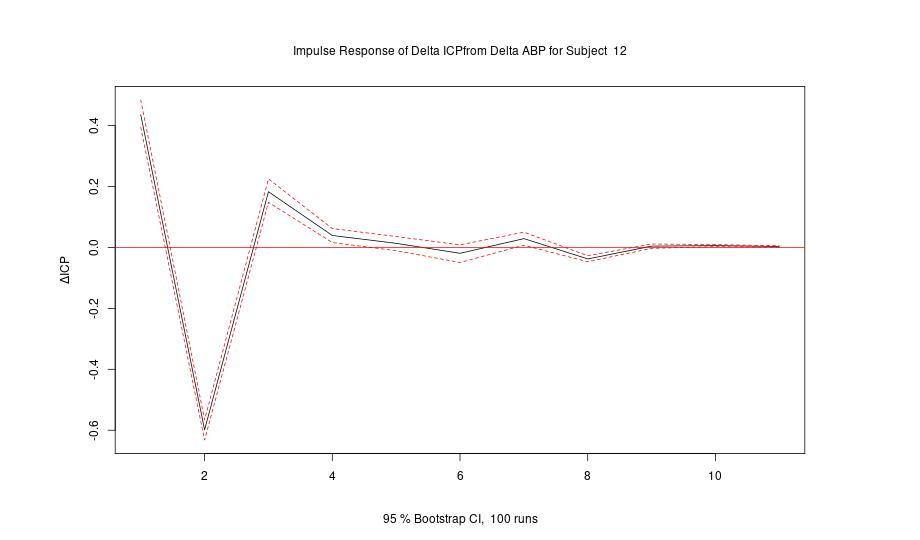


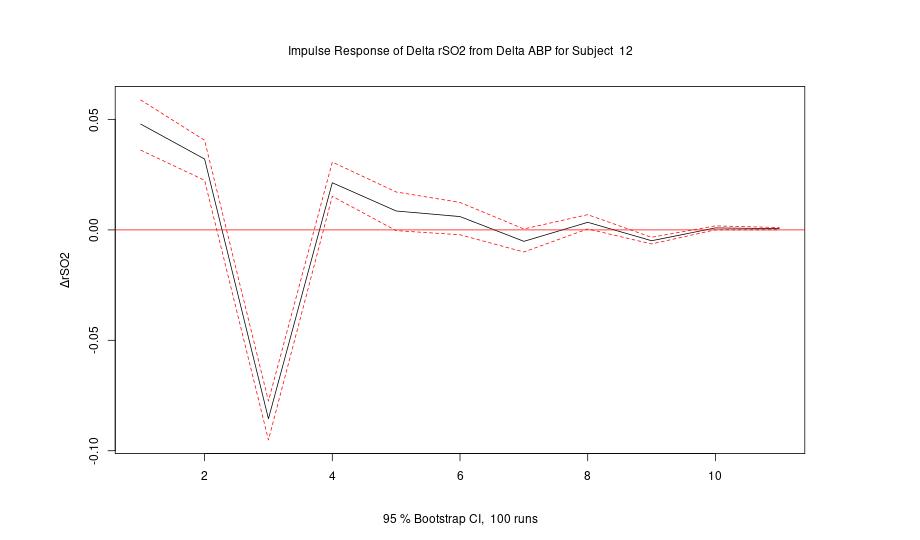


**Figure S12:** *Top panel shows the modeled resulting response in change in intracranial pressure (ΔICP) to an orthogonal impulse in change in arterial blood pressure (ΔABP). Bottom panel shows the modeled resulting response in change in regional cerebral oxygen saturation (ΔrSO_2_) to an orthogonal impulse in change in arterial blood pressure (ΔABP). The 95% confidence intervals are indicated by the red dashed line. Note the similar pattern of phases.*


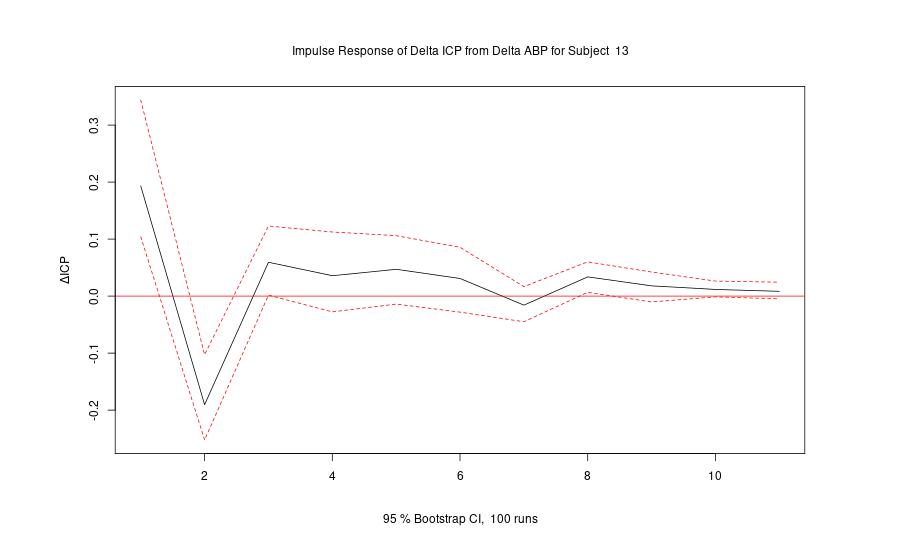

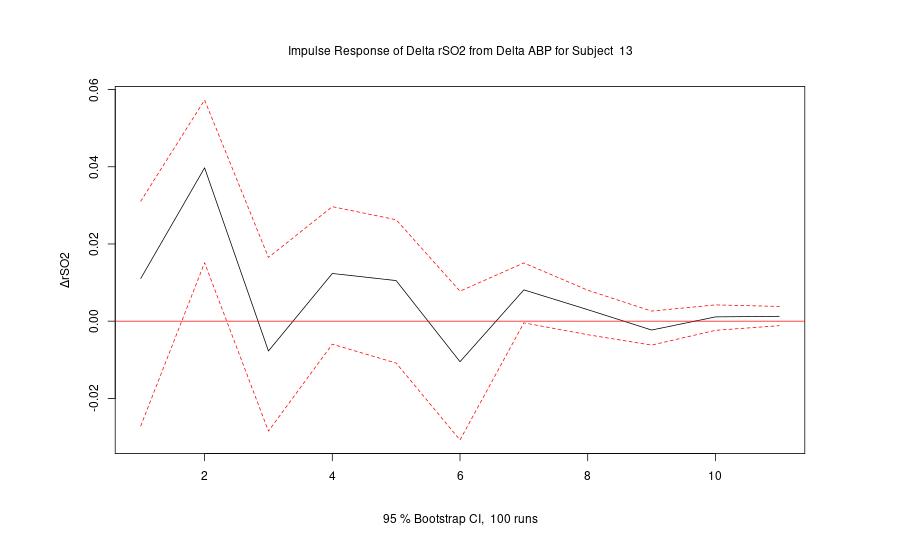


**Figure S13:** *Top panel shows the modeled resulting response in change in intracranial pressure (ΔICP) to an orthogonal impulse in change in arterial blood pressure (ΔABP). Bottom panel shows the modeled resulting response in change in regional cerebral oxygen saturation (ΔrSO_2_) to an orthogonal impulse in change in arterial blood pressure (ΔABP). The 95% confidence intervals are indicated by the red dashed line. Note the dissimilar pattern of phases.*


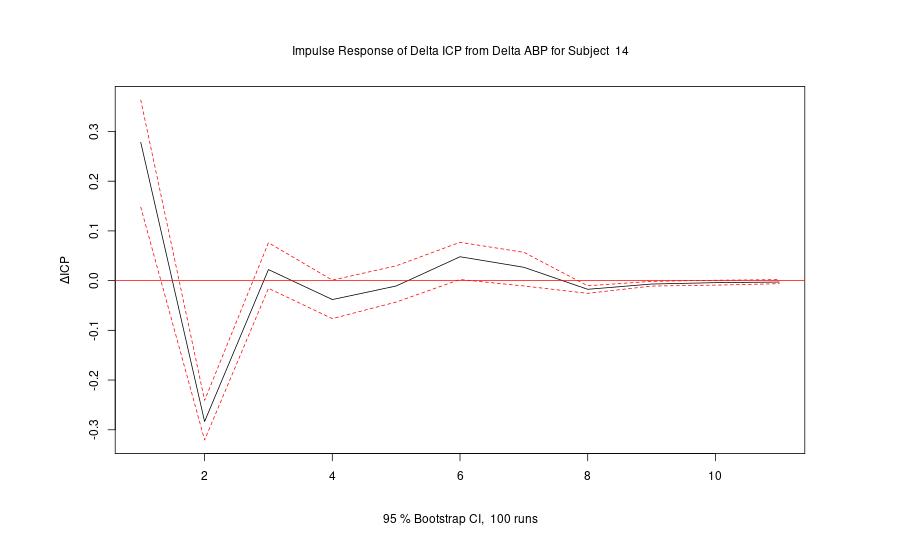

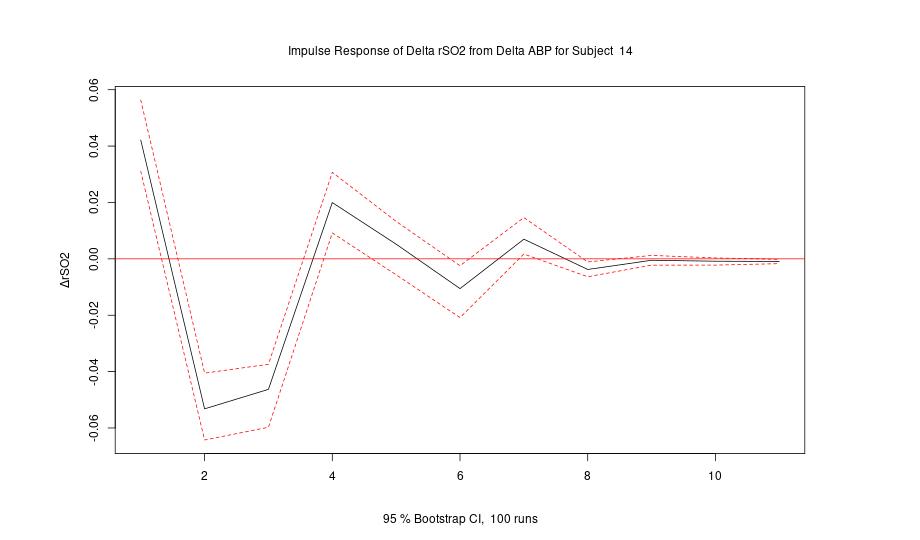


**Figure S14:** *Top panel shows the modeled resulting response in change in intracranial pressure (ΔICP) to an orthogonal impulse in change in arterial blood pressure (ΔABP). Bottom panel shows the modeled resulting response in change in regional cerebral oxygen saturation (ΔrSO_2_) to an orthogonal impulse in change in arterial blood pressure (ΔABP). The 95% confidence intervals are indicated by the red dashed line. Note the similar pattern of phases.*


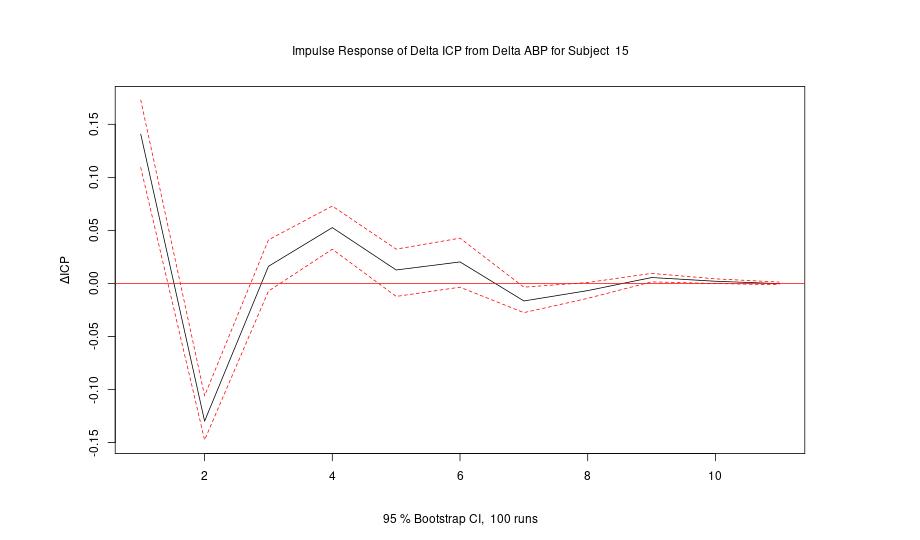

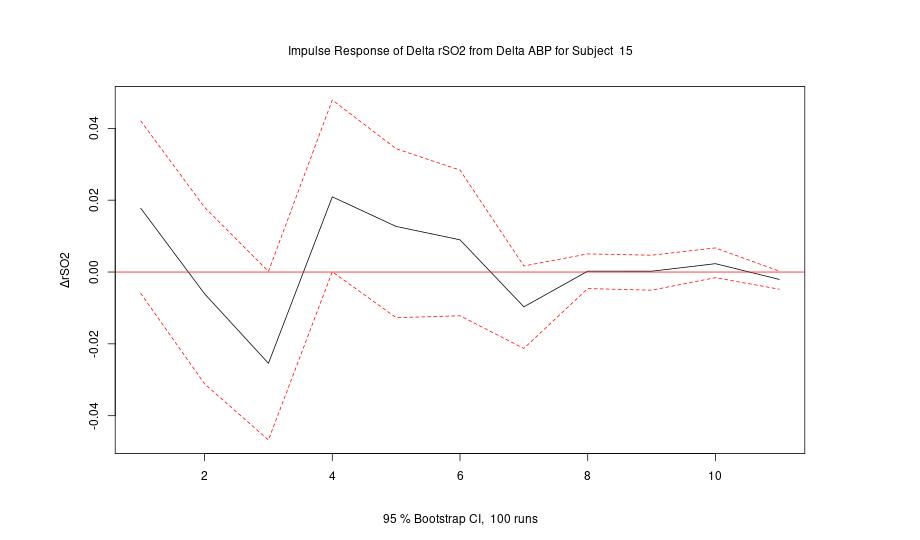


**Figure S15:** *Top panel shows the modeled resulting response in change in intracranial pressure (ΔICP) to an orthogonal impulse in change in arterial blood pressure (ΔABP). Bottom panel shows the modeled resulting response in change in regional cerebral oxygen saturation (ΔrSO_2_) to an orthogonal impulse in change in arterial blood pressure (ΔABP). The 95% confidence intervals are indicated by the red dashed line. Note the similar pattern of phases.*


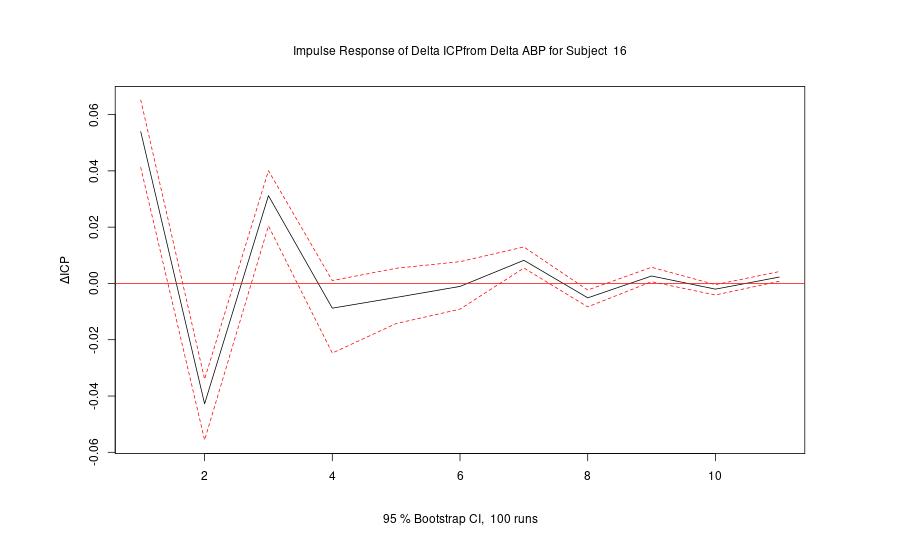

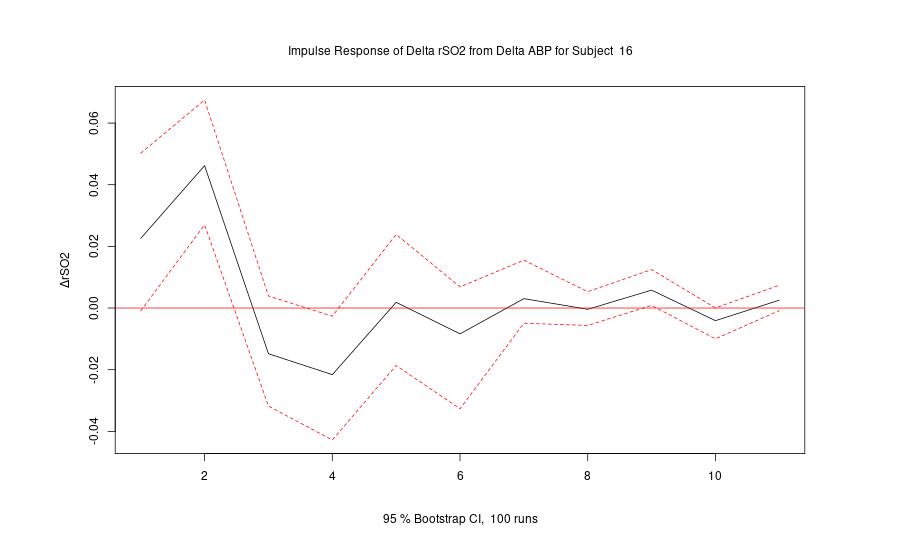


**Figure S16:** *Top panel shows the modeled resulting response in change in intracranial pressure (ΔICP) to an orthogonal impulse in change in arterial blood pressure (ΔABP). Bottom panel shows the modeled resulting response in change in regional cerebral oxygen saturation (ΔrSO_2_) to an orthogonal impulse in change in arterial blood pressure (ΔABP). The 95% confidence intervals are indicated by the red dashed line. Note the dissimilar pattern of phases.*


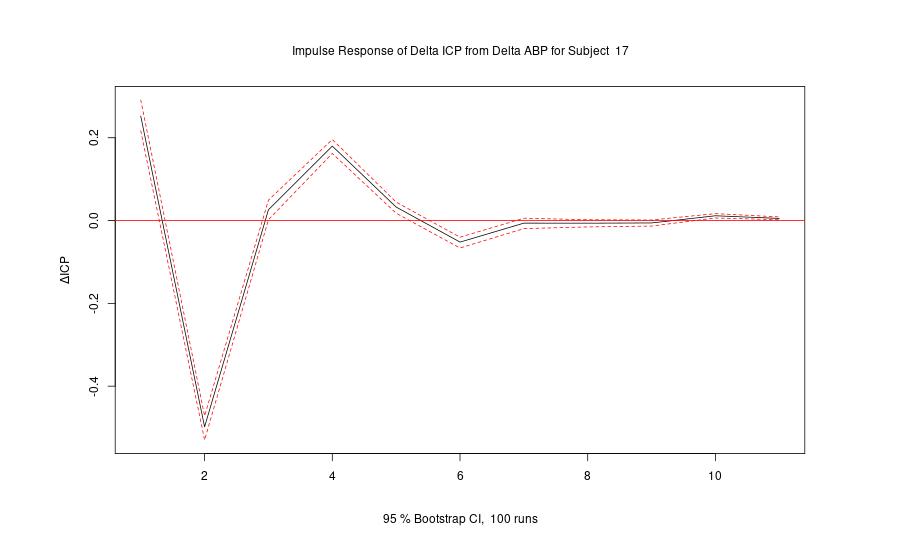

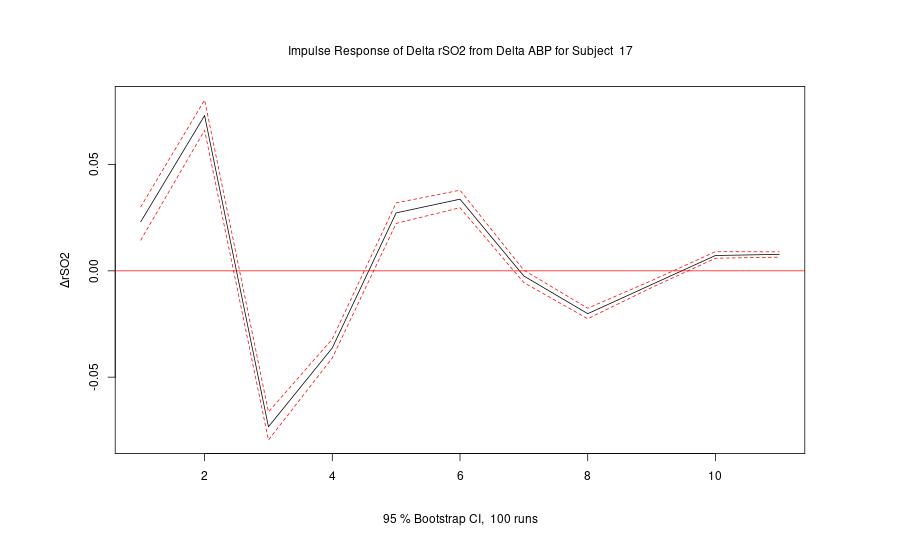


**Figure S17:** *Top panel shows the modeled resulting response in change in intracranial pressure (ΔICP) to an orthogonal impulse in change in arterial blood pressure (ΔABP). Bottom panel shows the modeled resulting response in change in regional cerebral oxygen saturation (ΔrSO_2_) to an orthogonal impulse in change in arterial blood pressure (ΔABP). The 95% confidence intervals are indicated by the red dashed line. Note the similar pattern of phases.*


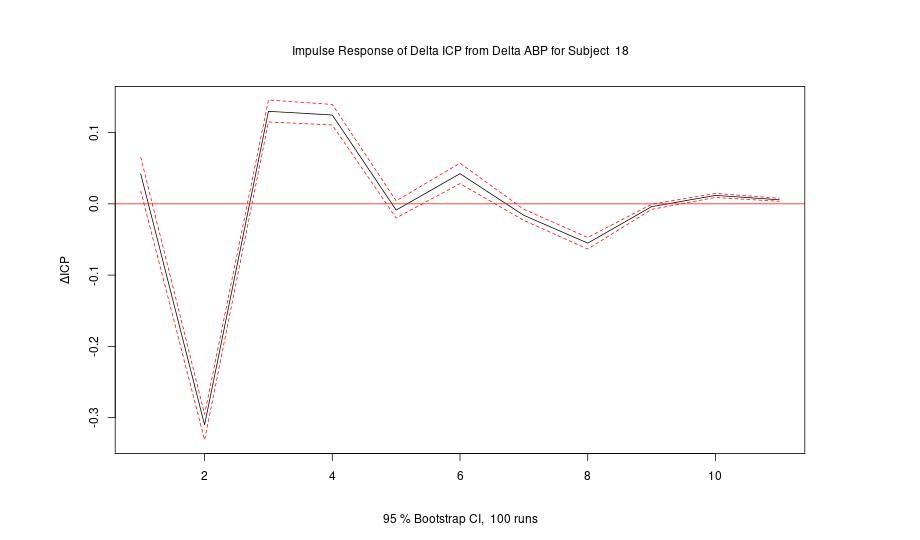

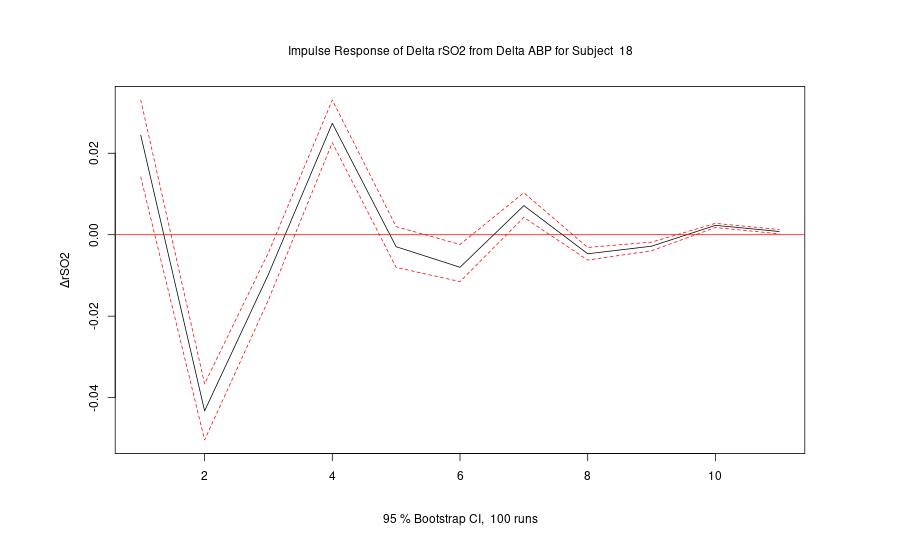


**Figure S18:** *Top panel shows the modeled resulting response in change in intracranial pressure (ΔICP) to an orthogonal impulse in change in arterial blood pressure (ΔABP). Bottom panel shows the modeled resulting response in change in regional cerebral oxygen saturation (ΔrSO_2_) to an orthogonal impulse in change in arterial blood pressure (ΔABP). The 95% confidence intervals are indicated by the red dashed line. Note the similar pattern of phases.*


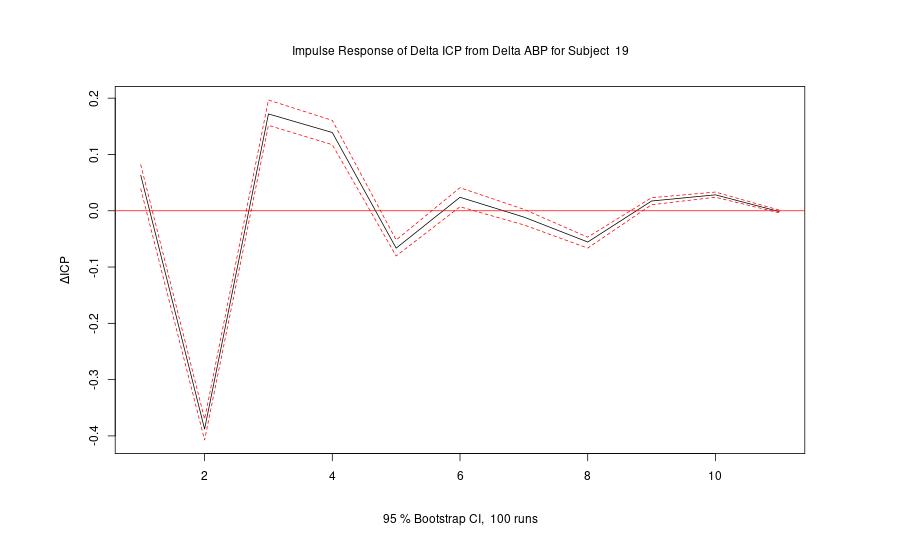

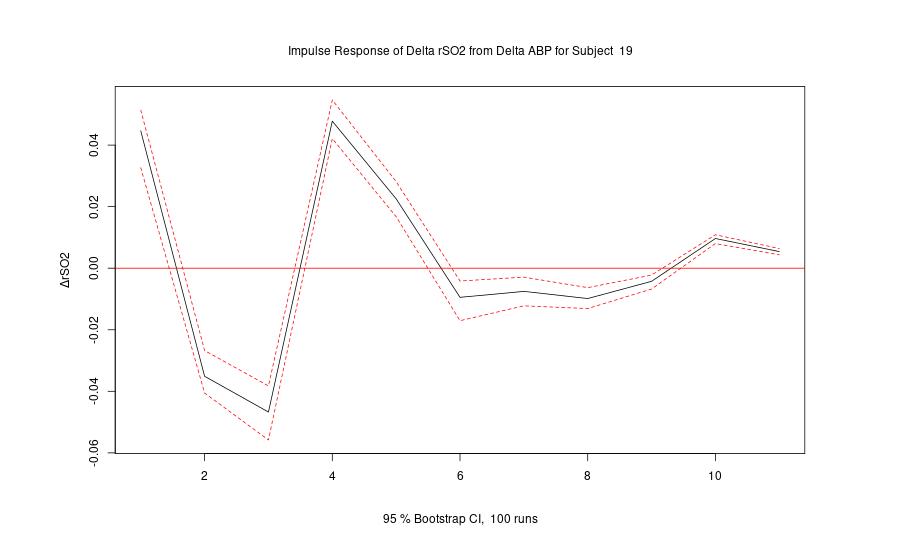


**Figure S19:** *Top panel shows the modeled resulting response in change in intracranial pressure (ΔICP) to an orthogonal impulse in change in arterial blood pressure (ΔABP). Bottom panel shows the modeled resulting response in change in regional cerebral oxygen saturation (ΔrSO_2_) to an orthogonal impulse in change in arterial blood pressure (ΔABP). The 95% confidence intervals are indicated by the red dashed line. Note the similar pattern of phases.*


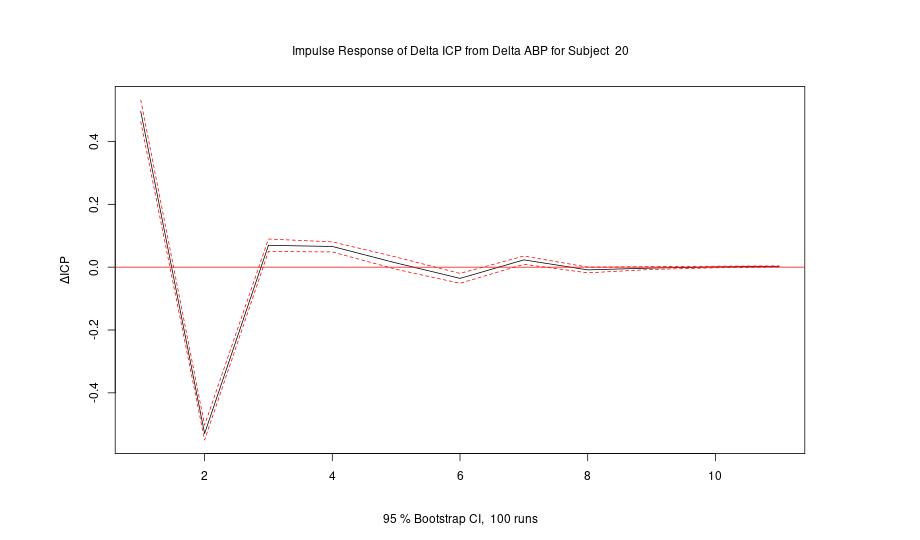

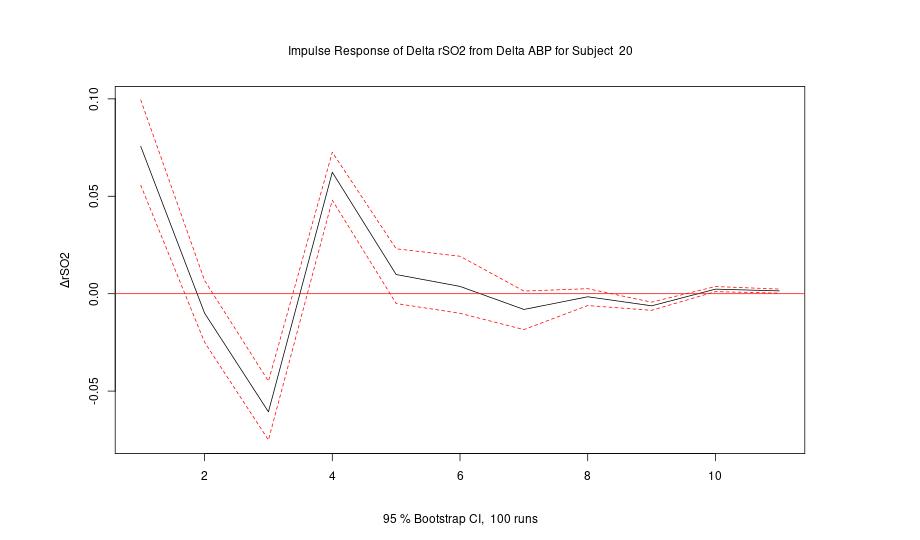


**Figure S20:** *Top panel shows the modeled resulting response in change in intracranial pressure (ΔICP) to an orthogonal impulse in change in arterial blood pressure (ΔABP). Bottom panel shows the modeled resulting response in change in regional cerebral oxygen saturation (ΔrSO_2_) to an orthogonal impulse in change in arterial blood pressure (ΔABP). The 95% confidence intervals are indicated by the red dashed line. Note the similar pattern of phases.*


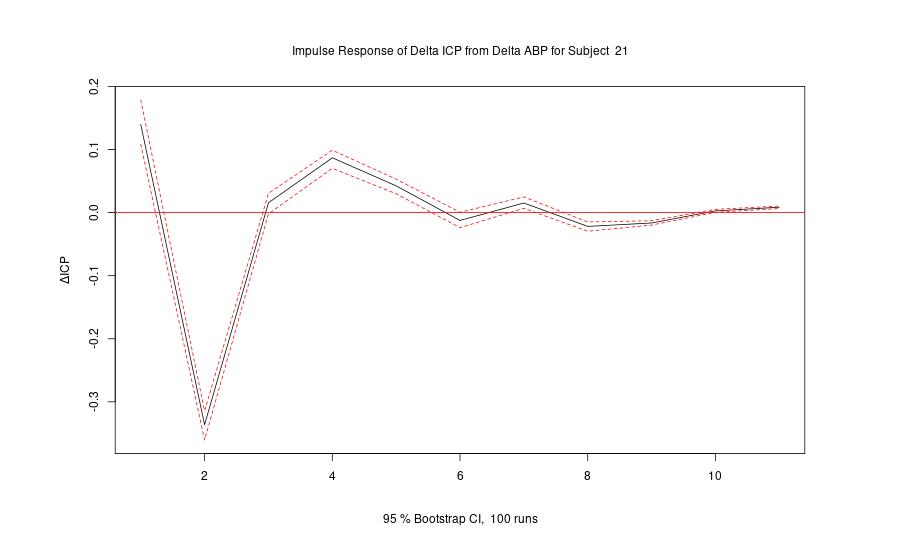

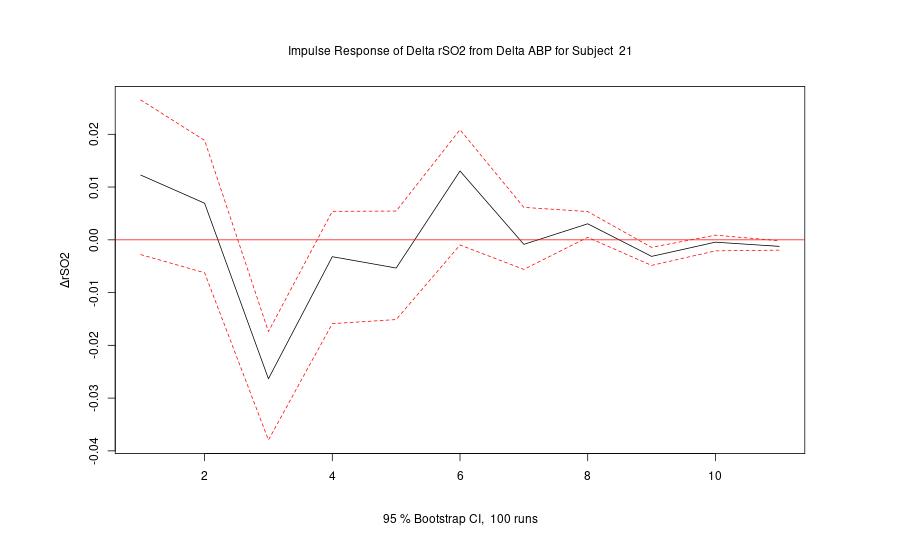


**Figure S21:** *Top panel shows the modeled resulting response in change in intracranial pressure (ΔICP) to an orthogonal impulse in change in arterial blood pressure (ΔABP). Bottom panel shows the modeled resulting response in change in regional cerebral oxygen saturation (ΔrSO_2_) to an orthogonal impulse in change in arterial blood pressure (ΔABP). The 95% confidence intervals are indicated by the red dashed line. Note the similar pattern of phases.*


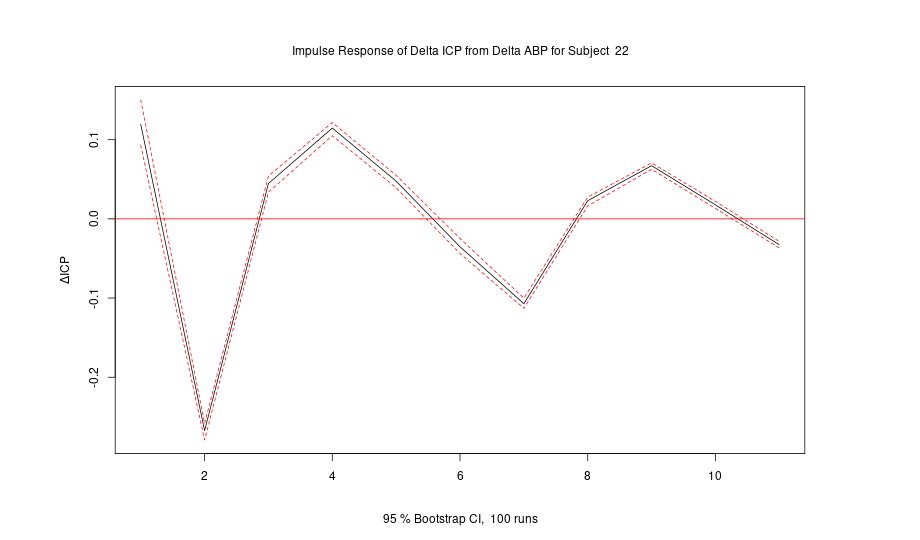

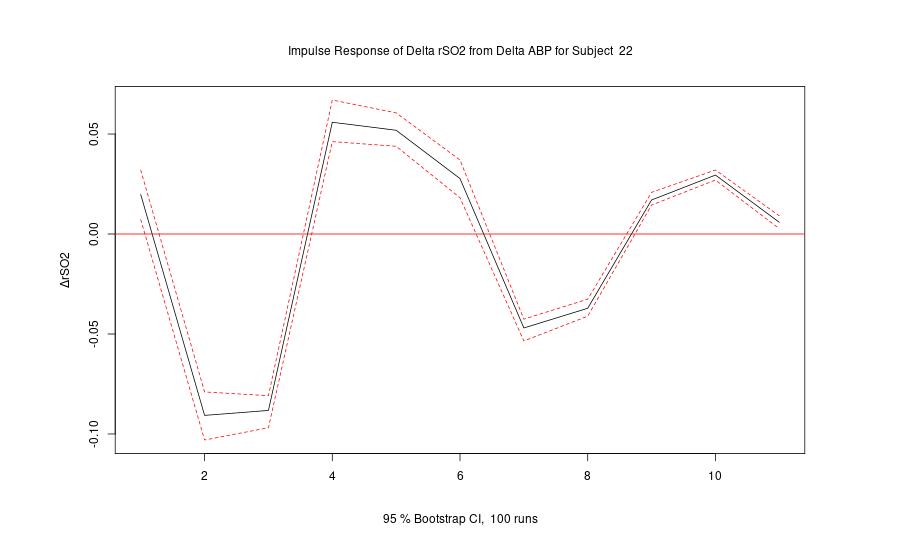


**Figure S22:** *Top panel shows the modeled resulting response in change in intracranial pressure (ΔICP) to an orthogonal impulse in change in arterial blood pressure (ΔABP). Bottom panel shows the modeled resulting response in change in regional cerebral oxygen saturation (ΔrSO_2_) to an orthogonal impulse in change in arterial blood pressure (ΔABP). The 95% confidence intervals are indicated by the red dashed line. Note the similar pattern of phases.*


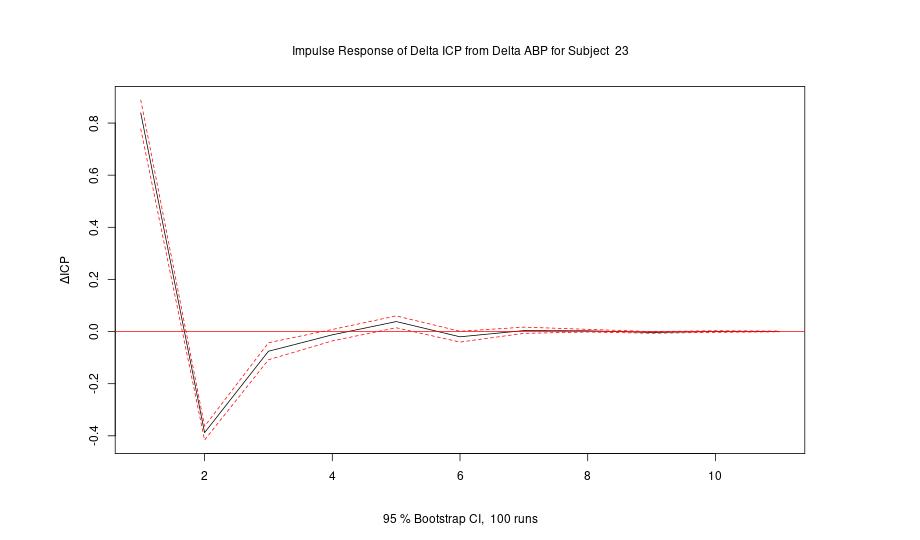

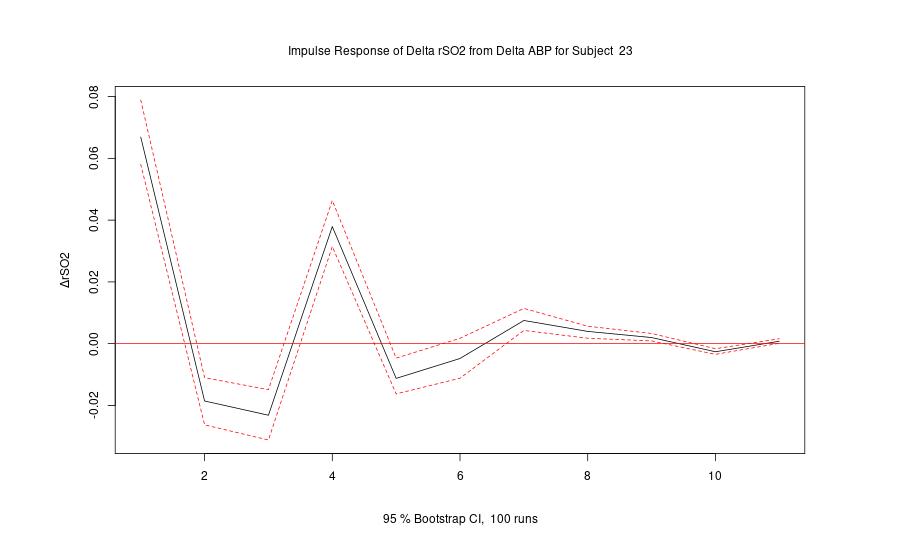


**Figure S23:** *Top panel shows the modeled resulting response in change in intracranial pressure (ΔICP) to an orthogonal impulse in change in arterial blood pressure (ΔABP). Bottom panel shows the modeled resulting response in change in regional cerebral oxygen saturation (ΔrSO_2_) to an orthogonal impulse in change in arterial blood pressure (ΔABP). The 95% confidence intervals are indicated by the red dashed line. Note the similar pattern of phases.*


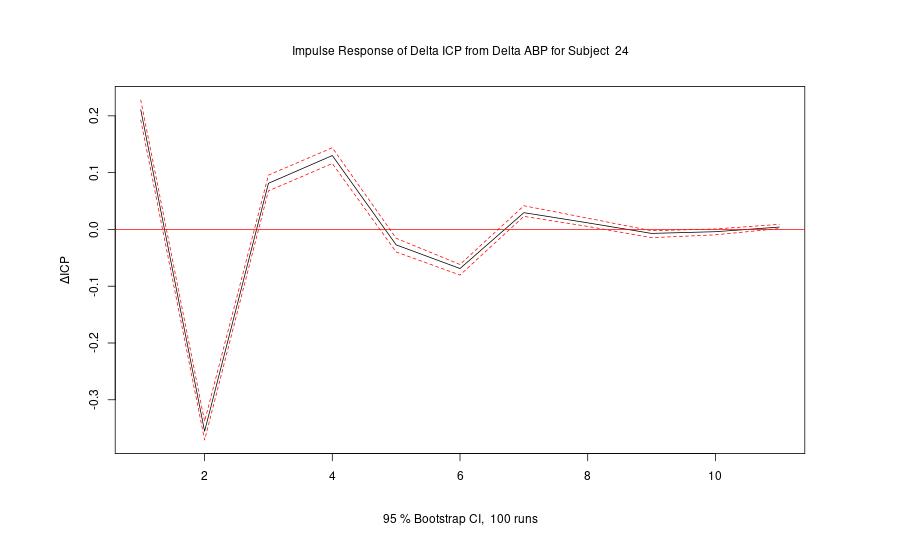

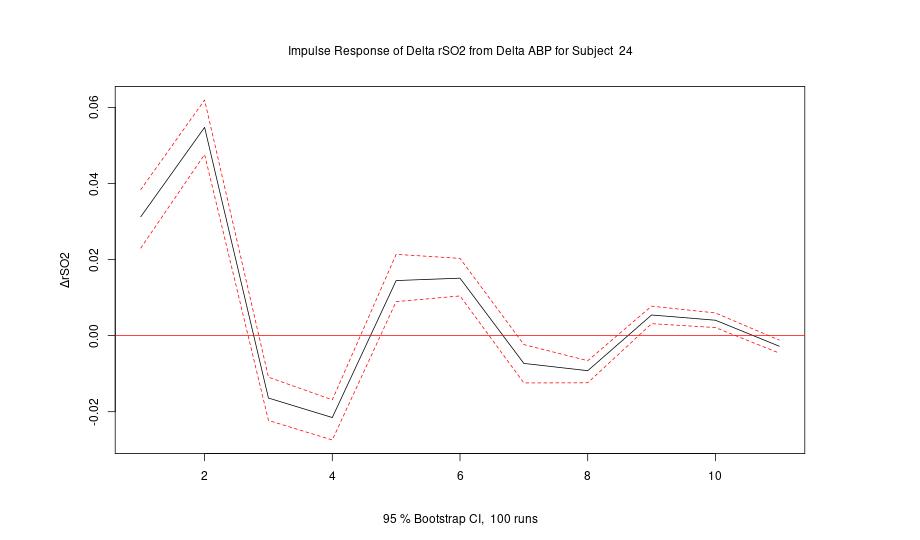


**Figure S24:** *Top panel shows the modeled resulting response in change in intracranial pressure (ΔICP) to an orthogonal impulse in change in arterial blood pressure (ΔABP). Bottom panel shows the modeled resulting response in change in regional cerebral oxygen saturation (ΔrSO_2_) to an orthogonal impulse in change in arterial blood pressure (ΔABP). The 95% confidence intervals are indicated by the red dashed line. Note the similar pattern of phases.*


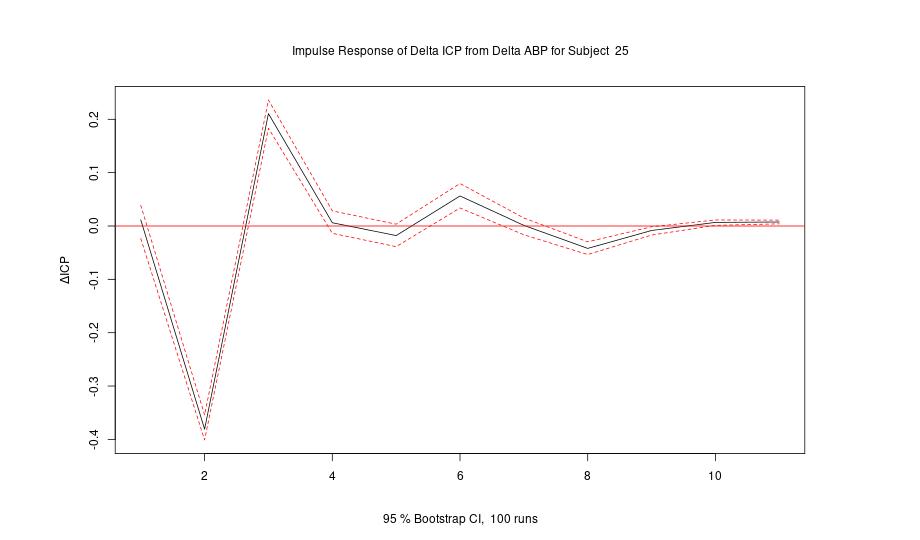

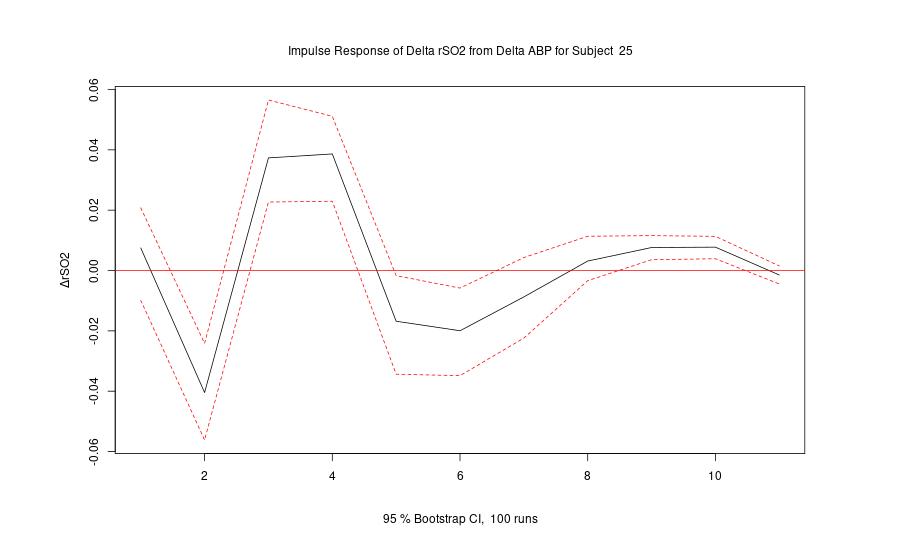


**Figure S25:** *Top panel shows the modeled resulting response in change in intracranial pressure (ΔICP) to an orthogonal impulse in change in arterial blood pressure (ΔABP). Bottom panel shows the modeled resulting response in change in regional cerebral oxygen saturation (ΔrSO_2_) to an orthogonal impulse in change in arterial blood pressure (ΔABP). The 95% confidence intervals are indicated by the red dashed line. Note the similar pattern of phases.*


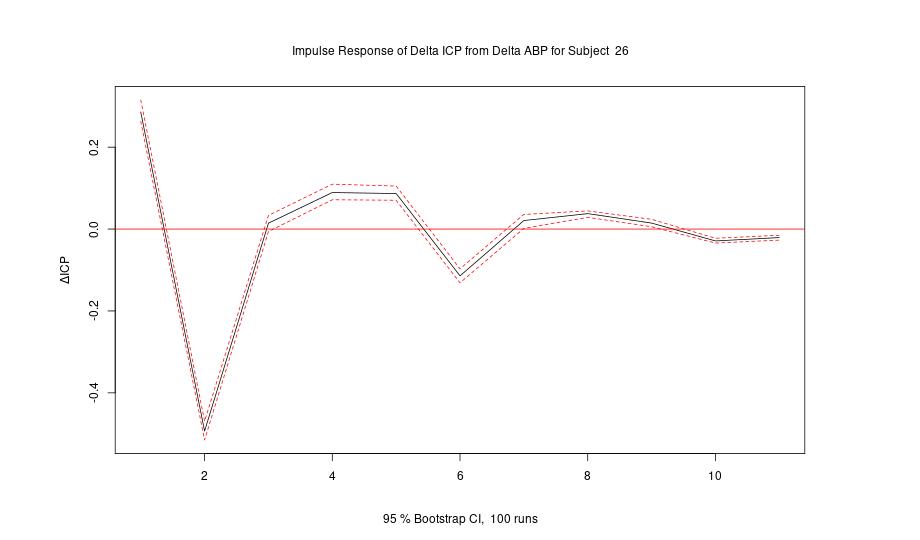

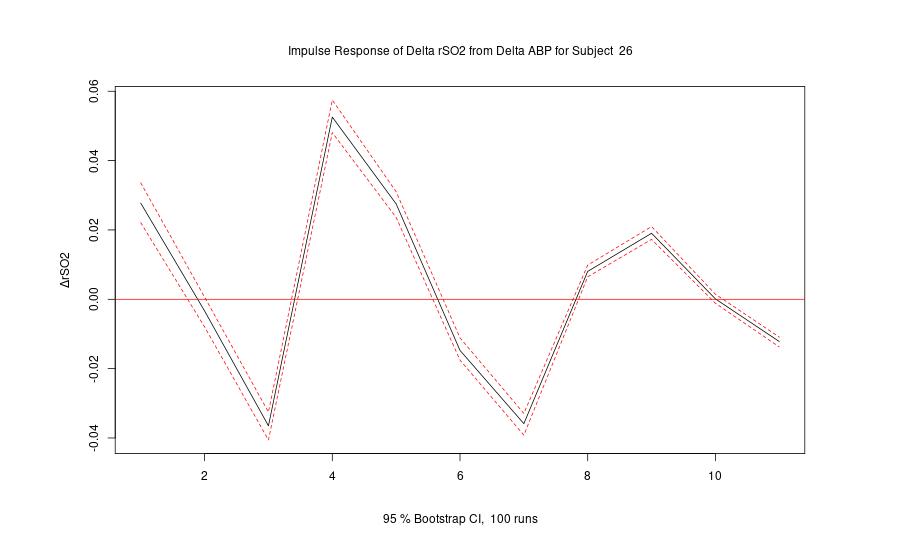


**Figure S26:** *Top panel shows the modeled resulting response in change in intracranial pressure (ΔICP) to an orthogonal impulse in change in arterial blood pressure (ΔABP). Bottom panel shows the modeled resulting response in change in regional cerebral oxygen saturation (ΔrSO_2_) to an orthogonal impulse in change in arterial blood pressure (ΔABP). The 95% confidence intervals are indicated by the red dashed line. Note the similar pattern of phases.*


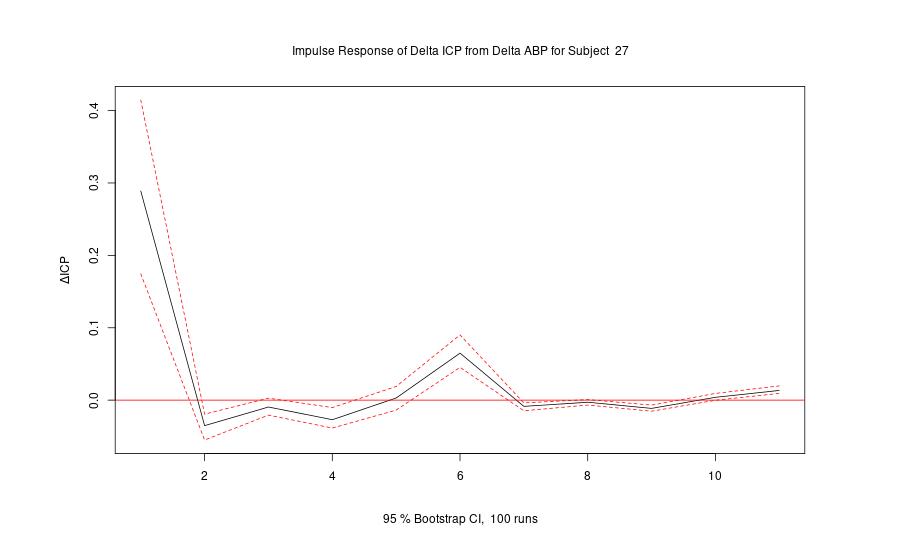

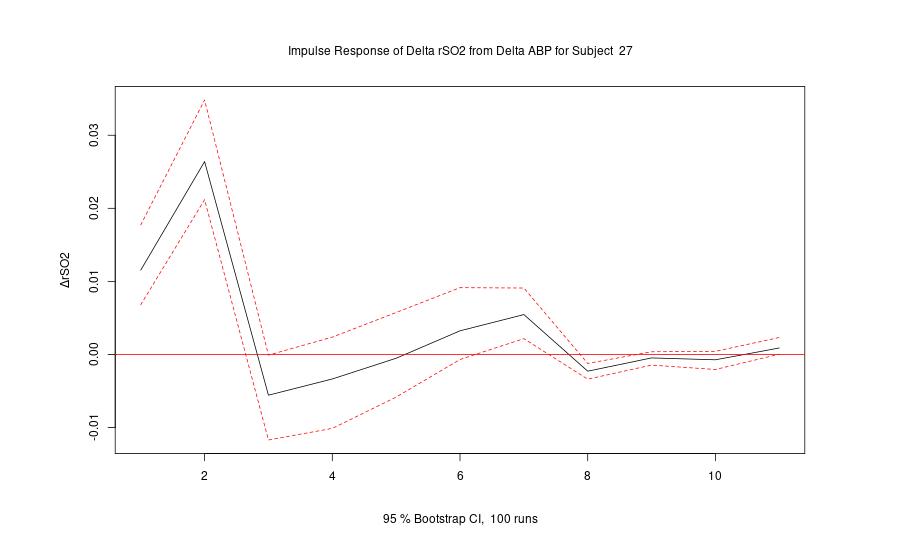


**Figure S27:** *Top panel shows the modeled resulting response in change in intracranial pressure (ΔICP) to an orthogonal impulse in change in arterial blood pressure (ΔABP). Bottom panel shows the modeled resulting response in change in regional cerebral oxygen saturation (ΔrSO_2_) to an orthogonal impulse in change in arterial blood pressure (ΔABP). The 95% confidence intervals are indicated by the red dashed line. Note the similar pattern of phases.*


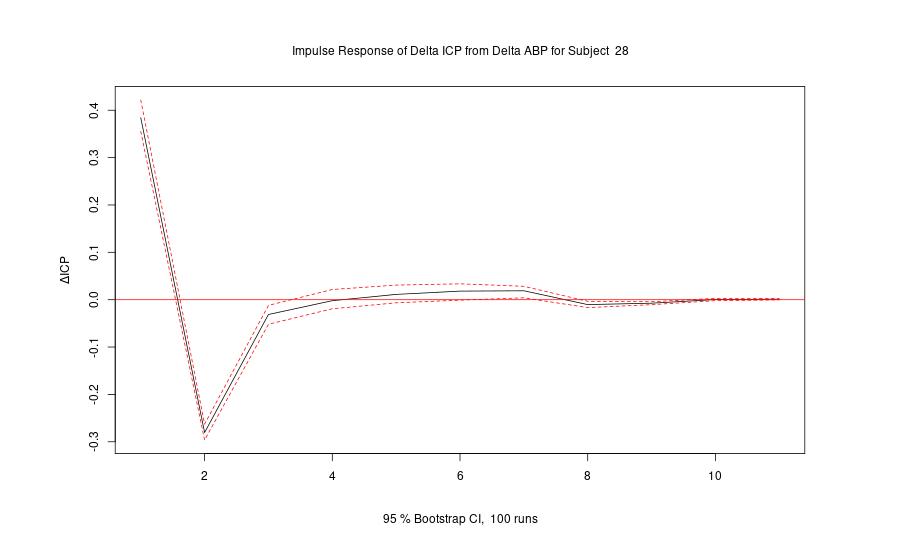

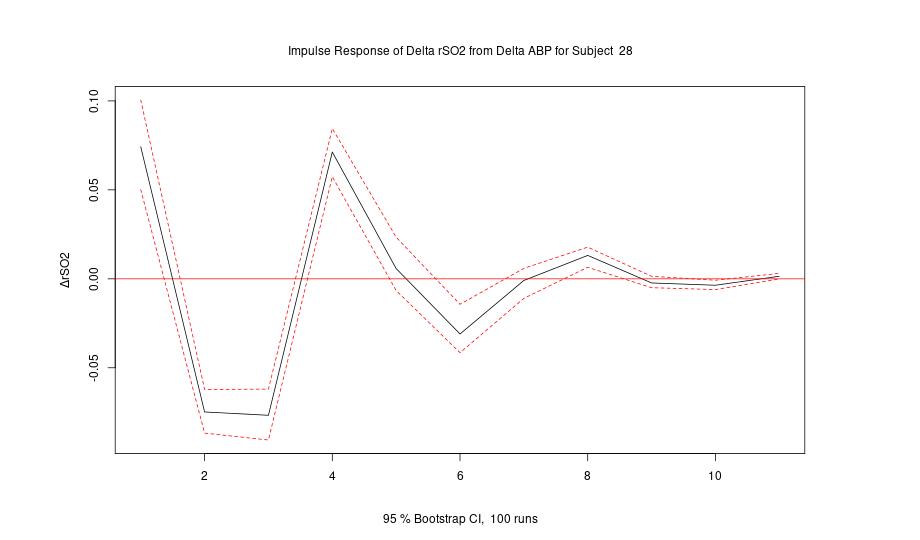


**Figure S28:** *Top panel shows the modeled resulting response in change in intracranial pressure (ΔICP) to an orthogonal impulse in change in arterial blood pressure (ΔABP). Bottom panel shows the modeled resulting response in change in regional cerebral oxygen saturation (ΔrSO_2_) to an orthogonal impulse in change in arterial blood pressure (ΔABP). The 95% confidence intervals are indicated by the red dashed line. Note the similar pattern of phases.*


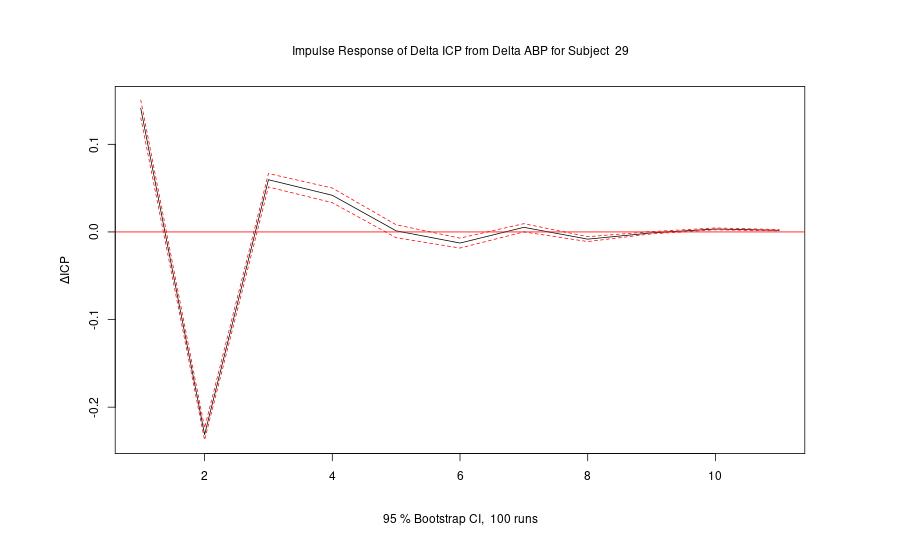

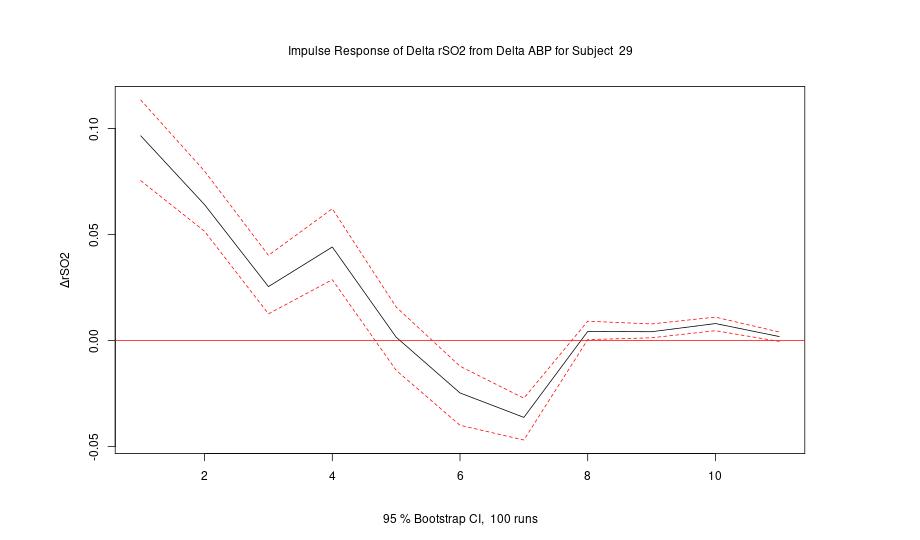


**Figure S29:** *Top panel shows the modeled resulting response in change in intracranial pressure (ΔICP) to an orthogonal impulse in change in arterial blood pressure (ΔABP). Bottom panel shows the modeled resulting response in change in regional cerebral oxygen saturation (ΔrSO_2_) to an orthogonal impulse in change in arterial blood pressure (ΔABP). The 95% confidence intervals are indicated by the red dashed line. Note the dissimilar pattern of phases.*


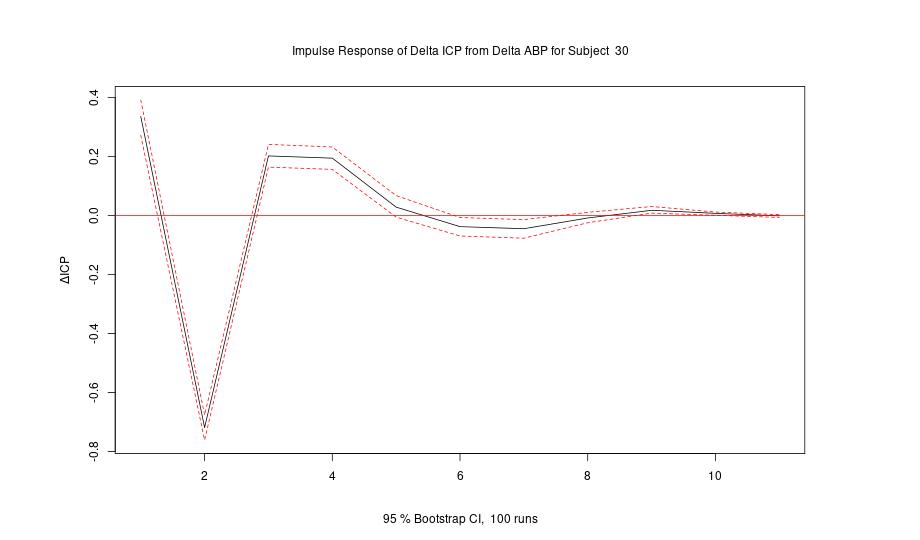

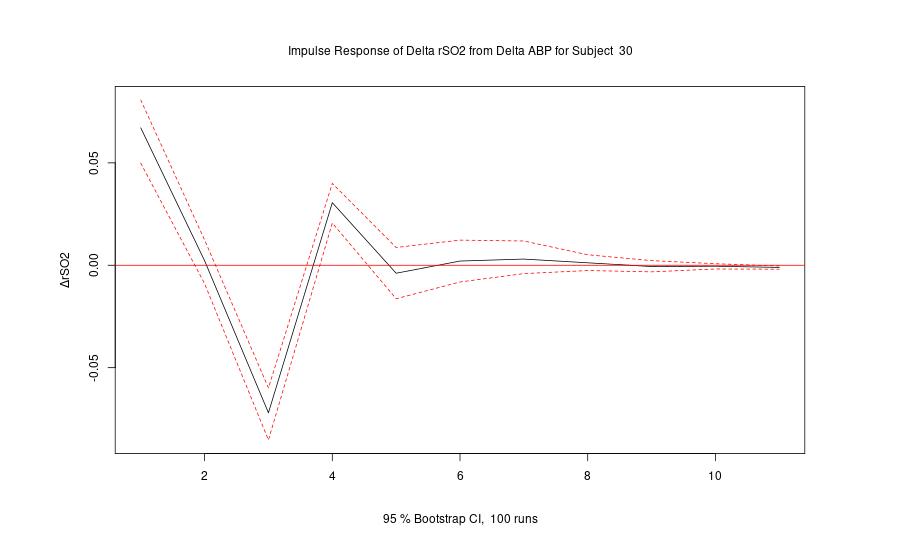


**Figure S30:** *Top panel shows the modeled resulting response in change in intracranial pressure (ΔICP) to an orthogonal impulse in change in arterial blood pressure (ΔABP). Bottom panel shows the modeled resulting response in change in regional cerebral oxygen saturation (ΔrSO_2_) to an orthogonal impulse in change in arterial blood pressure (ΔABP). The 95% confidence intervals are indicated by the red dashed line. Note the similar pattern of phases.*


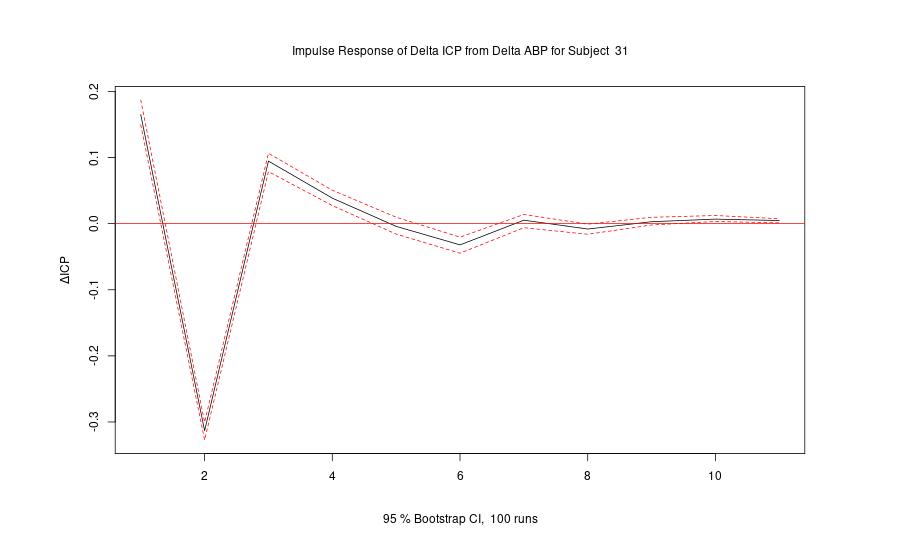

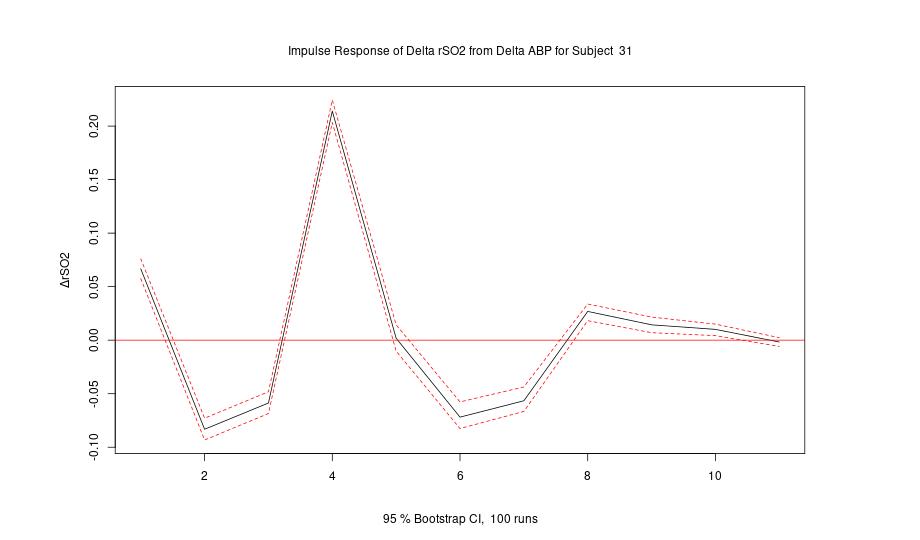


**Figure S31:** *Top panel shows the modeled resulting response in change in intracranial pressure (ΔICP) to an orthogonal impulse in change in arterial blood pressure (ΔABP). Bottom panel shows the modeled resulting response in change in regional cerebral oxygen saturation (ΔrSO_2_) to an orthogonal impulse in change in arterial blood pressure (ΔABP). The 95% confidence intervals are indicated by the red dashed line. Note the similar pattern of phases.*


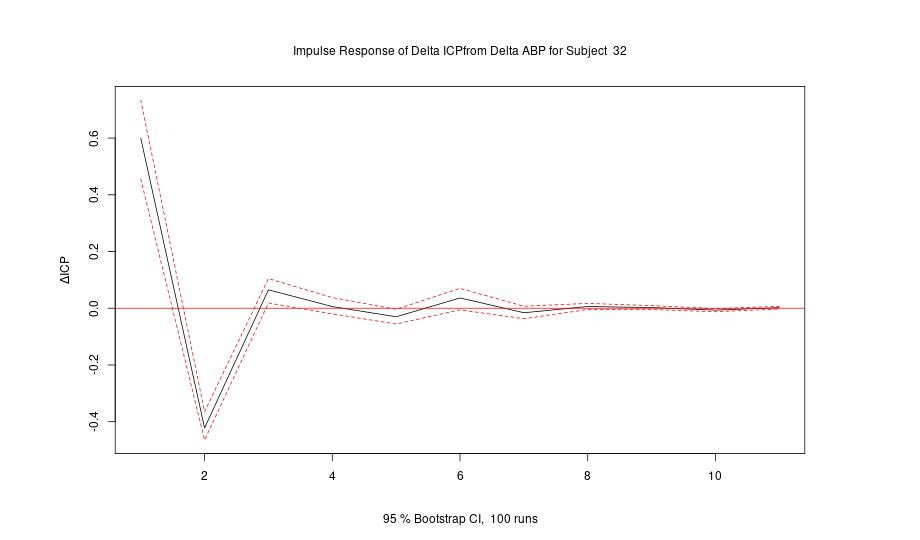

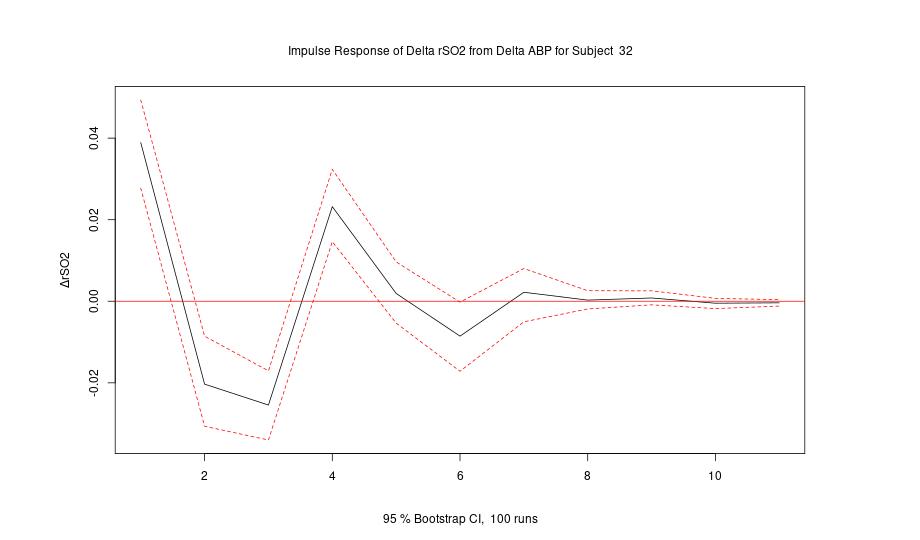


**Figure S32:** *Top panel shows the modeled resulting response in change in intracranial pressure (ΔICP) to an orthogonal impulse in change in arterial blood pressure (ΔABP). Bottom panel shows the modeled resulting response in change in regional cerebral oxygen saturation (ΔrSO_2_) to an orthogonal impulse in change in arterial blood pressure (ΔABP). The 95% confidence intervals are indicated by the red dashed line. Note the similar pattern of phases.*


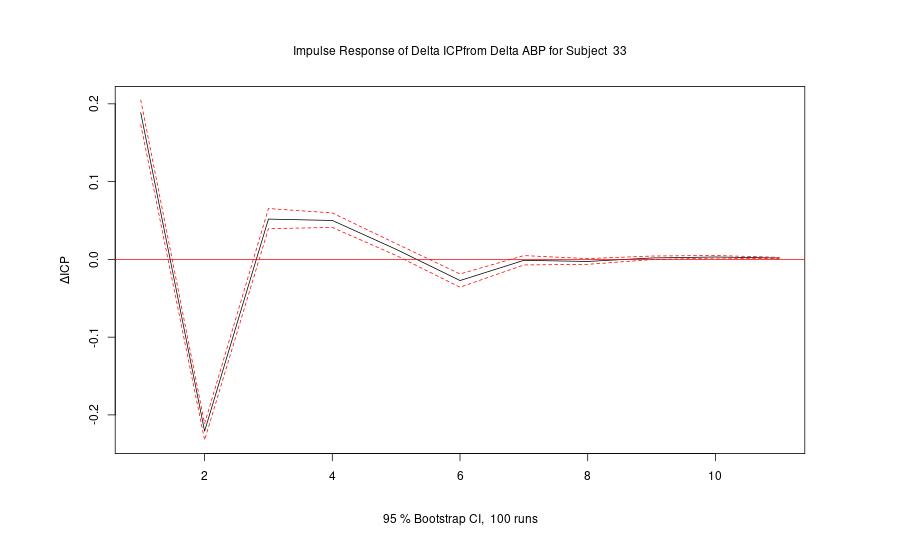

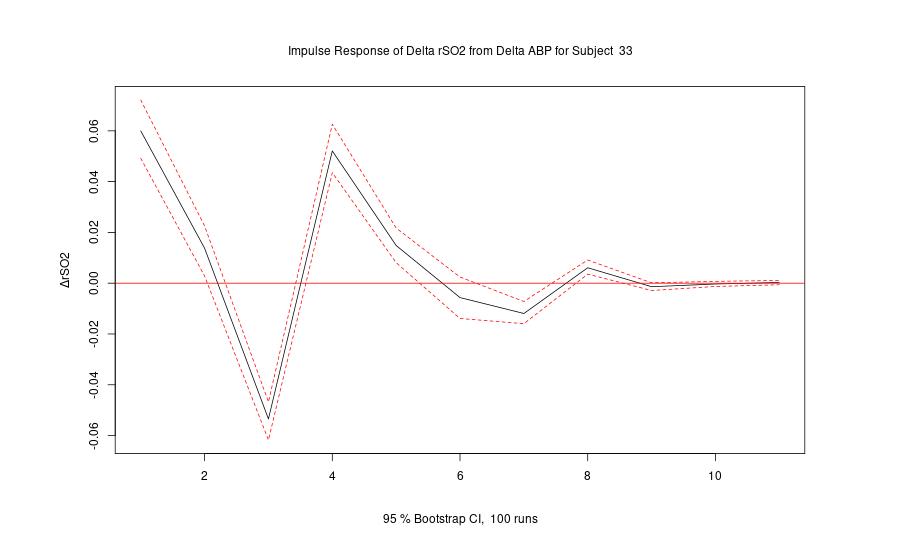


**Figure S33:** *Top panel shows the modeled resulting response in change in intracranial pressure (ΔICP) to an orthogonal impulse in change in arterial blood pressure (ΔABP). Bottom panel shows the modeled resulting response in change in regional cerebral oxygen saturation (ΔrSO_2_) to an orthogonal impulse in change in arterial blood pressure (ΔABP). The 95% confidence intervals are indicated by the red dashed line. Note the similar pattern of phases.*


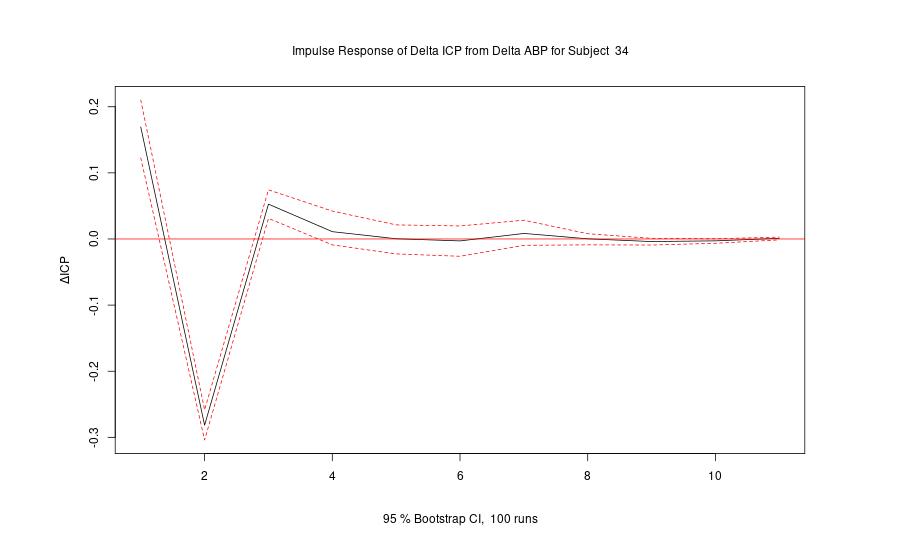

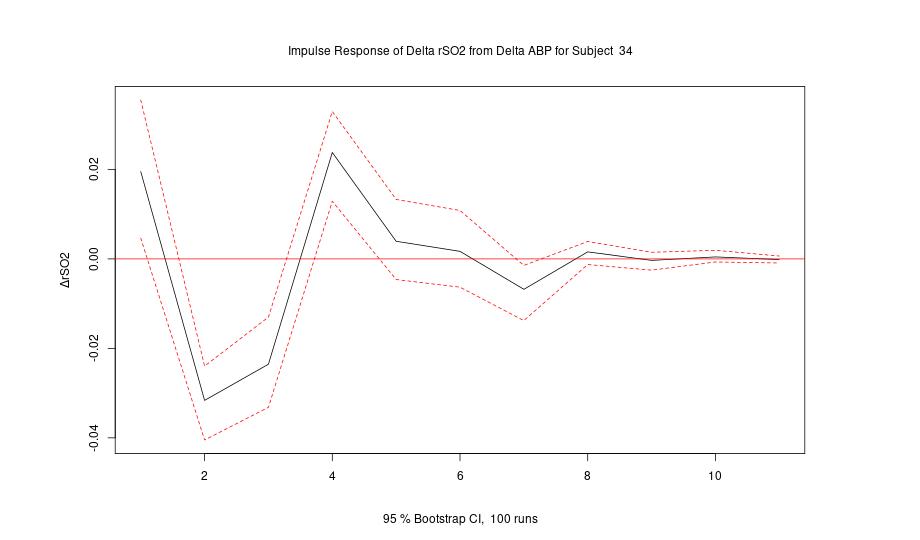


**Figure S34:** *Top panel shows the modeled resulting response in change in intracranial pressure (ΔICP) to an orthogonal impulse in change in arterial blood pressure (ΔABP). Bottom panel shows the modeled resulting response in change in regional cerebral oxygen saturation (ΔrSO_2_) to an orthogonal impulse in change in arterial blood pressure (ΔABP). The 95% confidence intervals are indicated by the red dashed line. Note the similar pattern of phases.*


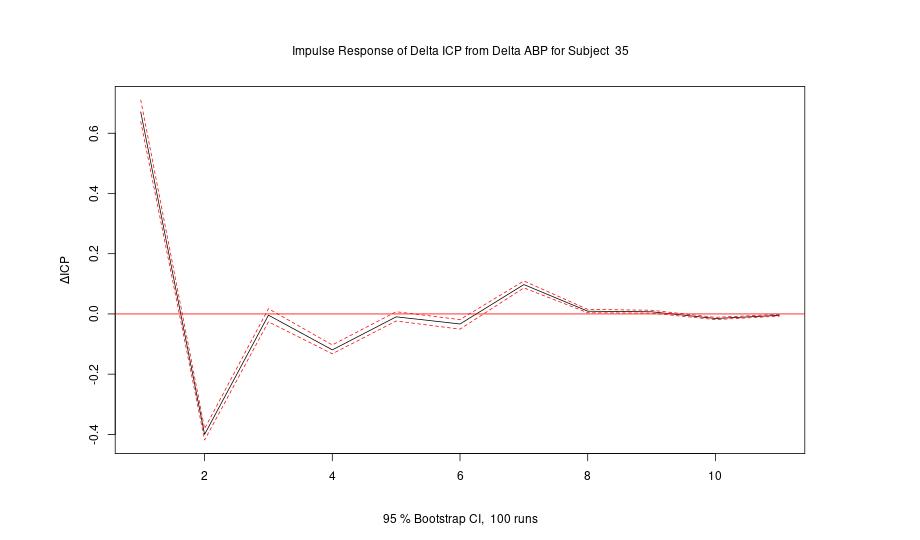

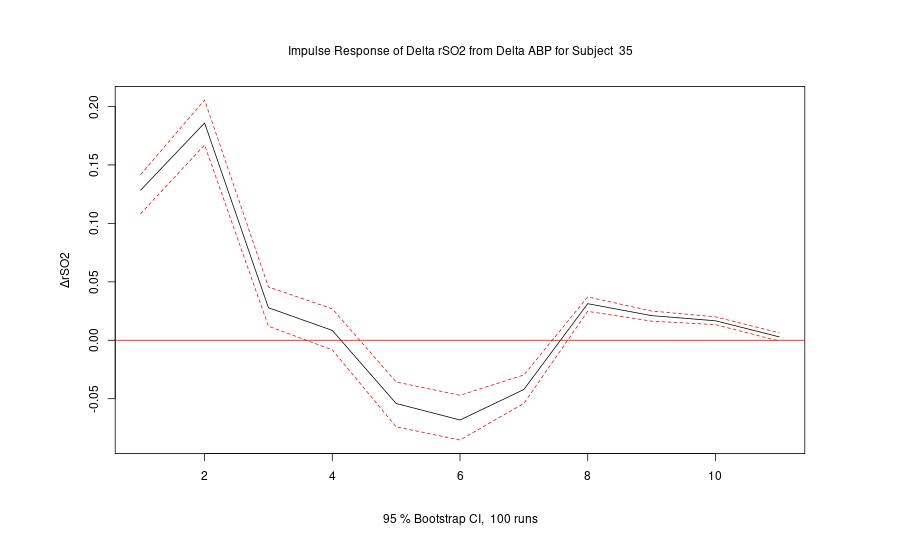


**Figure S35:** *Top panel shows the modeled resulting response in change in intracranial pressure (ΔICP) to an orthogonal impulse in change in arterial blood pressure (ΔABP). Bottom panel shows the modeled resulting response in change in regional cerebral oxygen saturation (ΔrSO_2_) to an orthogonal impulse in change in arterial blood pressure (ΔABP). The 95% confidence intervals are indicated by the red dashed line. Note the dissimilar pattern of phases.*


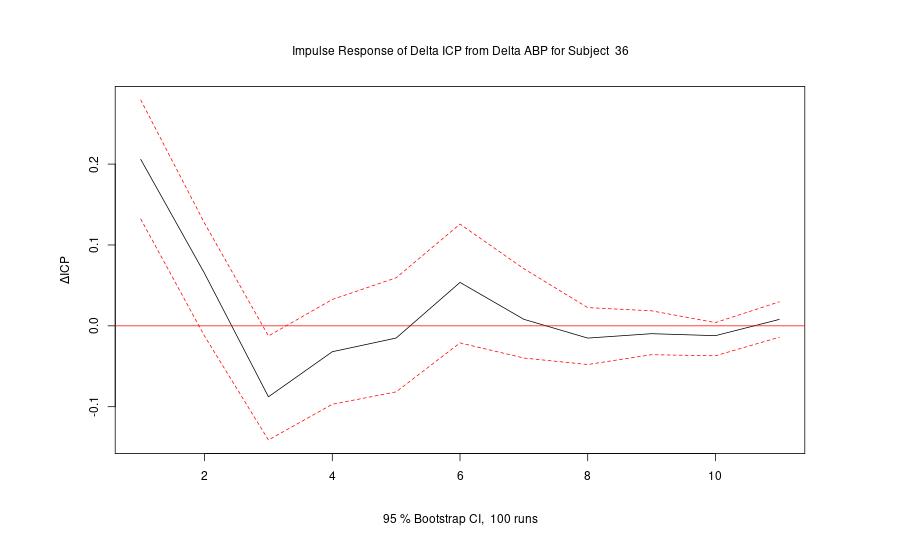

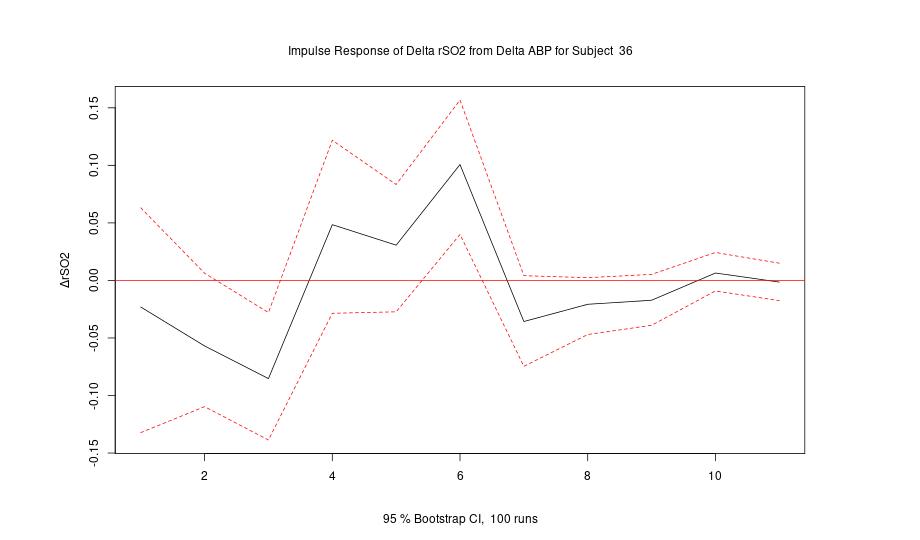


**Figure S36:** *Top panel shows the modeled resulting response in change in intracranial pressure (ΔICP) to an orthogonal impulse in change in arterial blood pressure (ΔABP). Bottom panel shows the modeled resulting response in change in regional cerebral oxygen saturation (ΔrSO_2_) to an orthogonal impulse in change in arterial blood pressure (ΔABP). The 95% confidence intervals are indicated by the red dashed line. Note the similar pattern of phases.*


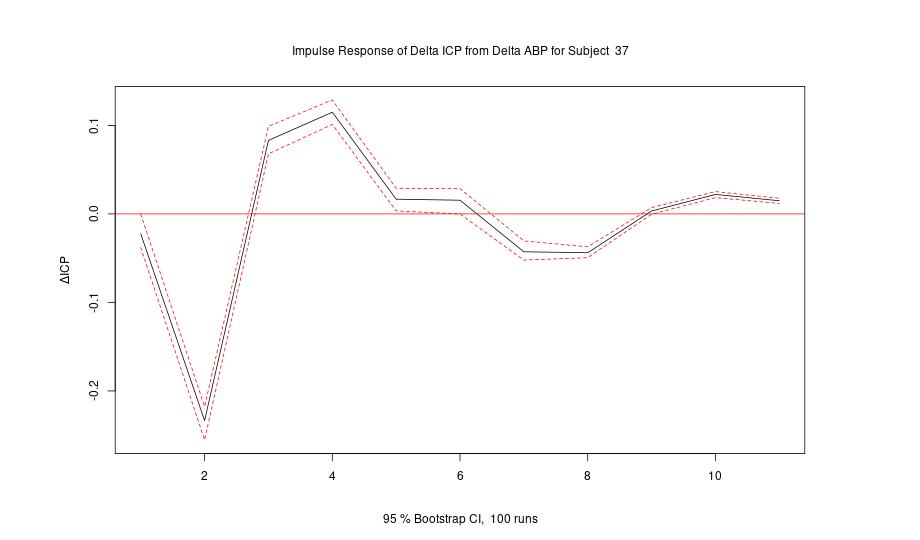

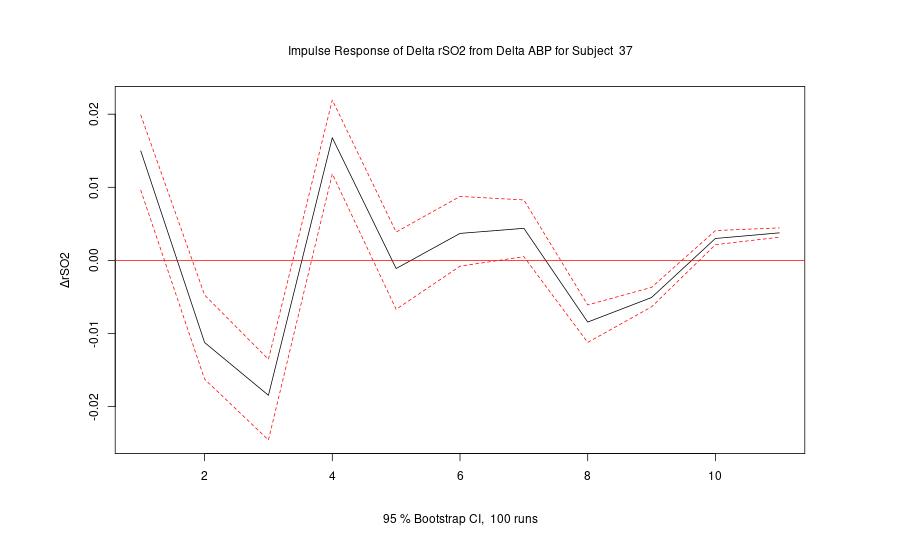


**Figure S37:** *Top panel shows the modeled resulting response in change in intracranial pressure (ΔICP) to an orthogonal impulse in change in arterial blood pressure (ΔABP). Bottom panel shows the modeled resulting response in change in regional cerebral oxygen saturation (ΔrSO_2_) to an orthogonal impulse in change in arterial blood pressure (ΔABP). The 95% confidence intervals are indicated by the red dashed line. Note the similar pattern of phases.*


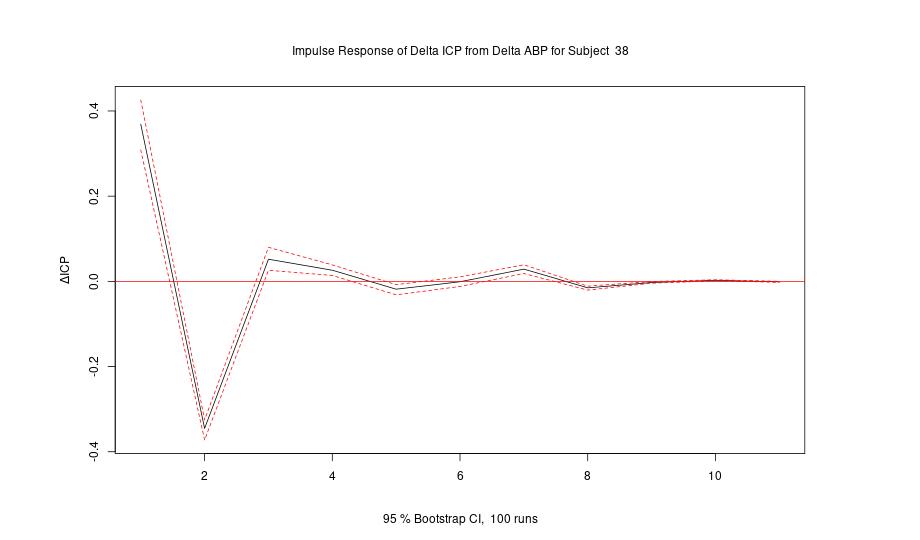

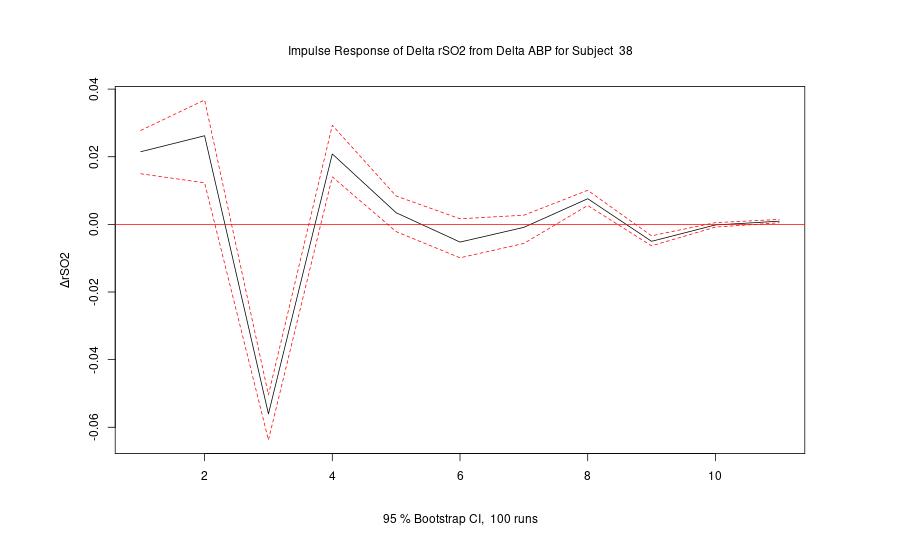


**Figure S38:** *Top panel shows the modeled resulting response in change in intracranial pressure (ΔICP) to an orthogonal impulse in change in arterial blood pressure (ΔABP). Bottom panel shows the modeled resulting response in change in regional cerebral oxygen saturation (ΔrSO_2_) to an orthogonal impulse in change in arterial blood pressure (ΔABP). The 95% confidence intervals are indicated by the red dashed line. Note the similar pattern of phases.*


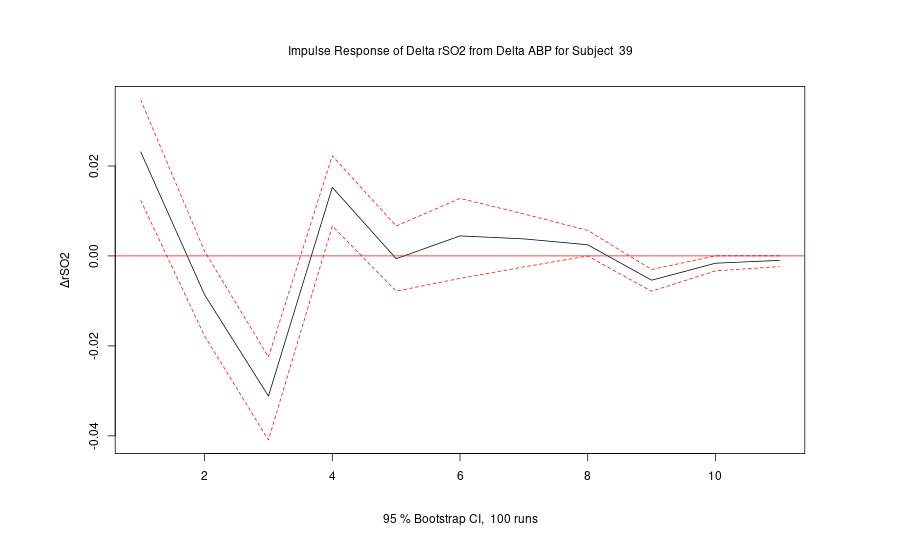

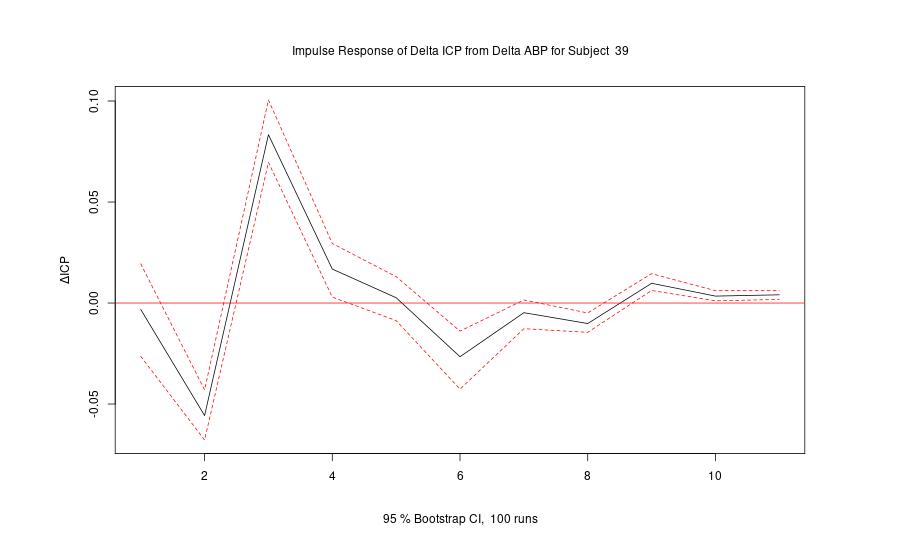


**Figure S39:** *Top panel shows the modeled resulting response in change in intracranial pressure (ΔICP) to an orthogonal impulse in change in arterial blood pressure (ΔABP). Bottom panel shows the modeled resulting response in change in regional cerebral oxygen saturation (ΔrSO_2_) to an orthogonal impulse in change in arterial blood pressure (ΔABP). The 95% confidence intervals are indicated by the red dashed line. Note the similar pattern of phases.*


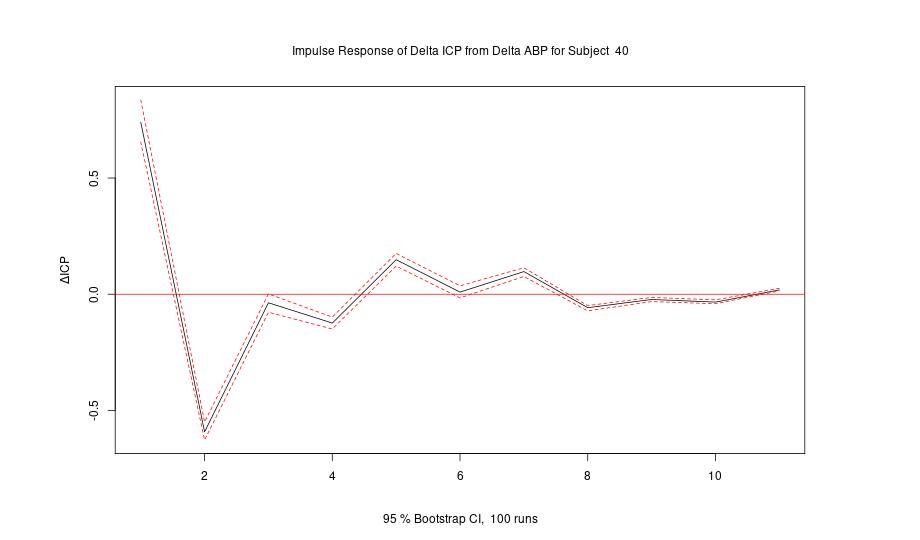

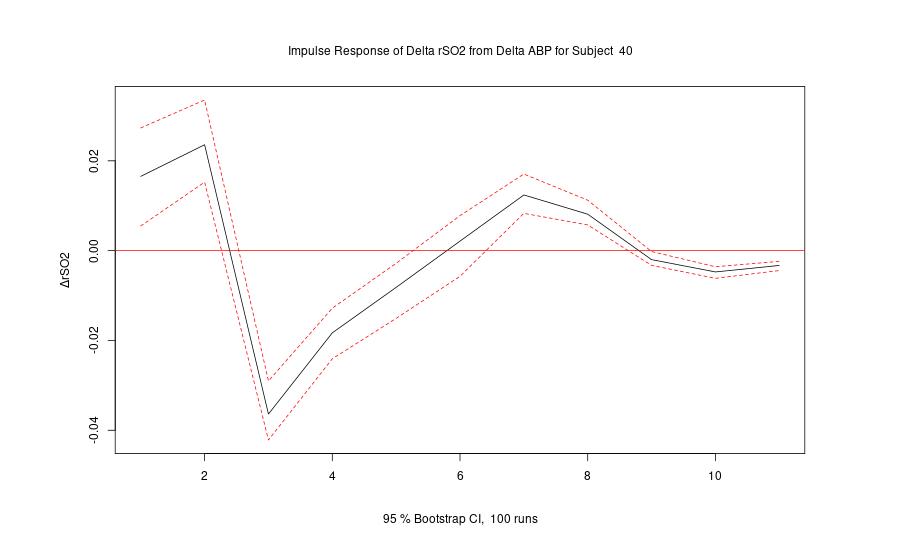


**Figure S40:** *Top panel shows the modeled resulting response in change in intracranial pressure (ΔICP) to an orthogonal impulse in change in arterial blood pressure (ΔABP). Bottom panel shows the modeled resulting response in change in regional cerebral oxygen saturation (ΔrSO_2_) to an orthogonal impulse in change in arterial blood pressure (ΔABP). The 95% confidence intervals are indicated by the red dashed line. Note the similar pattern of phases.*


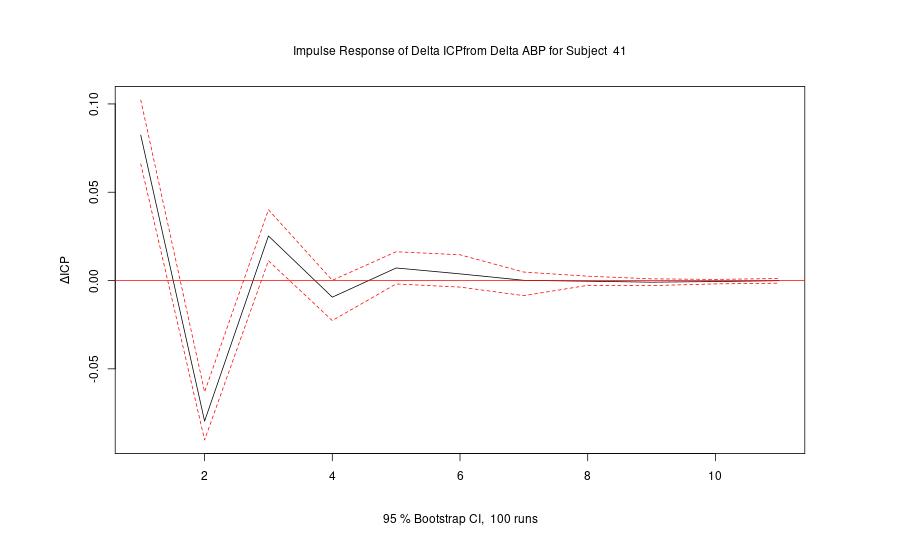

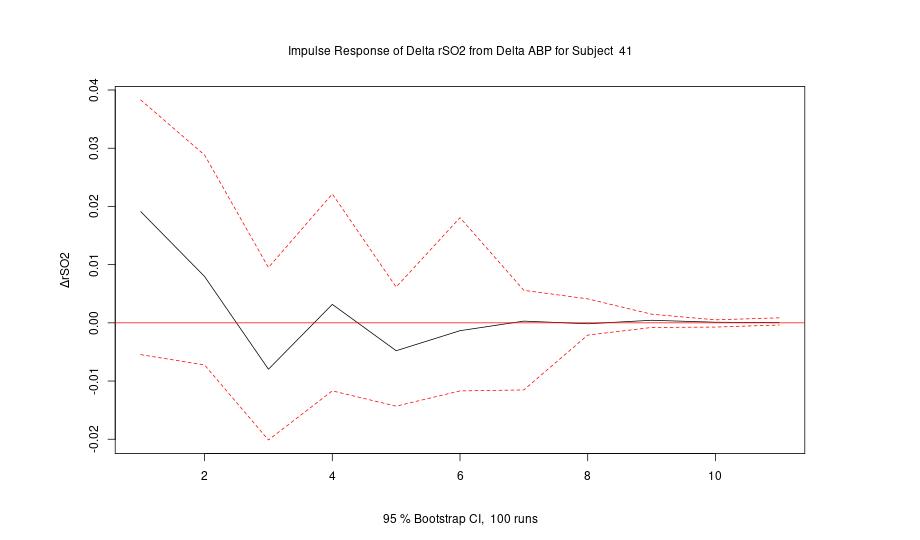


**Figure S41:** *Top panel shows the modeled resulting response in change in intracranial pressure (ΔICP) to an orthogonal impulse in change in arterial blood pressure (ΔABP). Bottom panel shows the modeled resulting response in change in regional cerebral oxygen saturation (ΔrSO_2_) to an orthogonal impulse in change in arterial blood pressure (ΔABP). The 95% confidence intervals are indicated by the red dashed line. Note the similar pattern of phases.*


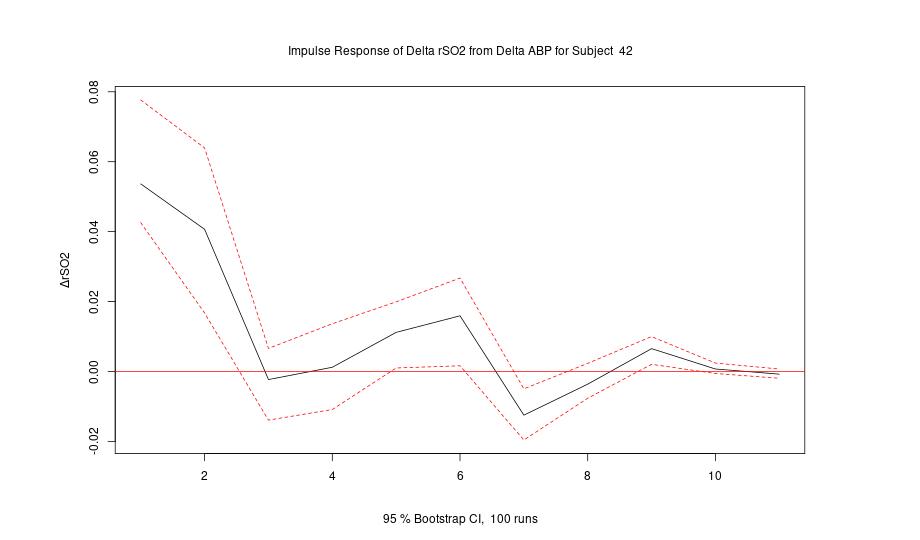

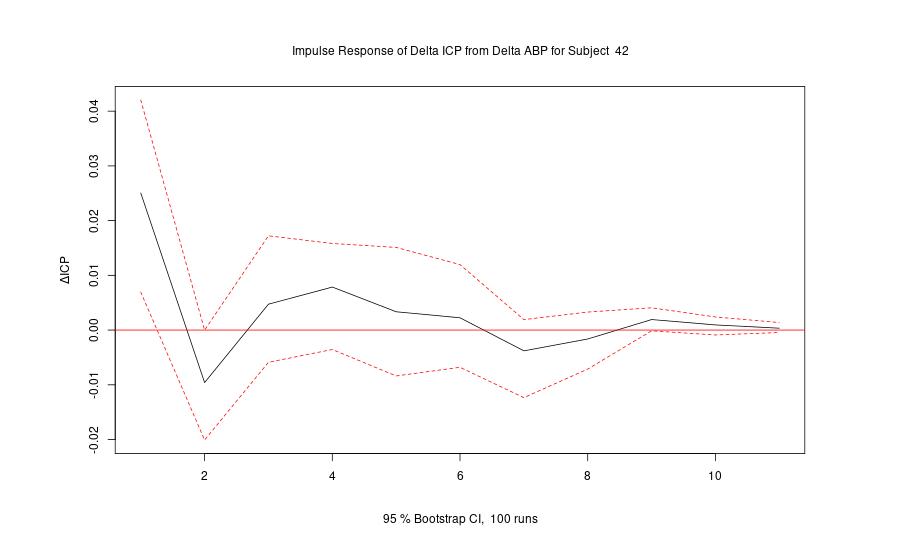


**Figure S42:** *Top panel shows the modeled resulting response in change in intracranial pressure (ΔICP) to an orthogonal impulse in change in arterial blood pressure (ΔABP). Bottom panel shows the modeled resulting response in change in regional cerebral oxygen saturation (ΔrSO_2_) to an orthogonal impulse in change in arterial blood pressure (ΔABP). The 95% confidence intervals are indicated by the red dashed line. Note the similar pattern of phases.*


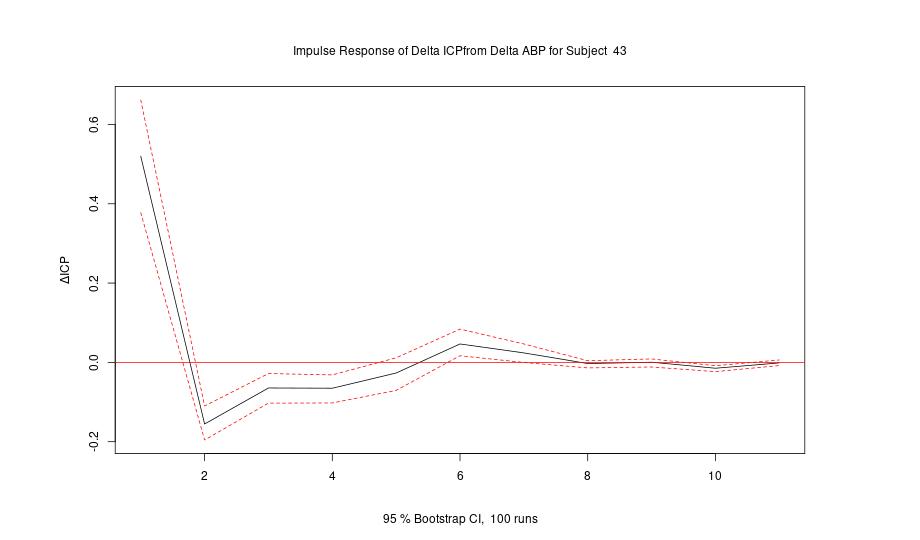

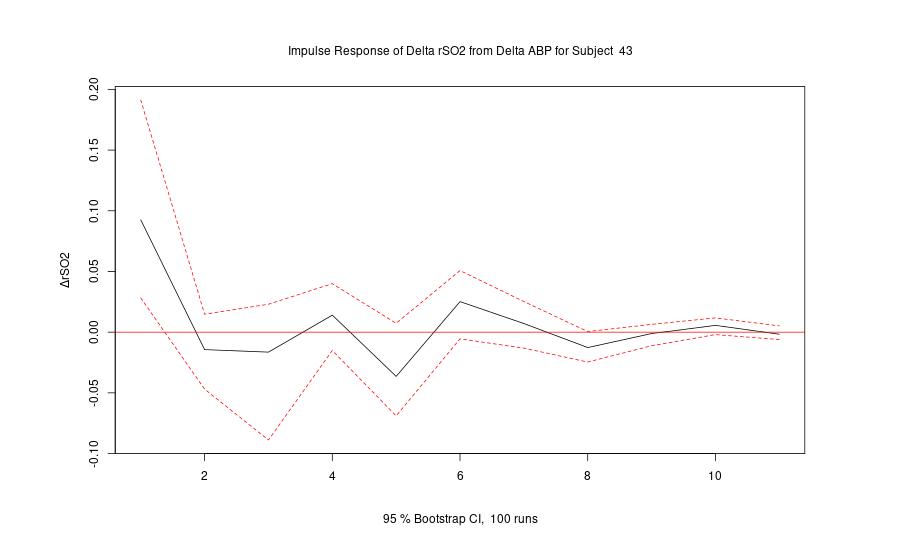


**Figure S43:** *Top panel shows the modeled resulting response in change in intracranial pressure (ΔICP) to an orthogonal impulse in change in arterial blood pressure (ΔABP). Bottom panel shows the modeled resulting response in change in regional cerebral oxygen saturation (ΔrSO_2_) to an orthogonal impulse in change in arterial blood pressure (ΔABP). The 95% confidence intervals are indicated by the red dashed line. Note the similar pattern of phases.*


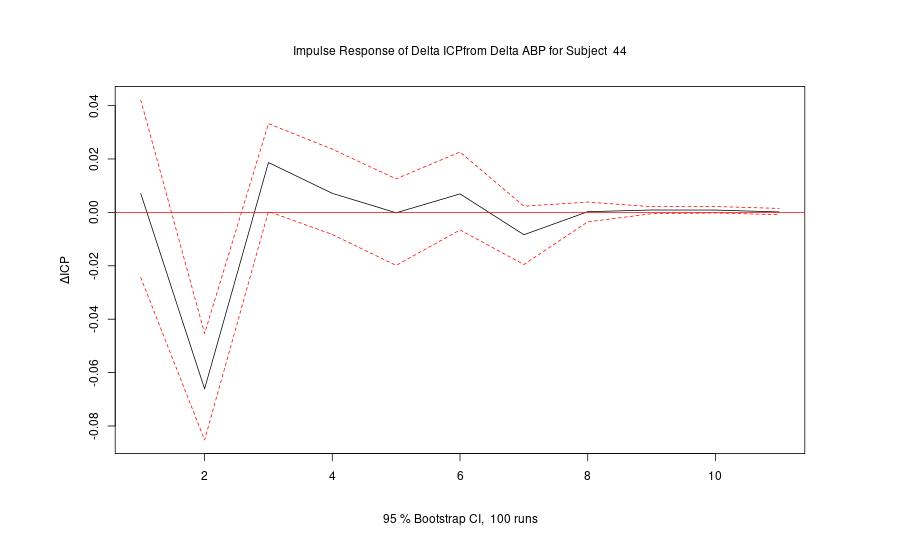

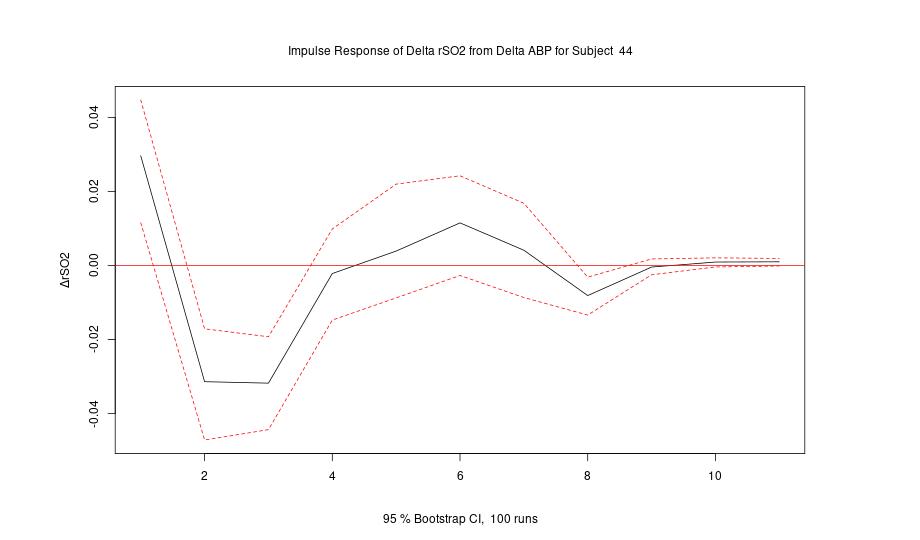


**Figure S44:** *Top panel shows the modeled resulting response in change in intracranial pressure (ΔICP) to an orthogonal impulse in change in arterial blood pressure (ΔABP). Bottom panel shows the modeled resulting response in change in regional cerebral oxygen saturation (ΔrSO_2_) to an orthogonal impulse in change in arterial blood pressure (ΔABP). The 95% confidence intervals are indicated by the red dashed line. Note the similar pattern of phases.*


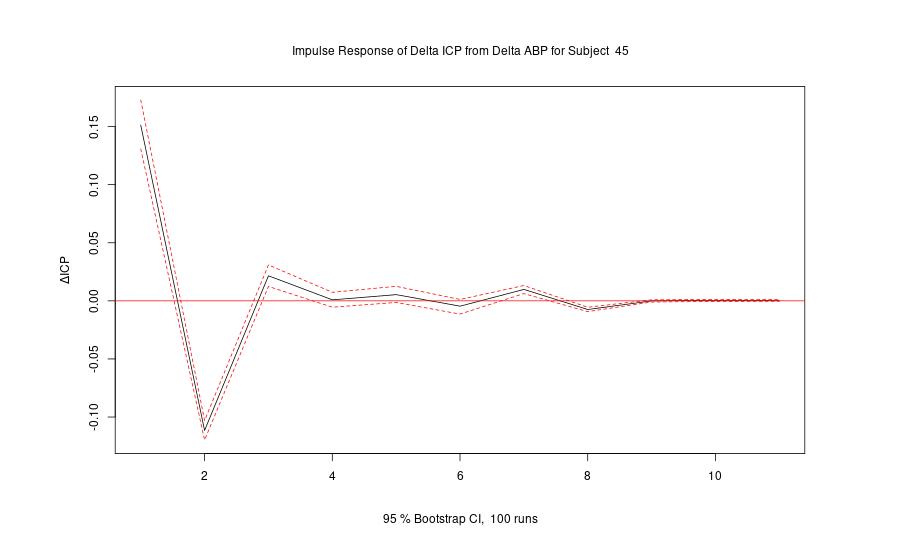

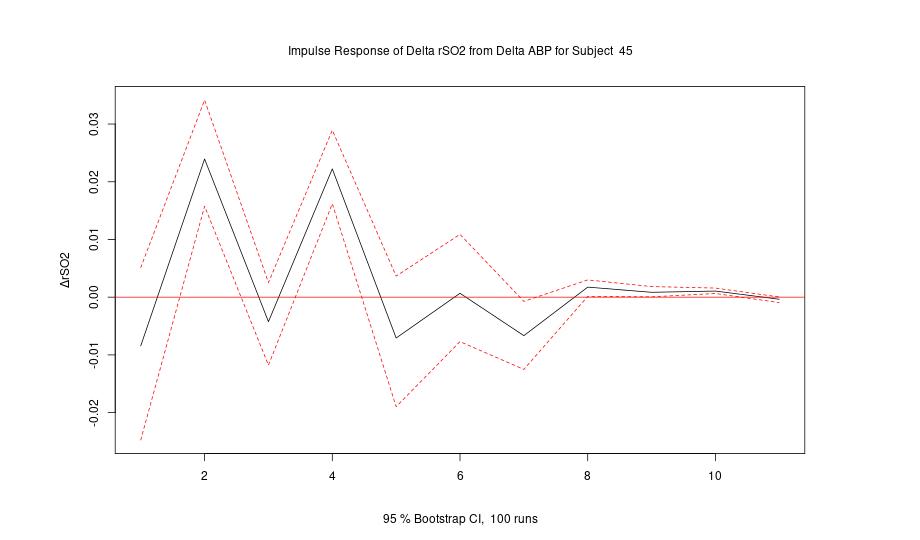


**Figure S45:** *Top panel shows the modeled resulting response in change in intracranial pressure (ΔICP) to an orthogonal impulse in change in arterial blood pressure (ΔABP). Bottom panel shows the modeled resulting response in change in regional cerebral oxygen saturation (ΔrSO_2_) to an orthogonal impulse in change in arterial blood pressure (ΔABP). The 95% confidence intervals are indicated by the red dashed line. Note the dissimilar pattern of phases.*


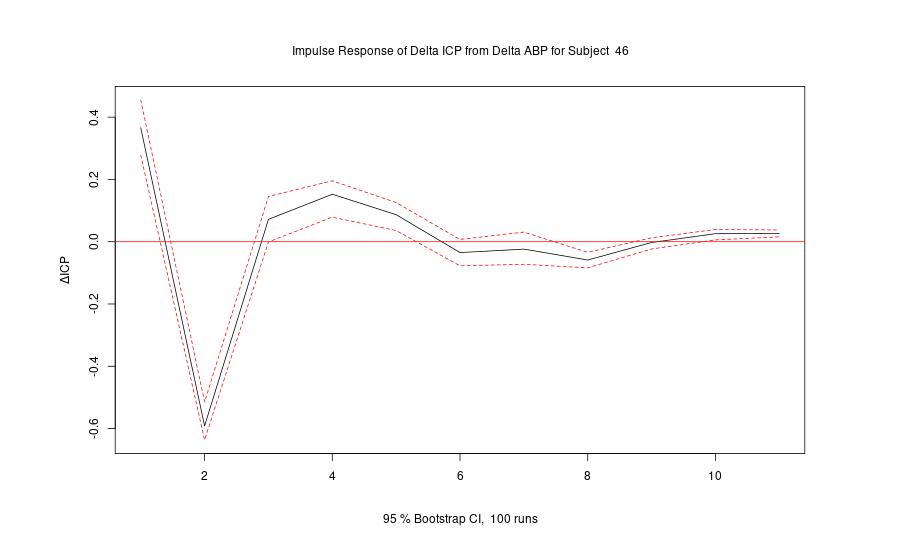

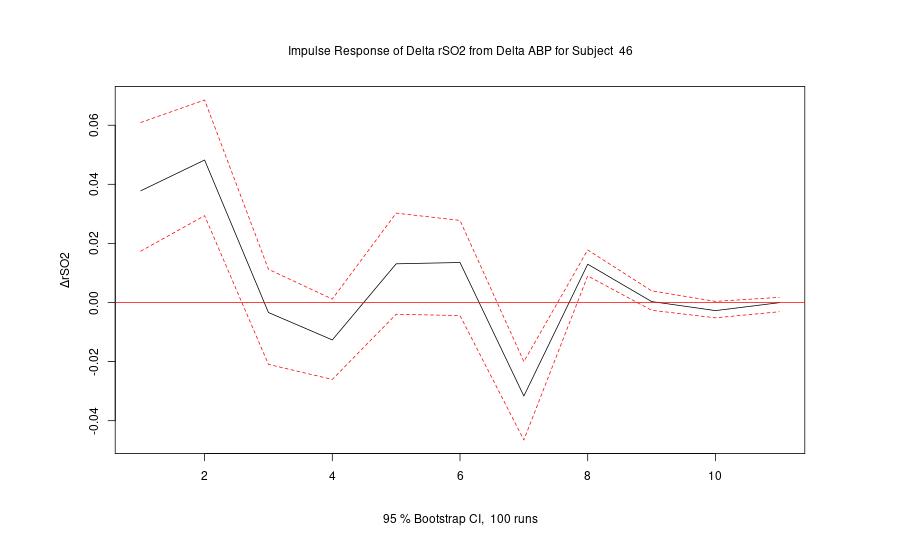


**Figure S46:** *Top panel shows the modeled resulting response in change in intracranial pressure (ΔICP) to an orthogonal impulse in change in arterial blood pressure (ΔABP). Bottom panel shows the modeled resulting response in change in regional cerebral oxygen saturation (ΔrSO_2_) to an orthogonal impulse in change in arterial blood pressure (ΔABP). The 95% confidence intervals are indicated by the red dashed line. Note the dissimilar pattern of phases.*


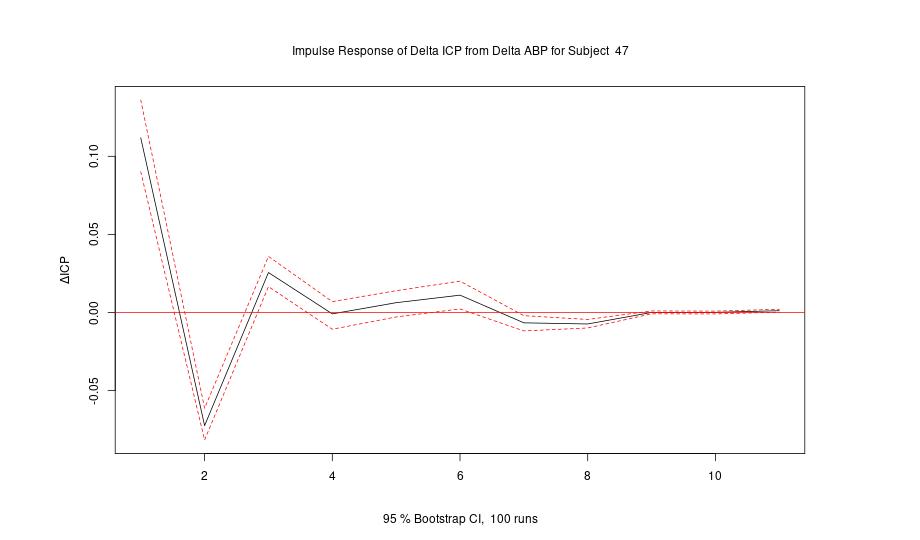

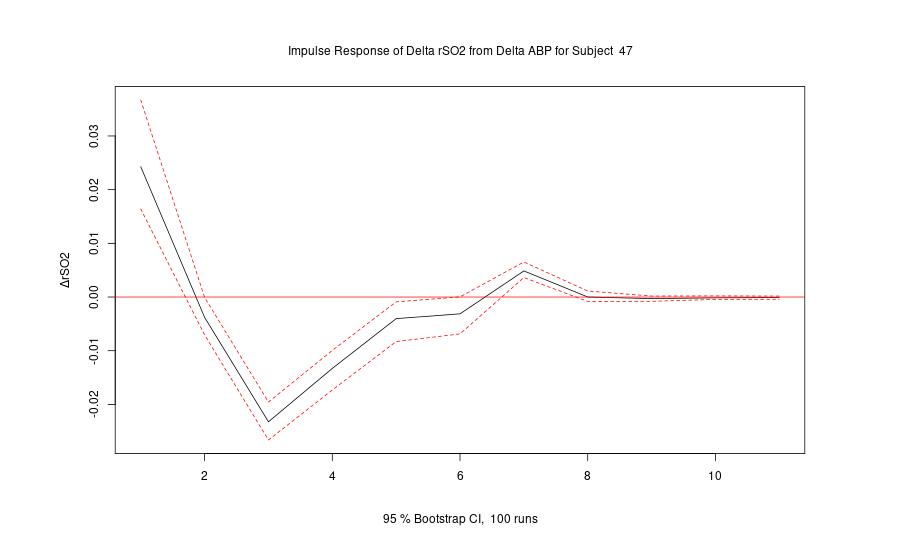


**Figure S47:** *Top panel shows the modeled resulting response in change in intracranial pressure (ΔICP) to an orthogonal impulse in change in arterial blood pressure (ΔABP). Bottom panel shows the modeled resulting response in change in regional cerebral oxygen saturation (ΔrSO_2_) to an orthogonal impulse in change in arterial blood pressure (ΔABP). The 95% confidence intervals are indicated by the red dashed line. Note the similar pattern of phases.*


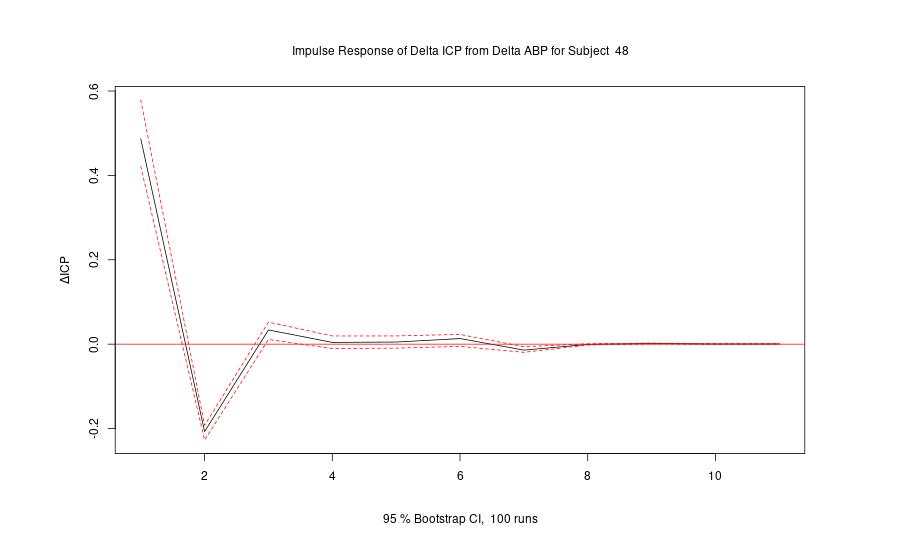

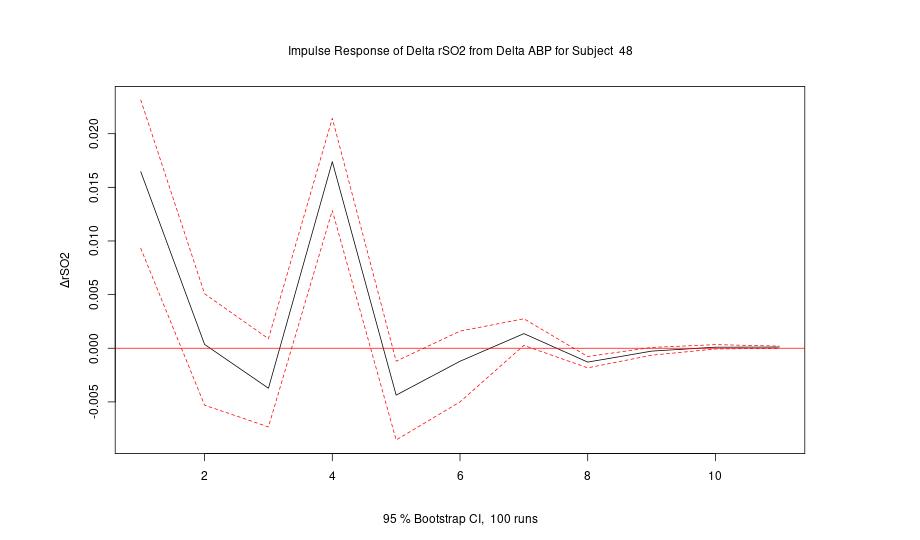


**Figure S48:** *Top panel shows the modeled resulting response in change in intracranial pressure (ΔICP) to an orthogonal impulse in change in arterial blood pressure (ΔABP). Bottom panel shows the modeled resulting response in change in regional cerebral oxygen saturation (ΔrSO_2_) to an orthogonal impulse in change in arterial blood pressure (ΔABP). The 95% confidence intervals are indicated by the red dashed line. Note the similar pattern of phases.*


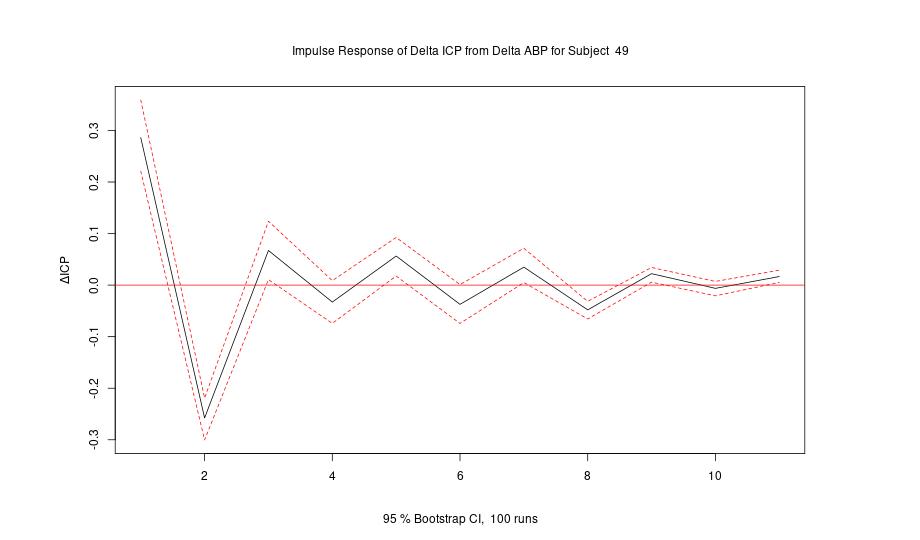

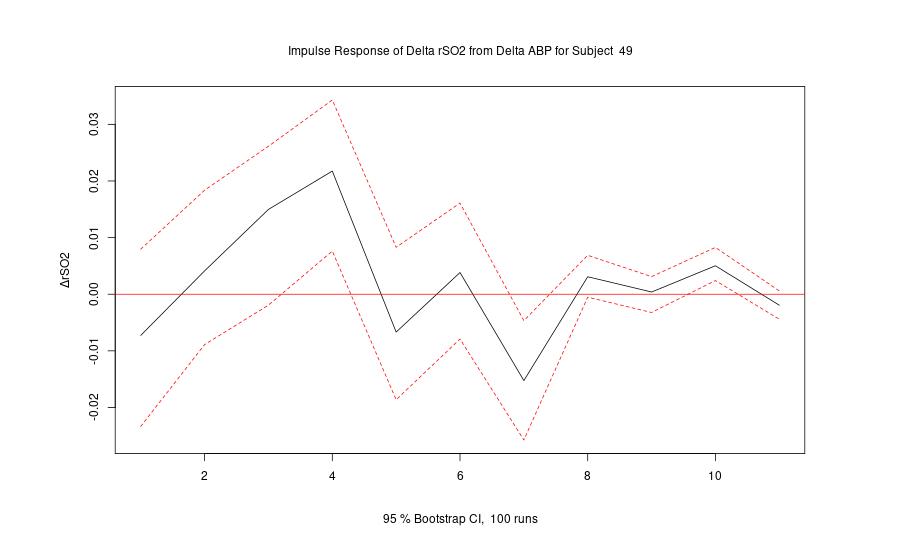


**Figure S49:** *Top panel shows the modeled resulting response in change in intracranial pressure (ΔICP) to an orthogonal impulse in change in arterial blood pressure (ΔABP). Bottom panel shows the modeled resulting response in change in regional cerebral oxygen saturation (ΔrSO_2_) to an orthogonal impulse in change in arterial blood pressure (ΔABP). The 95% confidence intervals are indicated by the red dashed line. Note the dissimilar pattern of phases.*


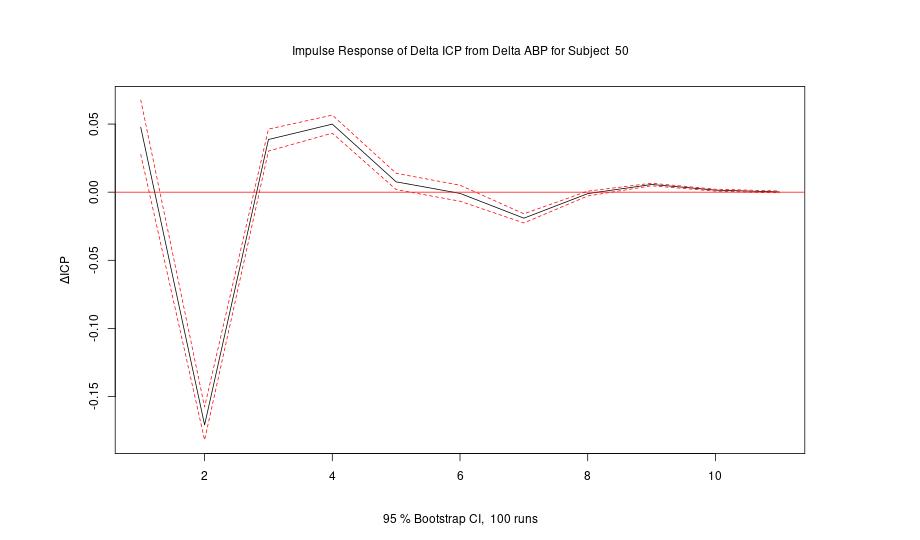

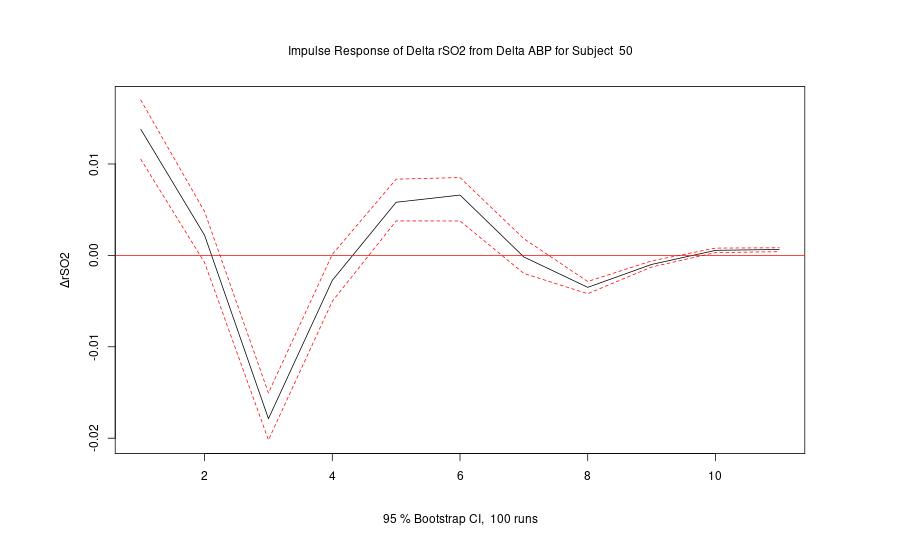


**Figure S50:** *Top panel shows the modeled resulting response in change in intracranial pressure (ΔICP) to an orthogonal impulse in change in arterial blood pressure (ΔABP). Bottom panel shows the modeled resulting response in change in regional cerebral oxygen saturation (ΔrSO_2_) to an orthogonal impulse in change in arterial blood pressure (ΔABP). The 95% confidence intervals are indicated by the red dashed line. Note the similar pattern of phases.*

**Figure S51:** *Top panel shows the modeled resulting response in change in intracranial pressure (ΔICP) to an orthogonal impulse in change in arterial blood pressure (ΔABP). Bottom panel shows the modeled resulting response in change in regional cerebral oxygen saturation (ΔrSO_2_) to an orthogonal impulse in change in arterial blood pressure (ΔABP). The 95% confidence intervals are indicated by the red dashed line. Note the dissimilar pattern of phases.*

**Figure S52:** *Top panel shows the modeled resulting response in change in intracranial pressure (ΔICP) to an orthogonal impulse in change in arterial blood pressure (ΔABP). Bottom panel shows the modeled resulting response in change in regional cerebral oxygen saturation (ΔrSO_2_) to an orthogonal impulse in change in arterial blood pressure (ΔABP). The 95% confidence intervals are indicated by the red dashed line. Note the dissimilar pattern of phases.*

**Figure S53:** *Top panel shows the modeled resulting response in change in intracranial pressure (ΔICP) to an orthogonal impulse in change in arterial blood pressure (ΔABP). Bottom panel shows the modeled resulting response in change in regional cerebral oxygen saturation (ΔrSO_2_) to an orthogonal impulse in change in arterial blood pressure (ΔABP). The 95% confidence intervals are indicated by the red dashed line. Note the similar pattern of phases.*

**Figure S54:** *Top panel shows the modeled resulting response in change in intracranial pressure (ΔICP) to an orthogonal impulse in change in arterial blood pressure (ΔABP). Bottom panel shows the modeled resulting response in change in regional cerebral oxygen saturation (ΔrSO_2_) to an orthogonal impulse in change in arterial blood pressure (ΔABP). The 95% confidence intervals are indicated by the red dashed line. Note the dissimilar pattern of phases.*

**Figure S55:** *Top panel shows the modeled resulting response in change in intracranial pressure (ΔICP) to an orthogonal impulse in change in arterial blood pressure (ΔABP). Bottom panel shows the modeled resulting response in change in regional cerebral oxygen saturation (ΔrSO_2_) to an orthogonal impulse in change in arterial blood pressure (ΔABP). The 95% confidence intervals are indicated by the red dashed line. Note the similar pattern of phases.*

**Figure S56:** *Top panel shows the modeled resulting response in change in intracranial pressure (ΔICP) to an orthogonal impulse in change in arterial blood pressure (ΔABP). Bottom panel shows the modeled resulting response in change in regional cerebral oxygen saturation (ΔrSO_2_) to an orthogonal impulse in change in arterial blood pressure (ΔABP). The 95% confidence intervals are indicated by the red dashed line. Note the dissimilar pattern of phases.*

**Figure S57:** *Top panel shows the modeled resulting response in change in intracranial pressure (ΔICP) to an orthogonal impulse in change in arterial blood pressure (ΔABP). Bottom panel shows the modeled resulting response in change in regional cerebral oxygen saturation (ΔrSO_2_) to an orthogonal impulse in change in arterial blood pressure (ΔABP). The 95% confidence intervals are indicated by the red dashed line. Note the similar pattern of phases.*

**Figure S58:** *Top panel shows the modeled resulting response in change in intracranial pressure (ΔICP) to an orthogonal impulse in change in arterial blood pressure (ΔABP). Bottom panel shows the modeled resulting response in change in regional cerebral oxygen saturation (ΔrSO_2_) to an orthogonal impulse in change in arterial blood pressure (ΔABP). The 95% confidence intervals are indicated by the red dashed line. Note the similar pattern of phases.*

**Figure S59:** *Top panel shows the modeled resulting response in change in intracranial pressure (ΔICP) to an orthogonal impulse in change in arterial blood pressure (ΔABP). Bottom panel shows the modeled resulting response in change in regional cerebral oxygen saturation (ΔrSO_2_) to an orthogonal impulse in change in arterial blood pressure (ΔABP). The 95% confidence intervals are indicated by the red dashed line. Note the dissimilar pattern of phases.*

**Figure S60:** *Top panel shows the modeled resulting response in change in intracranial pressure (ΔICP) to an orthogonal impulse in change in arterial blood pressure (ΔABP). Bottom panel shows the modeled resulting response in change in regional cerebral oxygen saturation (ΔrSO_2_) to an orthogonal impulse in change in arterial blood pressure (ΔABP). The 95% confidence intervals are indicated by the red dashed line. Note the similar pattern of phases.*

**Figure S61:** *Top panel shows the modeled resulting response in change in intracranial pressure (ΔICP) to an orthogonal impulse in change in arterial blood pressure (ΔABP). Bottom panel shows the modeled resulting response in change in regional cerebral oxygen saturation (ΔrSO_2_) to an orthogonal impulse in change in arterial blood pressure (ΔABP). The 95% confidence intervals are indicated by the red dashed line. Note the similar pattern of phases.*

**Figure S62:** *Top panel shows the modeled resulting response in change in intracranial pressure (ΔICP) to an orthogonal impulse in change in arterial blood pressure (ΔABP). Bottom panel shows the modeled resulting response in change in regional cerebral oxygen saturation (ΔrSO_2_) to an orthogonal impulse in change in arterial blood pressure (ΔABP). The 95% confidence intervals are indicated by the red dashed line. Note the similar pattern of phases.*

**Figure S63:** *Top panel shows the modeled resulting response in change in intracranial pressure (ΔICP) to an orthogonal impulse in change in arterial blood pressure (ΔABP). Bottom panel shows the modeled resulting response in change in regional cerebral oxygen saturation (ΔrSO_2_) to an orthogonal impulse in change in arterial blood pressure (ΔABP). The 95% confidence intervals are indicated by the red dashed line. Note the similar pattern of phases.*

**Figure S64:** *Top panel shows the modeled resulting response in change in intracranial pressure (ΔICP) to an orthogonal impulse in change in arterial blood pressure (ΔABP). Bottom panel shows the modeled resulting response in change in regional cerebral oxygen saturation (ΔrSO_2_) to an orthogonal impulse in change in arterial blood pressure (ΔABP). The 95% confidence intervals are indicated by the red dashed line. Note the similar pattern of phases.*

**Figure S65:** *Top panel shows the modeled resulting response in change in intracranial pressure (ΔICP) to an orthogonal impulse in change in arterial blood pressure (ΔABP). Bottom panel shows the modeled resulting response in change in regional cerebral oxygen saturation (ΔrSO_2_) to an orthogonal impulse in change in arterial blood pressure (ΔABP). The 95% confidence intervals are indicated by the red dashed line. Note the similar pattern of phases.*

**Figure S66:** *Top panel shows the modeled resulting response in change in intracranial pressure (ΔICP) to an orthogonal impulse in change in arterial blood pressure (ΔABP). Bottom panel shows the modeled resulting response in change in regional cerebral oxygen saturation (ΔrSO_2_) to an orthogonal impulse in change in arterial blood pressure (ΔABP). The 95% confidence intervals are indicated by the red dashed line. Note the similar pattern of phases.*

**Figure S67:** *Top panel shows the modeled resulting response in change in intracranial pressure (ΔICP) to an orthogonal impulse in change in arterial blood pressure (ΔABP). Bottom panel shows the modeled resulting response in change in regional cerebral oxygen saturation (ΔrSO_2_) to an orthogonal impulse in change in arterial blood pressure (ΔABP). The 95% confidence intervals are indicated by the red dashed line. Note the similar pattern of phases.*

**Figure S68:** *Top panel shows the modeled resulting response in change in intracranial pressure (ΔICP) to an orthogonal impulse in change in arterial blood pressure (ΔABP). Bottom panel shows the modeled resulting response in change in regional cerebral oxygen saturation (ΔrSO_2_) to an orthogonal impulse in change in arterial blood pressure (ΔABP). The 95% confidence intervals are indicated by the red dashed line. Note the similar pattern of phases.*

**Figure S69:** *Top panel shows the modeled resulting response in change in intracranial pressure (ΔICP) to an orthogonal impulse in change in arterial blood pressure (ΔABP). Bottom panel shows the modeled resulting response in change in regional cerebral oxygen saturation (ΔrSO_2_) to an orthogonal impulse in change in arterial blood pressure (ΔABP). The 95% confidence intervals are indicated by the red dashed line. Note the similar pattern of phases.*

**Figure S70:** *Top panel shows the modeled resulting response in change in intracranial pressure (ΔICP) to an orthogonal impulse in change in arterial blood pressure (ΔABP). Bottom panel shows the modeled resulting response in change in regional cerebral oxygen saturation (ΔrSO_2_) to an orthogonal impulse in change in arterial blood pressure (ΔABP). The 95% confidence intervals are indicated by the red dashed line. Note the similar pattern of phases.*

**Figure S71:** *Top panel shows the modeled resulting response in change in intracranial pressure (ΔICP) to an orthogonal impulse in change in arterial blood pressure (ΔABP). Bottom panel shows the modeled resulting response in change in regional cerebral oxygen saturation (ΔrSO_2_) to an orthogonal impulse in change in arterial blood pressure (ΔABP). The 95% confidence intervals are indicated by the red dashed line. Note the similar pattern of phases.*

**Figure S72:** *Top panel shows the modeled resulting response in change in intracranial pressure (ΔICP) to an orthogonal impulse in change in arterial blood pressure (ΔABP). Bottom panel shows the modeled resulting response in change in regional cerebral oxygen saturation (ΔrSO_2_) to an orthogonal impulse in change in arterial blood pressure (ΔABP). The 95% confidence intervals are indicated by the red dashed line. Note the similar pattern of phases.*

**Figure S73:** *Top panel shows the modeled resulting response in change in intracranial pressure (ΔICP) to an orthogonal impulse in change in arterial blood pressure (ΔABP). Bottom panel shows the modeled resulting response in change in regional cerebral oxygen saturation (ΔrSO_2_) to an orthogonal impulse in change in arterial blood pressure (ΔABP). The 95% confidence intervals are indicated by the red dashed line. Note the similar pattern of phases.*

**Figure S74:** *Top panel shows the modeled resulting response in change in intracranial pressure (ΔICP) to an orthogonal impulse in change in arterial blood pressure (ΔABP). Bottom panel shows the modeled resulting response in change in regional cerebral oxygen saturation (ΔrSO_2_) to an orthogonal impulse in change in arterial blood pressure (ΔABP). The 95% confidence intervals are indicated by the red dashed line. Note the similar pattern of phases.*

**Figure S75:** *Top panel shows the modeled resulting response in change in intracranial pressure (ΔICP) to an orthogonal impulse in change in arterial blood pressure (ΔABP). Bottom panel shows the modeled resulting response in change in regional cerebral oxygen saturation (ΔrSO_2_) to an orthogonal impulse in change in arterial blood pressure (ΔABP). The 95% confidence intervals are indicated by the red dashed line. Note the dissimilar pattern of phases.*

**Figure S76:** *Top panel shows the modeled resulting response in change in intracranial pressure (ΔICP) to an orthogonal impulse in change in arterial blood pressure (ΔABP). Bottom panel shows the modeled resulting response in change in regional cerebral oxygen saturation (ΔrSO_2_) to an orthogonal impulse in change in arterial blood pressure (ΔABP). The 95% confidence intervals are indicated by the red dashed line. Note the similar pattern of phases.*

**Figure S77:** *Top panel shows the modeled resulting response in change in intracranial pressure (ΔICP) to an orthogonal impulse in change in arterial blood pressure (ΔABP). Bottom panel shows the modeled resulting response in change in regional cerebral oxygen saturation (ΔrSO_2_) to an orthogonal impulse in change in arterial blood pressure (ΔABP). The 95% confidence intervals are indicated by the red dashed line. Note the similar pattern of phases.*

**Figure S78:** *Top panel shows the modeled resulting response in change in intracranial pressure (ΔICP) to an orthogonal impulse in change in arterial blood pressure (ΔABP). Bottom panel shows the modeled resulting response in change in regional cerebral oxygen saturation (ΔrSO_2_) to an orthogonal impulse in change in arterial blood pressure (ΔABP). The 95% confidence intervals are indicated by the red dashed line. Note the similar pattern of phases.*

**Figure S79:** *Top panel shows the modeled resulting response in change in intracranial pressure (ΔICP) to an orthogonal impulse in change in arterial blood pressure (ΔABP). Bottom panel shows the modeled resulting response in change in regional cerebral oxygen saturation (ΔrSO_2_) to an orthogonal impulse in change in arterial blood pressure (ΔABP). The 95% confidence intervals are indicated by the red dashed line. Note the dissimilar pattern of phases.*

**Figure S80:** *Top panel shows the modeled resulting response in change in intracranial pressure (ΔICP) to an orthogonal impulse in change in arterial blood pressure (ΔABP). Bottom panel shows the modeled resulting response in change in regional cerebral oxygen saturation (ΔrSO_2_) to an orthogonal impulse in change in arterial blood pressure (ΔABP). The 95% confidence intervals are indicated by the red dashed line. Note the similar pattern of phases.*

**Figure S81:** *Top panel shows the modeled resulting response in change in intracranial pressure (ΔICP) to an orthogonal impulse in change in arterial blood pressure (ΔABP). Bottom panel shows the modeled resulting response in change in regional cerebral oxygen saturation (ΔrSO_2_) to an orthogonal impulse in change in arterial blood pressure (ΔABP). The 95% confidence intervals are indicated by the red dashed line. Note the similar pattern of phases.*

**Figure S82:** *Top panel shows the modeled resulting response in change in intracranial pressure (ΔICP) to an orthogonal impulse in change in arterial blood pressure (ΔABP). Bottom panel shows the modeled resulting response in change in regional cerebral oxygen saturation (ΔrSO_2_) to an orthogonal impulse in change in arterial blood pressure (ΔABP). The 95% confidence intervals are indicated by the red dashed line. Note the similar pattern of phases.*

**Figure S83:** *Top panel shows the modeled resulting response in change in intracranial pressure (ΔICP) to an orthogonal impulse in change in arterial blood pressure (ΔABP). Bottom panel shows the modeled resulting response in change in regional cerebral oxygen saturation (ΔrSO_2_) to an orthogonal impulse in change in arterial blood pressure (ΔABP). The 95% confidence intervals are indicated by the red dashed line. Note the similar pattern of phases.*

**Table S1:** Here is found the number of 10 second datapoints for each subject in the study.

| **Subject Number** | **Number of 10-Second Datapoints** |
| --- | --- |
| 1 | 22090 |
| 2 | 4989 |
| 3 | 3760 |
| 4 | 5643 |
| 5 | 63180 |
| 6 | 1817 |
| 7 | 4634 |
| 8 | 12813 |
| 9 | 1879 |
| 10 | 12801 |
| 11 | 25134 |
| 12 | 18752 |
| 13 | 872 |
| 14 | 11418 |
| 15 | 5729 |
| 16 | 6012 |
| 17 | 39870 |
| 18 | 31800 |
| 19 | 33635 |
| 20 | 13887 |
| 21 | 26394 |
| 22 | 27153 |
| 23 | 16923 |
| 24 | 13390 |
| 25 | 9596 |
| 26 | 21316 |
| 27 | 8312 |
| 28 | 12647 |
| 29 | 25451 |
| 30 | 7132 |
| 31 | 8618 |
| 32 | 6926 |
| 33 | 23058 |
| 34 | 4359 |
| 35 | 19261 |
| 36 | 566 |
| 37 | 74101 |
| 38 | 23184 |
| 39 | 7312 |
| 40 | 14921 |
| 41 | 6230 |
| 42 | 5023 |
| 43 | 4101 |
| 44 | 6719 |
| 45 | 28244 |
| 46 | 6045 |
| 47 | 23824 |
| 48 | 47846 |
| 49 | 2267 |
| 50 | 82806 |
| 51 | 6953 |
| 52 | 18444 |
| 53 | 47274 |
| 54 | 4378 |
| 55 | 7743 |
| 56 | 15346 |
| 57 | 43484 |
| 58 | 58319 |
| 59 | 4654 |
| 60 | 12573 |
| 61 | 16486 |
| 62 | 47634 |
| 63 | 54891 |
| 64 | 56082 |
| 65 | 73860 |
| 66 | 40731 |
| 67 | 29546 |
| 68 | 67528 |
| 69 | 46201 |
| 70 | 49652 |
| 71 | 6347 |
| 72 | 33404 |
| 73 | 113 |
| 74 | 16329 |
| 75 | 25370 |
| 76 | 20508 |
| 77 | 56336 |
| 78 | 7189 |
| 79 | 18084 |
| 80 | 72670 |
| 81 | 22845 |
| 82 | 21416 |
| 83 | 30795 |
