# Supplementary material for: Statistical properties of cerebral near infrared and intracranial pressure-based cerebrovascular reactivity metrics in moderate and severe neural injury: a machine learning and time-series analysis
Source: Intensive Care Med Exp. 2023 Aug 28;11:57. doi: 10.1186/s40635-023-00541-3 (PMC10460757; doi:10.1186/s40635-023-00541-3)
Supplement: Supplementary file 4 — Additional file 4: The results of Granger causality testing for individual subjects. [file 40635_2023_541_MOESM4_ESM.docx]

**Additional File 4**

**Table S1: The results of Granger Causality testing for individual subjects.**

| **Subject #** | **ΔABP → ΔrSO_2_**  **(F Statistic)** | **ΔABP → ΔrSO_2_**  **(p-value)** | **ΔABP → ΔICP**  **(F Statistic)** | **ΔABP → ΔICP**  **(p-value)** | **ΔrSO_2_ → ΔABP**  **(F Statistic)** | **ΔrSO_2_ → ΔABP**  **(p-value)** | **ΔICP → ΔABP**  **(F Statistic)** | **ΔICP → ΔABP**  **(p-value)** |
| --- | --- | --- | --- | --- | --- | --- | --- | --- |
| **1** | 10.94 | >0.01 | 123.26 | >0.01 | 2.53 | 0.11 | 209.22 | >0.01 |
| **2** | 100.60 | >0.01 | 1140.22 | >0.01 | 1.88 | 0.17 | 770.40 | >0.01 |
| **3** | 53.80 | >0.01 | 985.68 | >0.01 | 27.15 | >0.01 | 140.10 | >0.01 |
| **4** | 64.70 | >0.01 | 364.58 | >0.01 | 1.13 | 0.29 | 0.02 | 0.88 |
| **5** | 927.17 | >0.01 | 6961.63 | >0.01 | 15.83 | >0.01 | 536.07 | >0.01 |
| **6** | 39.80 | >0.01 | 52.20 | >0.01 | 1.16 | 0.28 | 20.27 | >0.01 |
| **7** | 0.15 | 0.70 | 58.04 | >0.01 | 2.39 | 0.12 | 0.03 | 0.86 |
| **8** | 983.17 | >0.01 | 3078.96 | >0.01 | 29.62 | >0.01 | 182.32 | >0.01 |
| **9** | 2.63 | 0.10 | 86.27 | >0.01 | 0.01 | 0.91 | 1.46 | 0.23 |
| **10** | 20.41 | >0.01 | 316.38 | >0.01 | 10.92 | >0.01 | 106.57 | >0.01 |
| **11** | 354.26 | >0.01 | 1194.41 | >0.01 | 6.87 | 0.01 | 0.22 | 0.64 |
| **12** | 323.88 | >0.01 | 1984.92 | >0.01 | 14.05 | >0.01 | 427.79 | >0.01 |
| **13** | 14.26 | >0.01 | 18.64 | >0.01 | 0.62 | 0.43 | 0.35 | 0.56 |
| **14** | 18.39 | >0.01 | 155.39 | >0.01 | 3.90 | 0.05 | 1.62 | 0.20 |
| **15** | 0.07 | 0.79 | 121.42 | >0.01 | 1.24 | 0.27 | 14.08 | >0.01 |
| **16** | 14.08 | >0.01 | 120.07 | >0.01 | 2.21 | 0.14 | 3.91 | 0.05 |
| **17** | 2159.54 | >0.01 | 8292.32 | >0.01 | 264.05 | >0.01 | 4102.39 | >0.01 |
| **18** | 201.16 | >0.01 | 3814.11 | >0.01 | 0.02 | 0.89 | 997.44 | >0.01 |
| **19** | 94.41 | >0.01 | 2985.74 | >0.01 | 48.29 | >0.01 | 547.10 | >0.01 |
| **20** | 11.14 | >0.01 | 2825.56 | >0.01 | 1.08 | 0.30 | 597.07 | >0.01 |
| **21** | 7.98 | >0.01 | 2927.79 | >0.01 | 0.23 | 0.63 | 93.91 | >0.01 |
| **22** | 348.09 | >0.01 | 5405.03 | >0.01 | 235.24 | >0.01 | 2005.68 | >0.01 |
| **23** | 20.85 | >0.01 | 668.70 | >0.01 | 16.86 | >0.01 | 1089.47 | >0.01 |
| **24** | 452.95 | >0.01 | 5045.28 | >0.01 | 293.63 | >0.01 | 1893.41 | >0.01 |
| **25** | 47.65 | >0.01 | 1508.50 | >0.01 | 0.38 | 0.54 | 450.68 | >0.01 |
| **26** | 5.38 | >0.01 | 2728.97 | >0.01 | 33.97 | >0.01 | 863.98 | >0.01 |
| **27** | 148.56 | >0.01 | 28.70 | >0.01 | 0.72 | 0.40 | 22.56 | >0.01 |
| **28** | 70.73 | >0.01 | 908.66 | >0.01 | 29.44 | >0.01 | 300.02 | >0.01 |
| **29** | 49.78 | >0.01 | 4515.25 | >0.01 | 4.31 | 0.04 | 1190.98 | >0.01 |
| **30** | 40.81 | >0.01 | 1699.43 | >0.01 | 0.02 | 0.89 | 147.01 | >0.01 |
| **31** | 133.05 | >0.01 | 2703.71 | >0.01 | 46.43 | >0.01 | 729.54 | >0.01 |
| **32** | 1.26 | 0.26 | 405.91 | >0.01 | 1.10 | 0.29 | 130.38 | >0.01 |
| **33** | 80.09 | >0.01 | 2463.13 | >0.01 | 7.97 | >0.01 | 1271.27 | >0.01 |
| **34** | 13.64 | >0.01 | 598.12 | >0.01 | 17.73 | >0.01 | 70.35 | >0.01 |
| **35** | 477.43 | >0.01 | 1270.86 | >0.01 | 0.52 | 0.47 | 172.95 | >0.01 |
| **36** | 0.80 | 0.37 | 20.90 | >0.01 | 0.02 | 0.88 | 0.02 | 0.87 |
| **37** | 0.29 | 0.59 | 1737.01 | >0.01 | 19.69 | >0.01 | 80.41 | >0.01 |
| **38** | 339.07 | >0.01 | 1558.71 | >0.01 | 2.05 | 0.15 | 51.43 | >0.01 |
| **39** | 5.43 | >0.01 | 197.05 | >0.01 | 2.24 | 0.13 | 140.71 | >0.01 |
| **40** | 144.39 | >0.01 | 1517.99 | >0.01 | 8.04 | >0.01 | 468.53 | >0.01 |
| **41** | 0.10 | 0.75 | 92.68 | >0.01 | 0.31 | 0.58 | 12.88 | >0.01 |
| **42** | 75.26 | >0.01 | 8.57 | >0.01 | 0.31 | 0.58 | 39.17 | >0.01 |
| **43** | 1.05 | 0.30 | 2.39 | 0.12 | 5.88 | 0.02 | 104.66 | >0.01 |
| **44** | 0.89 | 0.35 | 46.23 | >0.01 | 11.90 | >0.01 | 17.70 | >0.01 |
| **45** | 18.93 | >0.01 | 788.66 | >0.01 | 0.06 | 0.80 | 220.10 | >0.01 |
| **46** | 35.63 | >0.01 | 623.28 | >0.01 | 0.01 | 0.94 | 140.78 | >0.01 |
| **47** | 2.66 | 0.10 | 192.77 | >0.01 | 15.52 | >0.01 | 39.98 | >0.01 |
| **48** | 0.21 | 0.65 | 389.65 | >0.01 | 0.83 | 0.36 | 686.23 | >0.01 |
| **49** | 0.51 | 0.48 | 59.27 | >0.01 | 2.28 | 0.13 | >0.01 | 0.97 |
| **50** | 33.53 | >0.01 | 2848.00 | >0.01 | 29.13 | >0.01 | 435.85 | >0.01 |
| **51** | 20.95 | >0.01 | 74.57 | >0.01 | 2.08 | 0.15 | 1.49 | 0.22 |
| **52** | 175.87 | >0.01 | 570.77 | >0.01 | 1.19 | 0.28 | 140.14 | >0.01 |
| **53** | 582.96 | >0.01 | 6429.51 | >0.01 | 49.90 | >0.01 | 680.25 | >0.01 |
| **54** | 0.09 | 0.76 | 667.39 | >0.01 | 1.93 | 0.16 | 118.44 | >0.01 |
| **55** | 0.38 | 0.54 | 157.68 | >0.01 | 0.01 | 0.91 | 119.64 | >0.01 |
| **56** | 0.23 | 0.63 | 3938.28 | >0.01 | 4.95 | 0.03 | 570.86 | >0.01 |
| **57** | 29.34 | >0.01 | 58.15 | >0.01 | 17.90 | >0.01 | 0.48 | 0.49 |
| **58** | 721.14 | >0.01 | 2291.04 | >0.01 | 0.22 | 0.64 | 5.23 | 0.02 |
| **59** | 49.80 | >0.01 | 42.61 | >0.01 | 9.60 | >0.01 | 54.50 | >0.01 |
| **60** | 2.28 | 0.13 | 120.25 | >0.01 | 1.74 | 0.19 | 55.81 | >0.01 |
| **61** | 2.34 | 0.13 | 188.14 | >0.01 | 4.39 | 0.04 | 22.60 | >0.01 |
| **62** | 4.56 | 0.03 | 2049.26 | >0.01 | 0.46 | 0.50 | 0.84 | 0.36 |
| **63** | 655.11 | >0.01 | 7006.25 | >0.01 | 190.01 | >0.01 | 988.91 | >0.01 |
| **64** | 221.73 | >0.01 | 3474.27 | >0.01 | 46.17 | >0.01 | 289.03 | >0.01 |
| **65** | 84.39 | >0.01 | 3727.35 | >0.01 | 781.39 | >0.01 | 1640.47 | >0.01 |
| **66** | 392.12 | >0.01 | 675.14 | >0.01 | 5.57 | 0.02 | 115.81 | >0.01 |
| **67** | 11.24 | >0.01 | 4165.21 | >0.01 | 11.10 | >0.01 | 644.09 | >0.01 |
| **68** | 482.52 | >0.01 | 2447.10 | >0.01 | 28.30 | >0.01 | 51.35 | >0.01 |
| **69** | 1648.31 | >0.01 | 5881.73 | >0.01 | 156.19 | >0.01 | 3045.57 | >0.01 |
| **70** | 292.85 | >0.01 | 2665.58 | >0.01 | 4.45 | 0.03 | 1441.55 | >0.01 |
| **71** | 23.00 | >0.01 | 84.27 | >0.01 | 0.77 | 0.38 | 4.18 | 0.04 |
| **72** | 90.38 | >0.01 | 2671.94 | >0.01 | 1.52 | 0.22 | 298.81 | >0.01 |
| **73** | 3.27 | >0.01 | 10.41 | >0.01 | 0.96 | 0.33 | 9.72 | >0.01 |
| **74** | 0.02 | >0.01 | 826.67 | >0.01 | 0.01 | 0.93 | 147.93 | >0.01 |
| **75** | 64.40 | >0.01 | 130.18 | >0.01 | 1.70 | 0.19 | 49.06 | >0.01 |
| **76** | 65.74 | >0.01 | 1336.24 | >0.01 | 3.17 | 0.07 | 190.90 | >0.01 |
| **77** | 477.19 | >0.01 | 7960.71 | >0.01 | 25.29 | >0.01 | 543.76 | >0.01 |
| **78** | 51.72 | >0.01 | 2.26 | 0.13 | >0.01 | 0.97 | 0.23 | 0.63 |
| **79** | 36.39 | >0.01 | 1227.52 | >0.01 | 48.32 | >0.01 | 656.24 | >0.01 |
| **80** | 635.26 | >0.01 | 3830.58 | >0.01 | 31.50 | >0.01 | 693.15 | >0.01 |
| **81** | 7.61 | 0.01 | 49.12 | >0.01 | 5.78 | 0.02 | 69.88 | >0.01 |
| **82** | 14.00 | >0.01 | 720.70 | >0.01 | 5.26 | 0.02 | 19.58 | >0.01 |
| **83** | 258.75 | >0.01 | 574.76 | >0.01 | 38.01 | >0.01 | 139.49 | >0.01 |

***ΔABP = changes in arterial blood pressure, ΔICP = change in intracranial pressure, ΔrSO2 = change in regional cerebral oxygen saturation.***
